# Genetically predicted serum urate and cancer risk: A Mendelian randomization study

**DOI:** 10.1080/03009742.2025.2512667

**Published:** 2025-06-30

**Authors:** Tahzeeb Fatima, Mats Dehlin, Stephen Burgess, Amy M. Mason, Peter M Nilsson, Olle Melander, Lennart T. H. Jacobsson, Meliha C Kapetanovic

**Affiliations:** 1 Department of Rheumatology and Inflammation Research, University of Gothenburg, Gothenburg Sweden; 2 Department of Clinical Sciences Lund, Section of Rheumatology, Lund University, Lund Sweden; 3 MRC Biostatistics Unit, School of Clinical Medicine, University of Cambridge, Cambridge UK; 4 Cardiovascular Epidemiology Unit, School of Clinical Medicine, University of Cambridge, Cambridge UK; 5 British Heart Foundation Cardiovascular Epidemiology Unit, Department of Public Health and Primary Care, University of Cambridge, Cambridge UK; 6 Victor Phillip Dahdaleh Heart and Lung Research Institute, University of Cambridge, Cambridge UK; 7 Lund University, Department of Clinical Sciences Malmö, Malmö Sweden; 8 Lund University, Department of Clinical Sciences, Lund University, Lund Sweden; 9 Skåne University Hospital, Lund Sweden

## Abstract

**Objectives:**

Positive associations between urate levels and gout and the risk of some cancer types have been reported; however, whether the relationship is causal remains uncertain. We evaluate the causal effect of genetically predicted serum urate (SU) levels risk of overall and major site-specific cancers in individuals of European ancestry using Mendelian randomization (MR) analysis.

**Methods:**

Data from two population-based cohorts from southern Sweden, the Malmö Diet and Cancer Study (MDCS) and the Malmö Preventive Project (MPP) and summary-statistics data from the Global Urate Genetic Consortium (GUGC) and UK-Biobank cohort were used. A set of 26 SU-related variants was used as instrumental variables to perform a range of one (using MDCS-MPP) and two-sample (using GUGC and UK-Biobank) MR analyses. The causal relationship was assessed between genetically determined SU and 13 site-specific (bladder, breast, colorectal, gastric, hepatic, lung, pancreatic, prostate, renal, skin, lymphatic, hematopoietic, gynecological cancers, and brain tumor) and ‘any cancer’. We also performed epidemiological association analyses in individual-level data to determine SU-cancer relationship.

**Results:**

There was some suggestive evidence for an association between higher levels of genetically predicted SU and lower risk of brain (p=0.04; 1-sample MR) and colorectal (p=0.02; 2-sample MR) cancers, though these findings were not consistent across both MR approaches. No significant associations were observed between SU levels and the risk of other cancers (all *p* > 0.05).

**Conclusions:**

Our MR study found no consistent evidence of a causal effect of genetically predicted SU on overall or common site-specific cancers in individuals of European ancestry.

## Introduction

Serum urate (SU) is the end-product of purine degradation in humans and several genes are known to play a role in controlling its metabolism. Urate is a vital antioxidant that accounts for about 60% contribution to the respiratory and circulatory antioxidant activity in humans (1,2) which possibly could influence the risk for cancer (3, 4). Despite the beneficial role, high urate concentrations (hyperuricemia) could also have adverse health effects. Hyperuricemia is often defined by blood urate concentrations of >430 µmol/L in men and >360 µmol/L in women (5). A longstanding hyperuricemia can cause urate super-saturation in the blood and its crystallization in joints and soft tissues at serum concentrations above 405 µmol/L at 37 °C which can subsequently lead to the development of gout (6). The prevalence of hyperuricemia in the Western world is estimated to be 10 to 20% (7–9). At least 28 genetic loci conferring risk to develop hyperuricemia have been identified to date (10).

A number of diseases are associated with gout and hyperuricemia, including malignancies although causality remains to be established (11, 12). Associations between urate levels and the risk of cancer have been reported in a number of observational studies (13), and postulated to be the result of the intracellular oxidative action of urate (14). While few studies have reported a protective role of higher urate levels against some malignancies (15), other studies reported hyperuricemia being associated with increased risk of cancer (16). Such inconsistent results, despite the presence of many well-designed prospective studies (12, 17, 18), can be attributed to the biases that frequently arised in the observational studies due to unaccounted/unknown confounding.

Mendelian randomization (MR) is an analytical technique that takes into account the random assignment of alleles at the time of conception to disentangle the cause-effect relationship. The technique uses genetic variants known to be associated with exposure of interest as a proxy to detect the causal association with the outcome accounting for any confounding and reverse causality (19, 20). Several MR studies, using genetic variants in the urate transporter genes (*SCL2A9* and *ABCG2*) or combined genetic risk score from a list of urate-related variants as genetic instrument, have demonstrated a causal relationship between urate and multiple health outcomes (21). These and other studies provide evidence of both causal (e.g., T2D (22) and non-causal (e.g. chronic kidney disease (23)) relationship between genetically determined urate levels and defined phenotypes. Nevertheless, the available data regarding the causal relationship between urate and cancer risk remains limited.

Here, we designed a study to evaluate a causal effect of genetically predicted SU concentrations on cancer risks, overall and major cancer types, using a range of MR analytic approaches. The study also used observational data along with MR analysis to determine if the genetic data are consistent with the urate-cancer relationship detected in the observational association analysis. To keep the observational and MR association analyses in line, and owing to the availability of the data, the study was conducted only in individuals of European ancestry.

## Methods

### Study design

**Figure 1:**
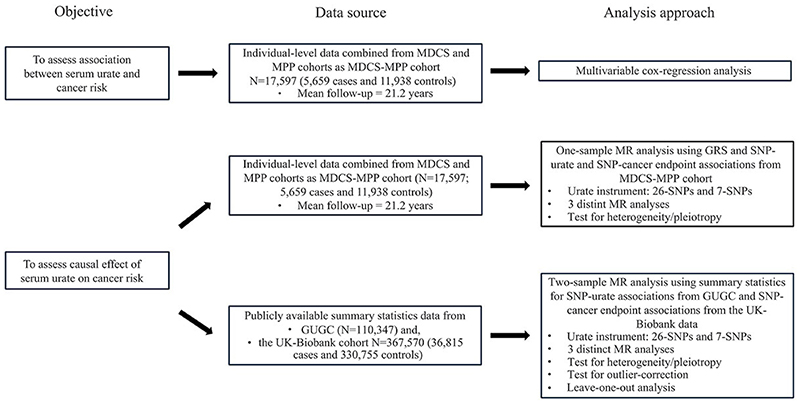
An overview of the study design. MDCS; Malmö Diet and Cancer Study, MPP; Malmö Preventive Project. Cases (MDCS-MPP) included canser and controls were free of cancer. GUGC; Global Urate Genetic Consortium, N; number of participants, GRS; genetic risk score, MR; Mendelian randomization, SNP; single nucleotide polymorphism/variants.

Figure 1 illustrates an overview of the study design, which comprised of several components including observational and causal association (or MR) analyses to comprehensively assess the relationship between urate and cancer in European individuals. We first performed an observational association analysis combining the data from two population-based Swedish cohorts from southernmost part of Sweden, the Malmö Diet and Cancer Study (MDCS) (24) and the Malmö Preventive Project (MPP) (25). The combined dataset is referred to as MDCS-MPP cohort in this study.

To ensure the robustness of causal associations, we implemented a range of, both primary and sensitivity, unidirectional MR analyses to determine the causal effect of urate on the risk of cancer. For this purpose, both one-sample and two-sample MR settings were employed using the individual-level data from MDCS-MPP and summary-statistics data from the Global Urate Genetic Consortium (GUGC) (10) and UK-Biobank cohort (26), respectively. The MR study was performed in accordance with Strengthening the Reporting of Observational Studies in Epidemiology using Mendelian Randomization (STROBE-MR) guidelines (27).

### Study participants and data sources

The observational association between urate and cancer risk was investigated using the data of 17,597 individuals in MDCS-MPP cohort (Table S1). Both MDCS and MPP are population-based cohorts of 30,447 and 33,346 European (mostly born in Sweden) participants, respectively. At baseline, individuals in MDCS underwent a health examination from 1991 to 1996, while those in MPP had a health examination from 1974 to 1992. Individuals from both of these cohorts, whose Genome-Wide Association Study (GWAS) data were available at the start of this study were included (n = 17,597 and mean age = 46.5 years, Table S2). The follow-up data descriptions and other details for MDCS and MPP have been published elsewhere (24, 25). For the purpose of this study, data for SU measurements (mean SU = 4.96 mg/dL) (Table S2) from the health surveys was used, and cancer endpoints (mean follow-up = 21.2 years) (Table S1) were obtained using the national cancer register in Sweden, which has been found to have a high validity (28–30). In total, data for 13 common site-specific cancers [bladder, breast, colorectal, gastric, hepatic, lung, pancreatic, prostate, renal, skin, lymphatic and hematopoietic cancers, gynecological (included ovarian, cervical and uterine cancers) cancers and brain tumor] were used, and the variable ‘any cancer’ was created by combining the cases from all 13 cancer types (n = 5,659) (Table S1). Cancer diagnosis was defined using International Classification of Diseases or ICD-9 and 10 codes (Table S3). To increase comparability, all cancer cases were excluded from the control group (n = 11,938).

For one-sample MR analysis, the data for SU measurement and that for 14 cancer outcomes (using data for each cancer type), were obtained from the MDCS-MPP cohort for all 17,597 participants. Among these, 5,659 who had cancer diagnosis were categorized as cases, while the remaining population without cancer development served as controls (n=11,938) in all analyses. For two-sample MR, we retrieved publicly available GWAS summary statistics data for variant-urate association for 110,347 European individuals from GUGC (10). The variant-cancer outcome association data for 13 site-specific and any cancer were derived from 367,570 European individuals in the UK-Biobank. Of these, 36,815 who were diagnosed with any of the studied cancer types were categorized as cases, and the remaining population without cancer served as controls (n=339,755) in all analyses. The details of the genotyping information in MDCS-MPP and UK-Biobank are provided in supplementary material (Text S1). The definitions for several cancer outcomes were similar but not identical between the MDCS-MPP and the UK-Biobank cohorts using ICD-9 and 10 codes (Table S3).

### Statistical analyses

#### Observational association analysis

For observational association analysis, multivariable cox-proportional hazard regression models, adjusted for age and sex (except for breast, prostate, and gynecological cancers, where the adjustment was done only for age), were used to evaluate the association of SU, separately, with the risk of each cancer type.

#### Instrument selection for MR analyses

We used a set of 26 variants (single nucleotide polymorphisms or SNPs), associated with SU at a genome-wide significance (*p* < 5 x 10^-08^) in the GUGC consortium (10), as the exposure instrument to run several MR analyses in this study (Table S4). Prior to conducting any MR analyses, we performed rigorous allele harmonization between the exposure (SU) and outcome (cancer) datasets. This involved removing palindromic SNPs, effect allele matching and ensuring the consistency in directionality i.e., all SNPs were coded such that the effect estimates reflected the allele associated with increased SU levels.

#### One-sample MR

We tested the association of all 26 SNPs with SU in the individual-level data from the MDCS-MPP cohort using linear regression models in controls only (Table S6 and S7). The F-statistic was also calculated in this regression analysis to assess the strength of the SU instrument. An F-statistic of > 10 is regarded as having a strong potential to predict causality without weak instrument bias (31). SNPs-to-cancer endpoint association estimates were obtained using logistic regression models (Table S8 to S21). All analyses were adjusted for age and sex, except for breast, prostate, and gynecological cancers, where the adjustment was done only for age.

To assess the robustness of causal associations, we performed a range of one-sample MR analyses using the summary-statistics from the above variant-urate and variant-cancer association analysis (wherever applicable) in the MDCS-MPP cohort. These analyses primarily implemented two-stage least square (2SLS) and inverse variance weighted (IVW) methods. Cochran’s Q test was used to assess the heterogeneity across the estimates for 26 SNPs. To ensure consistency in directionality, all SNPs were coded such that the effect estimates reflected the allele associated with increased SU levels in the cohort. We further applied MR analysis in MDCS-MPP data using weighted genetic risk score (GRS), where the GRS was calculated based on the number of urate-increasing alleles across all SNPs, weighted by their effect sizes on SU levels in the MDCS-MPP cohort.

#### Two-sample MR

To run two-sample MR, the summary statistics data for SNP/variant-urate associations were obtained from the GUGC consortium (Table S4 and S5) while for variant-cancer endpoints the data was obtained from the UK-Biobank (Table S22 to S35) for all 26 SNPs. The individual estimates for each SNP were generated using the Wald ratio estimator, and the standard errors (SE) were calculated using the Delta method (31, 32). The estimator provides the ratios for SNP-outcome estimate over SNP-exposure estimate. The individual estimates were then pooled for each cancer type in the IVW ratio method as the primary MR analysis using the random-effect model. All effects were adjusted for age and sex (wherever applicable) before running the MR analysis. Cochran’s Q test was used to assess the heterogeneity across the estimates for 26 SNPs. As IVW method is vulnerable to the horizontal pleiotropy through the confounding pathways independent of the exposure (SU in this case), a range of sensitivity analyses were carried out. Each of these sensitivity analyses assess the causal effect based on different assumptions that are designed to be less stringent to address the possible pleiotropy. The methods applied are weighted median and MR-Egger methods. The detailed description for these methods is provided in the supplementary material (Text S2). In addition, the Mendelian Randomization Pleiotropy Residual Sum and Outlier (MR-PRESSO) method was performed (33). The MR-PRESSO removes the potential outliers that are determined by the square of residual errors from the SNP-outcome against SNP-exposure regression to calculate an outlier-free effect estimate. This test is more sensitive than Egger and has the power to detect any outlier SNP in the MR analysis that could introduce biasness in the results. We also performed leave-one-out analyses to detect the significant effect that an individual SNP could render upon the MR estimates. Power calculations were done using mRnD power calculator (34).

To deal with any possible horizontal pleiotropy, an additional sensitivity analysis was performed. The 26 SNPs used as urate instrument were examined for their association with the traits other than SU levels using PhenoScanner (http://www.phenoscanner.medschl.cam.ac.uk/). The PhenoScanner is an online platform that provides public access to a vast range of GWAS summary results. We identified seven SNPs (Table S5 and S7) that were exclusively associated with SU levels (and or gout) and repeated all MR analyses using seven SNPs as urate instrument.

We applied Bonferroni correction to account for multiple testing for the 14 cancer outcomes. The associations with *p*-values < 3.5E-03 (where *p* = 0.05/14) were deemed as strong evidence for causal associations, while associations with *p*-values < 0.05 but > 3.5E-03 were arbitrated as suggestive evidence of associations. All analyses were done using R (v4.4.2) and Mendelian Randomization (35) and MR-PRESSO (33) packages in R.

#### Ethics approval and consent to participate

The Regional Ethics Committee at Lund University (Dnr LU 90-51, 85/2004 and 2009/633) provided ethical approval for this study in MDCS-MPP cohort. The UK Biobank has approval from the North West Multi-centre Research Ethics Committee (MREC) as a Research Tissue Bank (RTB) approval, renewed in 2021 (REC reference: 21/NW/0157).

## Results

### Observational association analysis in MDCS-MPP cohort

All hazard ratios (HRs) are presented as a change in the cancer risk per 1 SD increase in SU concentration (1 SD = 1.15 mgdL^-1^). We found a significant association between SU and an increased risk of colorectal cancer [HR (95%CI); 1.18 (1.09 to 1.28), p = 5.9E-05]. The urate was also associated with an increased risk of prostate cancer [HR (95%CI); 1.08 (1.02 to 1.15), p = 0.005] and gynecological cancers [HR (95%CI); 1.15 (1.01 to 1.32), p = 0.03]. While SU indicated susceptible association with most of the cancer risks (except gastric and lung cancer where the relationship was inverse), the results were not significant statistically. However, SU was associated with an increased risk of any cancer in both unadjusted and adjusted analyses [HR (95%CI); 1.06 (1.03 to 1.09), p = 1.18E-04]. The complete results for observational association analysis are provided in Table 1.

### One-sample MR analysis in MDCS-MPP cohort

**Figure 2:**
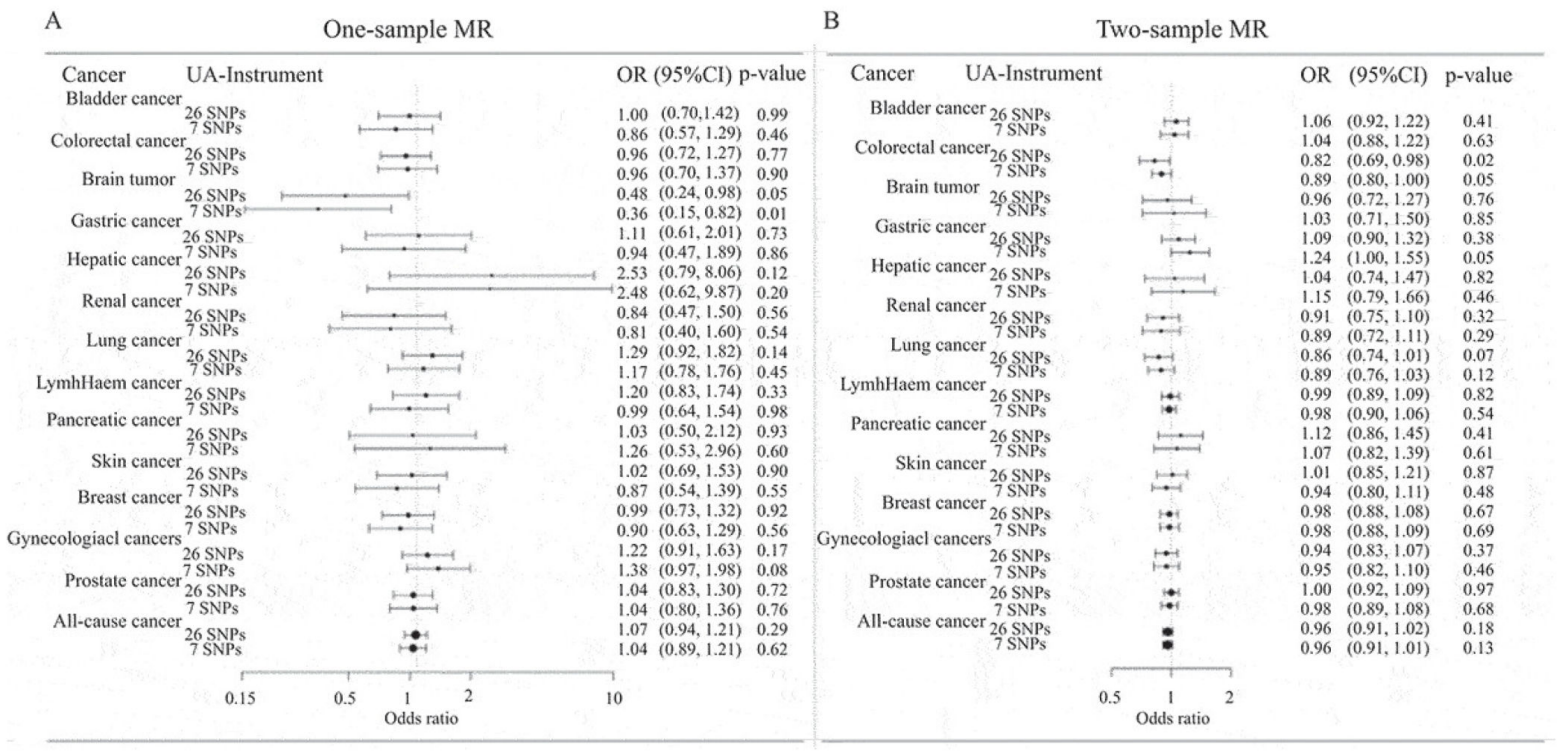
Forest plots illustrating results from MR analyses; A) results from one-sample MR analysis in MDCS-MPP cohort using 2-stage least square analysis approach and, B) results from two-sample MR analysis in GUGC-UK Biobank cohorts using inverse variance weighted (IVW) MR analysis approach. The results are presented for both 26 and 7-SNP urate instrument settings. All odds ratios (ORs) are presented as a change in the cancer risk per 1 mgdL^-1^ increase in SU concentration.

All 26 SNPs indicated an accumulative variance of 5.9% (R^2^ = 0.059) in SU concentrations in the MDCS-MPP cohort, which is comparable to 5.8% (R^2^ = 0.058) variance explained by these 26 SNPs in the GUGC cohort (10). The urate instrument also indicated an F-statistics of 31.38, suggesting that the instrument was unlikely to introduce marked weak instrument bias in the results. All odds ratios (ORs) in MR analyses are presented as a change in the cancer risk per 1 mgdL^-1^ increase in SU concentration. There was a consistent protective causal effect of urate on the risk of brain cancer in MDCS-MPP individuals in both 2SLS [OR (95%CI); 0.48 (0.24 to 0.98), p = 0.05] (Figure 2A) and GRS [OR (95%CI); 0.48 (0.24 to 0.98), p = 0.04] MR analyses, while the IVW estimates were not significant and indicated heterogeneity [OR (95%CI); 0.48 (0.17 to 1.41), p = 0.18, p_het_ = 0.04] in 26-SNP MR analysis (Table 2).

Restricting the urate instrument criteria to 7-SNPs showed a suggestive protective effect of urate on brain cancer [2SLS; OR (95%CI); 0.36 (0.15 to 0.82), p = 0.01 (Figure 2A) and GRS; OR (95%CI); 0.38 (0.17 to 0.86), p = 0.02, IVW; OR (95%CI); 0.36 (0.15 to 0.87), p = 0.02] with non-significant heterogeneity (p_het_ = 0.07) (Table 2). SU also indicated a consistent trend towards a significant causal effect on an increased risk of gynecological cancers in restricted (7-SNPs) analyses. Complete results for one-sample MR are presented in Figure 2A and Table 2.

### Two-sample MR analysis in publicly available GWAS

All 26 SNPs had F-statistic values larger than 10 (with lowest being 30.1) (Table S4), that indicated that the chance of introducing weak instrument bias in two-sample MR analyses was unlikely. The individual MR estimates for each SNP are illustrated in Figure S1 while the detailed results for two-sample MR are presented in Figure 2B and Table 3, where all ORs are presented as a change in the cancer risk per 1 mgdL^-1^ increase in SU concentration. The main MR analysis revealed a suggested causal effect of higher urate on the decreased risk of colorectal cancer with high heterogeneity [OR (95%CI); 0.82 (0.69 to 0.98), p = 0.02, p_het_ < 0.0001] (Figure 2B). Restricting the instrument criteria to 7-SNP set-up still indicated a significant effect of urate for a decreased risk of colorectal cancer, with no heterogeneity [OR (95%CI); 0.89 (0.79 to 1.00), p = 0.05, p_het_ = 0.74] (Figure 2B). The outlier removal via MR-PRESSO still resulted in a suggestive effect [OR (95%CI); 0.82 (0.68 to 0.97), p = 0.04] (Table 3). The only other cancer for which urate indicated a trend of association was lung cancer with significant heterogeneity [OR (95%CI); 0.86 (0.73 to 1.02), p = 0.07, p_het_ = 0.04] (Figure 2B). There was no association between urate and any other cancer risk and results were consistent throughout the range of MR and sensitivity analyses that were implied in two-sample setting. None of the leave-one-out analyses showed a particular SNP to be an outlier and having a significant effect on MR estimates (Figure S2). Results from power calculations for both one- and two-sample MR are detailed in Table S36.

## Discussion

In this population-based study, the MR analysis did not provide strong evidence to support a causal role of urate levels in cancer incidence. While there was evidence supporting a potential causal effect of higher urate on the decreased risk of colorectal cancer and brain tumor, these findings were only rendered as being suggestive as the significance diminished after correction for multiple testing and these findings were not consistent in the two populations studied. Among previous studies addressing this subject, one study using the Copenhagen General Population Study demonstrated higher plasma urate to be causally associated with cancer incidence (OR = 1.22) and all-cause mortality (OR = 1.49) using only *rs7442295* in *SLC2A9* gene as an instrument for urate (36), while one showed causal association of higher urate with higher risk of prostate cancer in Japanese population using 34-SNP urate instrument (37). Contrary to these, other studies did not report any causality of urate levels on neither colorectal (38) nor eight cancer outcomes (39) using 26-SNP urate instrument and several publicly available cancer datasets. Similarly, no evidence of a causal relationship between urate and lung cancer was reported using UK-Biobank data in one-sample MR setting (40). However, they did not adjust for potential confounding covariates while calculating variant-urate and variant-cancer risk associations for several MR analyses.

Several reports suggest that urate might potentially offer protection against cancer in humans due to its remarkable antioxidant properties (2, 4, 14, 41). However, persistently higher levels of urate have also been associated with inflammation and development of gout (8), and thereby set a question mark for its protective effect against cancer. In a study of middle-aged men in Sweden (Malmö), cancer mortality was positively associated with SU levels only in the first 2.5 years after urate screening (42). The Swedish Apolipoprotein-related MOrtality RISk study (AMORIS) including 493,281 adults identified a correlation between elevated SU and an increased risk of colorectal, hepatobiliary, kidney, and non-melanoma skin cancers in men, and head and neck cancers in women but an inverse relationship between SU levels and the risk of pulmonary cancers in men, along with lymphatic, hematological, and breast cancers in women (43). Moreover, a positive association between SU and prostate cancer incidence was observed in Japanese men in Hawaii, but it attenuated over time and became non-significant >10 years after the urate measurements (18). A recent prospective population-based study in EPIC-Heidelberg cohort reported higher levels of SU to be associated with lower risk of breast cancer but no evidence of any association between SU and risk of other common cancers i.e., prostate, colorectal and lung cancers (44). These findings align with the observational association analysis results in our study, where we observed both protective and susceptible relationship between higher SU levels and risk of a few cancer types, while for others the analyses showed no association at all even after adjustments for age and sex as primary confounders (Table 1).

In MDCS-MPP cohort, using one-sample MR approach, higher SU indicated an association with reduced risk of brain cancer in the Swedish population, although, the number of cases was limited. Given the brain’s lower antioxidant capacity, it is considered particularly vulnerable to oxidative stress, and urate has been suggested to play a neuroprotective role (45). In slight contrast to these suggestive findings, a meta-analysis of three prospective cohort studies reported no association between brain cancer risk and gout. An explanation to this apparent discrepancy could be that clinical gout represent a pro-inflammatory phenotype and occurs in only minority of subjects with hyperuricemia (46). It is important to note here that in MDCS-MPP cohort in our study *rs7193778* showed the strongest link to brain cancer risk. This association may represent an incidental finding given its pleiotropic effect and small overall impact on SU and should be interpreted with caution in the context of multiple testing.

Our study also demonstrated suggestive evidence for a protective role of SU against colorectal cancer in publicly available larger cohorts (GUGC and the UK-Biobank in two-sample MR settings), albeit with high heterogeneity. We, however, did not observe a causal effect of urate in changing the risk of colorectal cancer in Swedish population. The previous MR analyses were not able to report a causal effect of urate on colorectal cancer using the GUGC, UK-Biobank and other publicly available data in several European cohorts (38, 39). One possible explanation for these divergent results could be our exclusion of patients developing any cancer types from the control group that was used for all comparisons and calculating variant-urate associations in MR analyses. We also accounted for adjustments for age and sex as potential confounders for urate while calculating urate-cancer associations in the UK-Biobank cohort. In their study, Jiang et al. (2021) (39) also reported one variant *rs 12498742*, to be significantly affecting the MR estimates in leave-one-out analysis, which is line with our results (Figure S2). However, the association sustained in our analysis when corrected for this and other outlier variants via MR-PRESSO (OR = 0.82, p = 0.04, Table 3). Since *rs12498742* (in *SLC2A9* gene) is so far the strongest urate-associated variant reported in European GWAS (10), it would be undesirable to not select this variant as a proxy to reflect serum urate levels in humans in any MR analysis. In addition, we did find a similar protective association of urate for colorectal cancer risk when using variants strictly associated with urate and gout as urate instrument only in two-sample MR (OR 0.89, p =0.04, Table 3). Data in most observational studies have reported a possible association of high urate levels and increased risk of colorectal cancer (47, 48). Our observational analysis also indicating an association between higher urate levels and an increased risk of developing colorectal cancer (OR 1.18, p= 5.9E-05, after adjustment for age and sex), which is in line with these studies but could include major effects of residual confounding, a problem which is largely avoided using genetically predicted urate levels as the exposure. In contrast, a study that reported 10-year prevalence of colorectal cancer in gout verses osteoarthritis patients, using the computerized patient record system of the VA New York Harbor Health Care System, reported that individuals with gout had significantly lower (0.8%) prevalence of colorectal cancer than those with osteoarthritis (3.7%) (p = 0.0008) (49). Furthermore, Mi et al. (2022) reported a U-shaped relationship between serum urate and the risks of colon and rectal cancers i.e., the higher risks for both cancer types in the lowest (≤3.5 mgdL^-1^) and highest (>8.4 mgdL^-1^) serum urate groups compared to the reference group (>3.5-5.4 mgdL^-1^) (50). This suggests that the association between SU and colorectal cancer is more complex than a simple linear relationship, and given the known common genetic variants explain only a minor proportion of the variance (23.9%) in SU levels, the inconsistent MR estimates through a range of MR analyses in our study merits further exploration.

The study has several strengths. First, it investigates the observational association between SU levels and cancer risk using a comprehensive list of cancer outcomes in observational analysis in the Swedish (MDCS-MPP) cohort. Second, it applied one-sample and two-sample MR analyses in three independent European cohorts to avoid overlapping, hence improving the reliability of the results. Third, the study prevented competing risk via excluding the patients for any cancer type from the control group altogether. Fourth, we adjusted for common population covariates, age and sex, in all MR analyses to account for their possible association with urate levels. To the best of our knowledge, the study presents the largest MR-analyses performed so far in individuals of any ancestry to investigate SU to cancer risk causality with most of cancer outcomes included in the analysis. Moreover, the study used a range of robust MR-analyses methods to account for possible violations of the MR assumptions, such as horizontal pleiotropy.

The first limitation of this study is lower statistical power for several MR analyses, particularly for rarer cancer types, where a consistent lower case to control proportions for several cancer types may have largely contributed. In addition, the GWAS data for SU and cancer outcomes were obtained from heterogenous populations and it is possible that population differences for potentially important features, other than age and sex that we adjusted for, may have contributed to masking the causal effect of urate on cancer. Moreover, our investigation (observational and MR analyses) was confined to the European population, thereby limiting the generalizability of these findings to other population settings.

### Conclusion

This study did not find statistically significant and consistent evidence to support a causal role of genetically predicted SU levels for neither overall nor site specific cancer risks, although there were indications for lower risks for colorectal and brain cancer with higher genetically determined urate levels, which warrants further investigation in larger cohorts as well as in other population settings. Moreover, MR investigation(s) in data particularly well stratified for SU levels for SNP-urate and SNP-cancer endpoint associations may be helpful in delineating a potential non-linear relationship between SU and cancer risk.

## Supplementary Material

Supplementary material

## Figures and Tables

**Table 1:** Urate association with cancers in MDCS-MPP cohort

Cancer type	Unadjusted		Adjusted*	
	HR (95%CI)	*p*-value	HR (95%CI)	*p*-value
Bladder	0.97 (0.88; 1.08)	0.65	1.08 (0.98; 1.21)	0.12
Colorectal	0.98 (0.92; 1.06)	0.71	1.18 (1.09; 1.29)	5.9E-05
Brain	0.90 (0.75; 1.09)	0.27	1.16 (0.93; 1.44)	0.17
Gastric	0.82 (0.69; 0.99)	0.04	0.92 (0.76; 1.11)	0.36
Hepatic	1.04 (0.77; 1.40)	0.81	1.01 (0.76; 1.32)	0.97
Renal	0.89 (0.78; 1.03)	0.13	1.01 (0.86; 1.19)	0.92
Lung	0.85 (0.78; 0.93)	5.6E-04	0.94 (0.86; 1.04)	0.21
Lymphatic and hematopoietic	0.90 (0.82; 1.00)	0.04	1.02 (0.92; 1.13)	0.71
Pancreatic	0.97 (0.81; 1.19)	0.83	1.09 (0.89; 1.34)	0.40
Skin	0.85 (0.77; 0.95)	0.005	1.03 (0.91; 1.17)	0.60
Breast	1.13 (1.03; 1.24)	0.008	1.02 (0.93; 1.13)	0.62
Gynecological	1.16 (1.02; 1.32)	0.02	1.15 (1.01; 1.32)	0.03
Prostate	1.06 (1.01; 1.12)	0.04	1.08 (1.02; 1.15)	0.005
Any cancer	0.86 (0.84; 0.88)	<2E-16	1.06 (1.03; 1.09)	1.2E-04

All hazard ratios (HRs) are presented as a change in the cancer risk per 1 SD increase in SU concentration (1 SD = 1.15 mg/dL). SD: standard deviation, MDCS-MPP: Malmö Diet Cancer and Malmö Preventive Project

* Values are adjusted for age and sex, except for breast, prostate, and gynecological cancers where values are adjusted only for age.

**Table 2:** Results of one-sample MR (using MDCS-MPP cohort) for causal relationship between serum urate levels and cancer risk

Cancer type	MR instrument	Cochran’s Q test		GRS		IVW	
		*Q*	*p*-Het	OR (95% CI)	*p*-value	OR (95% CI)	*p*-value
Bladder	26 SNPs	23.47	0.55	0.94 (0.67; 1.34)	0.76	1.00 (0.71; 1.42)	0.80
	7 SNPs	2.71	0.84	0.82 (0.56; 1.23)	0.35	0.85 (0.57; 1.29)	0.46
Colorectal	26 SNPs	25.35	0.44	0.97 (0.73; 1.29)	0.83	0.96 (0.72; 1.28)	0.80
	7 SNPs	2.99	0.81	0.98 (0.71; 1.37)	0.94	0.97 (0.70; 1.37)	0.89
Brain	26 SNPs	38.75	0.04	0.48 (0.24; 0.98)	0.04	0.48 (0.17; 1.41)	0.18
	7 SNPs	11.52	0.07	0.38 (0.17; 0.87)	0.02	0.36 (0.15; 0.87)	0.02
Gastric	26 SNPs	26.62	0.37	1.05 (0.59; 1.90)	0.86	1.08 (0.59; 2.00)	0.78
	7 SNPs	6.07	0.41	0.89 (0.46; 1.77)	0.74	0.92 (0.45; 1.91)	0.83
Hepatic	26 SNPs	29.43	0.25	2.57 (0.82; 8.26)	0.11	2.67 (0.81; 8.82)	0.11
	7 SNPs	7.52	0.27	2.32 (0.62; 9.10)	0.22	2.83 (0.67; 11.9)	0.15
Renal	26 SNPs	15.92	0.92	0.82 (0.46; 1.47)	0.5	0.85 (0.48; 1.52)	0.59
	7 SNPs	0.99	0.98	0.80 (0.41; 1.58)	0.52	0.83 (0.42; 1.63)	0.58
Lung	26 SNPs	16.19	0.91	1.26 (0.90; 1.78)	0.18	1.29 (0.93; 1.82)	0.13
	7 SNPs	2.24	0.89	1.16 (0.79; 1.17)	0.43	1.17 (0.79; 1.75)	0.42
Lymphatic and hematopoietic	26 SNPs	19.98	0.74	1.18 (0.04; 1.18)	0.37	1.20 (0.83; 1.75)	0.33
	7 SNPs	3.99	0.67	0.99 (0.65; 1.53)	0.98	0.99 (0.64; 1.56)	0.99
Pancreatic	26 SNPs	33.08	0.13	1.04 (0.51; 2.13)	0.91	1.15 (0.58; 2.31)	0.68
	7 SNPs	10.62	0.10	1.22 (0.54; 2.85)	0.63	1.45 (0.64; 3.30)	0.37
Skin	26 SNPs	22.01	0.63	1.00 (0.67; 1.49)	0.99	0.99 (0.67; 1.48)	0.99
	7 SNPs	6.96	0.32	0.85 (0.54; 1.36)	0.5	0.84 (0.53; 1.35)	0.48
Breast	26 SNPs	33.58	0.12	0.98 (0.74; 1.32)	0.94	0.99 (0.74; 1.33)	0.95
	7 SNPs	2.24	0.89	0.89 (0.64; 1.26)	0.53	0.89 (0.63; 1.28)	0.55
Gynecological	26 SNPs	25.48	0.43	1.18 (0.89; 1.58)	0.23	1.22 (0.91; 1.63)	0.18
	7 SNPs	1.43	0.96	1.33 (0.95; 1.87)	0.09	1.36 (0.97; 1.94)	0.07
Prostate	26 SNPs	17.78	0.85	1.04 (0.84; 1.30)	0.69	1.06 (0.85; 1.33)	0.57
	7 SNPs	2.58	0.85	1.02 (0.80; 1.33)	0.82	1.05 (0.81; 1.36)	0.69
Any cancer	26 SNPs	22.51	0.61	1.07 (0.94; 1.21)	0.29	1.07 (0.95; 1.22)	0.27
	7 SNPs	1.29	0.97	1.04 (0.91; 1.21)	0.56	1.04 (0.90; 1.21)	0.61

All odds ratios (ORs) are presented as a change in the cancer risk per 1 mg/dL increase in SU concentration. MR: Mendelian randomization, MDCS-MPP: Malmö Diet Cancer and Malmö Preventive Project, 95% CI: 95% confidence interval, 2SLS: two-stage least square, GRS: genetic risk score, IVW: Inverse variance weighted method, *Q*; *Q* value from Cochran’s *Q*-test where higher value indicates higher heterogeneity, *p*; *p*-value for the given analysis.

**Table 3:** Results from two-sample MR (using GUGC and the UK Biobank cohort) for causal relationship between serum urate levels and cancer risk

Cancer type	MR instrument	Cochran’s *Q* test		Weighted median		MR-Egger		Egger- intercept		MR-PRESSO				
										Raw		No. of outilers	Outlier corrected	
		*Q*	*p*	OR (95% CI)	*p*	OR (95% CI)	*p*	Est.	*p*	Raw OR (95% CI)	*p*		OR (95% CI)	*p*
Bladder	26 SNPs	20.32	0.72	1.03 (0.86; 1.23)	0.73	0.97 (0.80; 1.19)	0.79	0.011	0.24	1.06 (0.92; 1.22)	0.37	0	NA	NA
	7 SNPs	4.57	0.59	1.02 (0.86; 1.23)	0.75	1.03 (0.82; 1.29	0.79	0.002	0.89	1.04 (0.88; 1.23)	0.6	0	NA	NA
Colorectal	26 SNPs	75.77	< 0.000 1	0.89 (0.79; 1.01)	0.06	0.92 (0.72; 1.18)	0.51	-0.014	0.20	0.82 (0.69; 0.98)	0.03	4	0.82 (0.69; 0.98)	0.0 4
	7 SNPs	3.49	0.74	0.89 (0.79; 1.01)	0.07	0.89 (0.76; 1.04)	0.15	0.001	0.96	0.89 (0.80; 1.01)	0.04	0	NA	NA
Brain	26 SNPs	27.21	0.34	1.08 (0.77; 1.54)	0.63	1.08 (0.72; 1.64)	0.68	-0.016	0.38	0.95 (0.72; 1.27)	0.76	0	NA	NA
	7 SNPs	8.41	0.21	1.10 (0.78; 1.54)	0.57	1.23 (0.74; 2.06)	0.42	-0.039	0.32	1.03 (0.71; 1.50)	0.86	0	NA	NA
Gastric	26 SNPs	25.93	0.41	1.18 (0.93; 1.52)	0.16	1.19 (0.91; 1.58)	0.21	-0.012	0.35	1.09 (0.89; 1.32)	0.39	0	NA	NA
	7 SNPs	5.17	0.52	1.19 (0.94; 1.53)	0.15	1.26 (0.93; 1.72)	0.13	-0.004	0.86	1.24 (1.00; 1.55)	0.08	0	NA	NA
Hepatic	26 SNPs	33.02	0.13	0.99 (0.68; 1.45)	0.98	1.22 (0.74; 1.10)	0.43	-0.02	0.37	1.04 (0.74; 1.41)	0.82	0	NA	NA
	7 SNPs	6.76	0.34	1.12 (0.77; 1.64)	0.54	1.03 (0.61; 1.78)	0.88	0.022	0.59	1.14 (0.79; 1.66)	0.49	0	NA	NA
Renal	26 SNPs	18.09	0.83	0.90 (0.71; 1.15)	0.4	0.88 (0.67; 1.15)	0.36	0.004	0.75	0.91 (0.76; 1.09)	0.25	0	NA	NA
	7 SNPs	2.64	0.85	0.89 (0.70; 1.13)	0.35	0.82 (0.61; 1.11)	0.19	0.017	0.44	0.89 (0.72; 1.11)	0.16	0	NA	NA
Lung	26 SNPs	38.15	0.04	0.86 (0.74; 1.02)	0.09	0.84 (0.67 ; 1.07)	0.15	0.003	0.76	0.86 (0.74; 1.02)	0.08	0	NA	NA
	7 SNPs	4.67	0.58	0.87 (0.74; 1.03)	0.11	0.79 (0.64; 0.97)	0.02	0.026	0.1	0.88 (0.76; 1.03)	0.13	0	NA	NA
Lymphatic and hematopoietic	26 SNPs	54.28	0.000 6	0.96 (0.89; 1.05)	0.41	0.93 (0.81; 1.08)	0.35	0.007	0.28	0.98 (0.89; 1.09)	0.82	1	0.99	0.9 9
	7 SNPs	4.97	0.54	0.96 (0.89; 1.05)	0.41	0.95 (0.85; 1.06)	0.36	0.006	0.48	0.97 (0.90; 1.06)	0.53	0	NA	NA
Pancreatic	26 SNPs 7 SNPs	32.44 2.69	0.14 0.84	1.07 (0.81; 1.44) 1.07 (0.81; 1.43)	0.61 0.61	1.07 (0.73; 1.57) 1.04 (0.73; 1.51)	0.72 0.79	0.005 0.005	0.76 0.86	1.11 (0.86; 1.45) 1.07 (0.82; 1.40)	0.41 0.48	0 0	NA NA	NA NA
Skin	26 SNPs	39.76	0.03	0.93 (0.78; 1.11)	0.44	0.91 (0.71; 1.18)	0.49	0.013	0.26	1.01 (0.85; 1.21)	0.87	0	NA	NA
	7 SNPs	5.63	0.46	0.92 (0.78; 1.10)	0.4	0.91 (0.72; 1.15)	0.42	0.008	0.65	0.94 (0.80; 1.11)	0.5	0	NA	NA
Breast	26 SNPs	57.24	0.000 2	1.00 (0.92; 1.09)	0.99	0.94 (0.82; 1.10)	0.46	0.004	0.54	0.97 (0.88; 1.08)	0.67	1	0.98 (0.88; 1.08)	0.8 1
	7 SNPs	11.94	0.06	0.99 (0.90; 1.08)	0.76	0.93 (0.80; 1.09)	0.41	0.01	0.42	0.97 (0.87; 1.09)	0.7	0	NA	NA
Gynecologic al	26 SNPs 7 SNPs	23.24 3.13	0.56 0.79	0.94 (0.80; 1.11) 0.94 (0.80; 1.11)	0.5 0.47	0.92 (0.77; 1.11) 0.91 (0.74; 1.12)	0.4 0.36	0.003 0.009	0.75 0.57	0.94 (0.83; 1.07) 0.94 (0.81; 1.)	0.37 0.35	0 0	NA NA	NA NA
Prostate	26 SNPs	20.51	0.71	0.98 (0.88; 1.10)	0.77	0.94 (0.84; 1.07)	0.37	0.007	0.19	1.00 (0.92; 1.09)	0.96	0	NA	NA
	7 SNPs	4.16	0.65	0.97(0.87; 1.08)	0.6	0.96 (0.84; 1.10)	0.56	0.004	0.68	0.97 (0.88; 1.09)	0.64	0	NA	NA
Any cancer	26 SNPs	62.4	< 0.000 1	0.97 (0.93; 1.02)	0.21	0.93 (0.86; 1.02)	0.11	0.004	0.35	0.96 (0.91; 1.02)	0.19	1	0.96 (0.91;1.02)	0.2 1
	7 SNPs	9.85	0.13	0.97 (0.92; 1.02)	0.22	0.93 (0.87; 1.00)	0.06	0.006	0.26	0.95 (0.89; 1.01)	0.18	0	NA	NA

All odds ratios (ORs) are presented as a change in the cancer risk per 1 mgdL^-1^ increase in SU concentration. MR: Mendelian randomization, GUGC: Global Urate Genetic Consortium, MR-PRESSO; Mendelian Randomization Pleiotropy Residual Sum and Outlier, Est.; Estimate for Egger intercept, 95% CI: 95% confidence interval, *Q*; *Q* value from Cochran’s *Q*-test where higher value indicates higher heterogeneity, *p*; *p*-value for the given analysis.

## Data Availability

The data for MDCS and MPP cohorts can be obtained via reasonable request, while the data used for two-sample MR analysis are publicly available.
