# Supplementary material for: Genetically predicted serum urate and cancer risk: A Mendelian randomization study
Source: Scand J Rheumatol. Author manuscript; Available in PMC 2025 Sep 23. (PMC7618153; doi:10.1080/03009742.2025.2512667)
Supplement: Supplementary material [file EMS207593-supplement-Supplementary_material.pdf]

Supplementary texts

Text S1: Genotyping information

In the MDCS-MPP cohort, DNA genotyping was done via a MALDI-TOF mass spectrometer (Sequenom MassArray, Sequenom, San Diego, CA, USA) using Sequenom reagents and protocols. For a candidate SNP that failed genotyping, a proxy SN P, identified using SNAP version 2.2.2, was used. For the SNPs failing the Sequenom genotyping, TaqMan, KASPar allelic discrimination on an ABI 7900HT (Applied Biosystems, Life Technologies, Carlsbad, CA, USA) genotyping was used as per manufacturer's instructions. Complete details of genotyping in MDCS-MPP cohort are provided in Teleka et al. (2020) [1].

In the UK-Biobank cohort, the DNA genotyping was performed on two similar purpose-designed arrays: UK BiLEVE Axiom array (~50,000 participants) and UK Biobank Axiom array (~450,000 participants). These arrays share 95% marker content [2]. The resulting dataset of 489,212 samples at 812,428 unique markers was quality controlled and imputed to give results for ~96M genotypes.

Text S2: MR-Egger and Weighted median methods

We estimated the intercept and slope of MR-Egger regression, which represent the average horizontal pleiotropy and a pleiotropy-adjusted MR estimate, respectively. An intercept value for this regression that does not include the null ($p < 0.05$) was considered indicative of horizontal pleiotropy. MR-Egger assumes that the strength of the instruments is not correlated to any pleiotropic effect that they have. Additionally, the weighted median estimator was performed. This is the weighted median effect of all the MR estimates produced by individual instruments, with weights equal to the inverse of the standard error. It is valid when more than half the information from the analysis comes from valid instruments.

Supplementary tables

Table S1: Number of controls and cancer cases in MDCS-MPP and UK Biobank cohorts

Cancer type		MDCS-MPP				UK Biobank		
		**Follow-up (years)**	**All total**	**Case**	**Control**	**All total**	**Case**	**Control**
1	Bladder	25.81	12454	516	11938	333000	2245	330755
2	Colorectal	24.98	12729	791	11938	335243	4488	330755
3	Brain	17.58	12054	116	11938	331344	589	330755
4	Gastric	25.06	12114	176	11938	331955	1200	330755
5	Hepatic	27.55	11987	49	11938	331241	486	330755
6	Renal	24.35	12115	177	11938	331994	1239	330755
7	Lung	25.31	12483	545	11938	333345	2590	330755
8	Lymphatic and hematopoietic	24.64	12399	461	11938	340332	9577	330755
9	Pancreatic	24.47	12060	122	11938	331590	835	330755
10	Skin	21.32	12331	393	11938	333019	2264	330755
11	Breast	17.4	5364	729	4635	189222	10274	178948
12	Gynecological	15.36	5000	368	4635	181590	2642	178948
13	Prostate	26.2	8827	1521	7306	158281	6474	151807
14	All-cause	21.19	17597	5659	11938	367570	36815	330755

MDCS-MPP: Malmö Diet Cancer Study and Malmö Preventive Project

Table S2: Basic characteristics of individuals in MDCS-MPP cohort (n = 17597)

Characteristics	N (%)	Mean ± SD
Sex, male	10639 (60.4)	-
Age (all)	17597 (100)	46.53 ± 6.6
Age (Males)	10639 (60.4)	44.11 ± 5.2
Age (Females)	6958 (39.5)	50.22 ± 6.8
BMI (kg/m2)	17590 (99.9)	24.43 ± 3.5
Serum creatinine (µmol/L)	17530 (99.6)	86.93 ± 18.2
Serum urate (µmol/L)	17544 (99.7)	294.89 ± 68.2
Serum urate (mg/dL)	17544 (99.7)	4.96 ± 1.15
Serum urate (Males) (µmol/L)	10619 (60.3)	321.69 ± 62.0
Serum urate (Females) (µmol/L)	6928 (39.3)	253.84 ± 55.6

MDCS-MPP: Malmö Diet Cancer Study and Malmö Preventive Project, BMI: body mass index, SD: standard deviation

Table S3: ICD codes for cancer diagnosis that were matched between the MDCS-MPP and UK Biobank cohort prior to the analyses

	Cancer type	ICD-9 codes	ICD-10 codes
1	Bladder Cancer	188.X	C67.X
2	Colorectal Cancer	153.X, 154.0, 154.1, 154.2, 154.8, 159.0, 159.9	C18.X, C20.X
3	Brain Tumor	191.X	C71.X
4	Gastric Cancer	150.X, 151.X	C15.X, C16.X
5	Hepatic Cancer	155.X	C22.X
6	Renal Cancer	189.X	C64.X, C65.X, C66.X, C68.X
7	Lung Cancer	162.X	C34.X
8	Lymphatic and Hematopoietic Cancer	200.X, 201.X, 202.X, 203.X, 204.X, 205.X, 206.X, 207.X, 208.X, 209.X	C77.X, C81.X, C82.X, C83.X, C84.X, C85.X, C86.X, C88.X, C90.X, C91.X, C92.X, C93.X, C94.X, C95.X, C96.X
9	Pancreatic Cancer	157.X	C25.X
10	Skin Cancer	172.X	C43.X
11	Breast Cancer	174.X	C50.X
12	Gynecological Cancers	179.9, 180.X, 181.9, 182.0, 182.1, 183.X, 184.9, 195.3	C51.X, C52.X, C53.X, C54.X, C55.X, C56.X, C57.X, C58.X
13	Prostate Cancer	185.X	C61.X
14	All-cause cancer	150.X, 151.X, 153.X, 154.0, 154.1, 154.2, 154.8, 155.X, 157.X, 159.0, 159.9, 162.X, 172.X, 174.X, 179.9, 180.X, 181.9, 182.0, 182.1, 183.X, 184.9, 185.X, 188.X, 189.X, 191.X, 195.3, 200.X, 201.X, 202.X, 203.X, 204.X, 205.X, 206.X, 207.X, 208.X, 209.X	C15.X, C16.X, C18.X, C20.X, C22.X, C25.X, C34.X, C43.X, C50.X, C51.X, C52.X, C53.X, C54.X, C55.X, C56.X, C57.X, C58.X, C61.X, C64.X, C65.X, C66.X, C67.X, C68.X, C71.X, C77.X, C81.X, C82.X, C83.X, C84.X, C85.X, C86.X, C88.X, C90.X, C91.X, C92.X, C93.X, C94.X, C95.X, C96.X

ICD: International Classification of Diseases, MDCS-MPP: Malmö Diet Cancer Study and Malmö Preventive Project

Table S4: List of 26 SNPs showing SNP-urate association results in GUGC cohort (Köttgen et al. [3]) used as instruments in MR

	SNP	Chr	Closest gene	EA	OA	EAF	GX	SE-GX	*p*	N	MAF	R2	F-statistics
1	*rs11264341**	1	*TRIM46*	C	T	0.57	-0.05	0.006	6.2 E-19	110347	0.43	0.00062893	69.3988818
2	*rs1471633*	1	*PDZK1*	A	C	0.46	0.059	0.005	1.2 E-29	110347	0.46	0.00126025	139.060742
3	*rs1260326*	2	*GCKR*	T	C	0.41	0.074	0.005	1.2 E-44	110347	0.41	0.00198108	218.600121
4	*rs17050272*	2	*INHBB*	A	G	0.43	0.035	0.006	1.6 E-10	110347	0.43	0.00030828	34.016363
5	*rs6770152**	3	*SFMBT1*	G	T	0.42	-0.044	0.005	2.6 E-16	110347	0.42	0.00070129	77.3835879
6	*rs12498742*	4	*SLC2A9*	A	G	0.77	0.373	0.006	< 1 E-700	110347	0.23	0.03383799	3733.81943
7	*rs2231142*	4	*ABCG2*	T	G	0.11	0.217	0.009	1.0 E-134	110347	0.11	0.00524073	578.28328
8	*rs17632159**	5	*TMEM171*	G	C	0.69	-0.039	0.006	3.5 E-11	110347	0.31	0.00038274	42.2326812
9	*rs1165151**	6	*SLC17A1*	G	T	0.53	-0.091	0.005	7.0 E-70	110347	0.47	0.00299282	330.23968
10	*rs675209*	6	*RREB1*	T	C	0.27	0.061	0.006	1.3 E-23	110347	0.27	0.00093582	103.261577
11	*rs729761**	6	*VEGFA*	G	T	0.7	-0.047	0.006	8.0 E-16	110347	0.3	0.00055577	61.3253415
12	*rs10480300*	7	*PRKAG2*	T	C	0.28	0.035	0.006	4.1 E-9	110347	0.28	0.00030828	34.016363
13	*rs1178977*	7	*BAZ1B*	A	G	0.81	0.047	0.007	1.2 E-12	110347	0.19	0.00040838	45.0619972
14	*rs17786744**	8	*STC1*	G	A	0.42	-0.029	0.005	1.4 E-8	110347	0.42	0.00030476	33.6288335
15	*rs2941484*	8	*HNF4G*	T	C	0.44	0.044	0.005	4.4 E-17	110347	0.44	0.00070129	77.3835879
16	*rs10821905*	10	*A1CF*	A	G	0.18	0.057	0.007	7.4 E-17	110347	0.18	0.00060053	66.2645023
17	*rs1171614**	10	*SLC16A9*	C	T	0.78	-0.079	0.007	2.3 E-28	110347	0.22	0.00115291	127.217045
18	*rs2078267**	11	*SLC22A11*	C	T	0.49	-0.073	0.006	9.4 E-38	110347	0.49	0.00133968	147.825449
19	*rs478607**	11	*NRXN2*	G	A	0.16	-0.047	0.007	4.4 E-11	110347	0.16	0.00040838	45.0619972
20	*rs3741414**	12	*INHBC*	C	T	0.76	-0.072	0.007	2.2 E-25	110347	0.24	0.00095784	105.691709
21	*rs653178**	12	*ATXN2*	C	T	0.49	-0.035	0.005	7.2 E-12	110347	0.49	0.00044386	48.9769195
22	*rs1394125*	15	*UBE2Q2*	A	G	0.34	0.043	0.006	2.5 E-13	110347	0.34	0.00046523	51.3358205

23	*rs6598541*	15	*IGF1R*	A	G	0.36	0.043	0.006	4.8 E-15	110347	0.36	0.00046523	51.3358205
24	*rs7188445**	16	*MAF*	G	A	0.67	-0.032	0.005	1.6 E-9	110347	0.33	0.00037105	40.9436884
25	*rs7193778**	16	*NFAT5*	C	T	0.14	-0.046	0.008	8.2 E-10	110347	0.14	0.00029953	33.0516981
26	*rs7224610**	17	*HLF*	C	A	0.42	-0.042	0.005	5.4 E-17	110347	0.42	0.00063903	70.512993

SNP: SNP id number; Chr: chromosome; EA: effect allele; OA: other allele; EAF: effect allele frequency; G: genetic variant or SNP, X: exposure (serum urate in this case); GX: beta for the variant/SNP-urate association in $mgdL^{-1}$; SE-GX: standard error of GX; *p*: p-value of GX; N: sample size of the Köttgen et al. (2013) study [3]. MAF: minor allele frequency; R2: % of variance in serum urate levels explained by each SNP; F-statistic: a measure of an instrument's strength. R2 and F-statistics were calculated by methods applied by [4] and [5], respectively.
*The direction of EA was corrected for the urate raising allele prior to analyses.

Table S5: List of 7 SNPs exclusively associated with serum urate/gout from Köttgen et al. [3] after removing pleiotropic SNPs

	SNP	Chr	Closest gene	EA	OA	EAF	GX	SE-GX	*p*	N	MAF	R2	F-statistics
1	*rs1471633*	1	*PDZK1*	A	C	0.46	0.059	0.005	1.2 E-29	110347	0.46	0.00126025	139.060742
2	*rs12498742*	4	*SLC2A9*	A	G	0.77	0.373	0.006	< 1 E-700	110347	0.23	0.03383799	3733.81943
3	*rs2231142*	4	*ABCG2*	T	G	0.11	0.217	0.009	1.0 E-134	110347	0.11	0.00524073	578.28328
4	*rs17632159**	5	*TMEM171*	G	C	0.69	-0.039	0.006	3.5 E-11	110347	0.31	0.00038274	42.2326812
5	*rs2941484*	8	*HNF4G*	T	C	0.44	0.044	0.005	4.4 E-17	110347	0.44	0.00070129	77.3835879
6	*rs2078267**	11	*SLC22A11*	C	T	0.49	-0.073	0.006	9.4 E-38	110347	0.49	0.00133968	147.825449
7	*rs7224610**	17	*HLF*	C	A	0.42	-0.042	0.005	5.4 E-17	110347	0.42	0.00063903	70.512993

SNP: SNP number; Chr: chromosome; EA: effect allele; OA: other allele; EAF: effect allele frequency; G: genetic variant or SNP, X: exposure (serum urate in this case); GX: beta for the SNP-urate association in $mgdL^{-1}$; SE-GX: standard error of GX; p: p-value of GX; N: sample size of the Köttgen et al. (2013) study [3]. MAF: minor allele frequency; R2: % of variance in serum urate levels explained by each SNP; F-statistic: a measure of an instrument's strength. R2 and F-statistics were calculated by methods applied by [4] and [5], respectively.
Note: The pleiotropic SNPs were excluded using the information at Phenoscanner [6].

Table S6: List of 26 SNPs showing SNP-urate association results in MDCS-MPP cohort

	SNPs	Chr	Closest gene	EA	OA	EAF	GX^	SE-GX	*p*	N#
1	*rs11264341**	1	*TRIM46*	C	T	0.57	-0.026	0.013	5.51E-02	11938
2	*rs1471633*	1	*PDZK1*	A	C	0.49	0.077	0.021	2.34E-04	11938
3	*rs1260326*	2	*GCKR*	T	C	0.37	0.047	0.014	6.19E-04	11938
4	*rs17050272*	2	*INHBB*	A	G	0.43	0.044	0.014	1.30E-03	11938
5	*rs6770152**	3	*SFMBT1*	G	T	0.41	-0.046	0.014	8.11E-04	11938
6	*rs12498742*	4	*SLC2A9*	A	G	0.77	0.297	0.016	4.09E-78	11938
7	*rs2231142*	4	*ABCG2*	T	G	0.09	0.219	0.022	7.35E-23	11938
8	*rs17632159**	5	*TMEM171*	G	C	0.71	-0.047	0.015	2.17E-03	11938
9	*rs1165151**	6	*SLC17A1*	G	T	0.53	-0.074	0.013	2.74E-08	11938
10	*rs675209*	6	*RREB1*	T	C	0.26	0.036	0.015	1.71E-02	11938
11	*rs729761**	6	*VEGFA*	G	T	0.72	-0.013	0.015	3.81E-01	11938
12	*rs10480300*	7	*PRKAG2*	T	C	0.26	0.012	0.015	4.19E-01	11938
13	*rs1178977*	7	*BAZ1B*	A	G	0.81	0.023	0.017	1.71E-01	11938
14	*rs17786744**	8	*STC1*	G	A	0.45	-0.009	0.013	4.68E-01	11938
15	*rs2941484*	8	*HNF4G*	T	C	0.45	0.052	0.013	1.02E-04	11938
16	*rs10821905*	10	*A1CF*	A	G	0.18	0.068	0.017	8.31E-05	11938
17	*rs1171614**	10	*SLC16A9*	C	T	0.78	-0.065	0.016	4.71E-05	11938
18	*rs2078267**	11	*SLC22A11*	C	T	0.49	-0.067	0.013	6.39E-07	11938
19	*rs478607**	11	*NRXN2*	G	A	0.17	-0.069	0.018	1.17E-04	11938
20	*rs3741414**	12	*INHBC*	C	T	0.74	-0.093	0.015	6.80E-10	11938
21	*rs653178**	12	*ATXN2*	C	T	0.49	-0.037	0.013	5.27E-03	11938
22	*rs1394125*	15	*UBE2Q2*	A	G	0.32	0.068	0.014	2.43E-06	11938
23	*rs6598541*	15	*IGF1R*	A	G	0.36	0.042	0.014	2.27E-03	11938
24	*rs7188445**	16	*MAF*	G	A	0.65	-0.017	0.014	2.33E-01	11938
25	*rs7193778**	16	*NFAT5*	C	T	0.15	-0.001	0.019	9.61E-01	11938
26	*rs7224610**	17	*HLF*	C	A	0.4	-0.029	0.014	3.34E-02	11938

MDCS-MPP: Malmö Diet Cancer Study and Malmö Preventive Project; SNP: SNP number; Chr: chromosome; EA: effect allele; OA: other allele; EAF: effect allele frequency; G: genetic variant or SNP, X: exposure (serum urate in this case); GX: beta for the SNP-urate association in $mgdL^{-1}$; SE-GX: standard error of GX; p: p-value of GX; N: total number of controls used for GX calculations in MDC-MPP cohort

^Estimates are adjusted for age and sex

#SNP-urate association analyses were done in controls only

Table S7: List of 7 SNPs associated only with serum urate/gout in [3], showing SNP-urate association in MDCS-MPP cohort

	**SNPs**	**Chr**	**Closest gene**	**EA**	**OA**	**EAF**	**GX^**	**SE-GX**	***p***	**N#**
1	*rs1471633*	1	*PDZK1*	A	C	0.49	0.077	0.021	2.34E-04	11938
2	*rs12498742*	4	*SLC2A9*	A	G	0.77	0.297	0.016	4.09E-78	11938
3	*rs2231142*	4	*ABCG2*	T	G	0.09	0.219	0.022	7.35E-23	11938
4	*rs17632159**	5	*TMEM171*	G	C	0.71	-0.047	0.015	2.17E-03	11938
5	*rs2941484*	8	*HNF4G*	T	C	0.45	0.052	0.013	1.02E-04	11938
6	*rs2078267**	11	*SLC22A11*	C	T	0.49	-0.067	0.013	6.39E-07	11938
7	*rs7224610**	17	*HLF*	C	A	0.4	-0.029	0.014	3.34E-02	11938

MDCS-MPP: Malmö Diet Cancer Study and Malmö Preventive Project; SNP: SNP number; Chr: chromosome; EA: effect allele; OA: other allele; EAF: effect allele frequency; G: genetic variant or SNP, X: exposure (serum urate in this case); GX: beta for the SNP-urate association in mgdL^{-1}; SE-GX: standard error of GX; p: p-value of GX; N: total number of controls used for GX calculations in MDC-MPP cohort

^Estimates are adjusted for age and sex

#SNP-urate association analyses were done in controls only

Table S8: The association estimates of the 26 SNPs for urate with bladder cancer in the MDCS-MPP cohort

	**SNPs**	**Chr**	**EA**	**OA**	**GY'**	**SE-GY**	***p***
1	*rs11264341**	1	C	T	-0.02305198	0.064210493	0.71959028
2	*rs1471633*	1	A	C	-0.078263057	0.098291456	0.4258957
3	*rs1260326*	2	T	C	0.044831169	0.065607922	0.49440501
4	*rs17050272*	2	A	G	0.064238688	0.064172188	0.31680927
5	*rs6770152**	3	G	T	0.047540121	0.065280459	0.46646406
6	*rs12498742*	4	A	G	-0.047748339	0.075420115	0.52666952
7	*rs2231142*	4	T	G	0.001915166	0.107030172	0.98572365
8	*rs17632159**	5	G	C	8.72E-05	0.07379044	0.9990571
9	*rs1165151**	6	G	T	-0.025805961	0.063734082	0.68555066
10	*rs675209*	6	T	C	-0.022577032	0.072612367	0.75585731
11	*rs729761**	6	G	T	-0.161575444	0.074295355	0.02964722
12	*rs10480300*	7	T	C	0.058083721	0.072111338	0.4205469
13	*rs1178977*	7	A	G	0.080149586	0.080961175	0.32218606
14	*rs17786744**	8	G	A	0.061448671	0.064145892	0.33808708
15	*rs2941484*	8	T	C	0.06435361	0.063914677	0.31399846
16	*rs10821905*	10	A	G	-0.090582768	0.084865448	0.28580514
17	*rs1171614**	10	C	T	0.01006516	0.076733061	0.89563995
18	*rs2078267**	11	C	T	0.064311557	0.06430449	0.31725733
19	*rs478607**	11	G	A	-0.092688774	0.083895838	0.26924289
20	*rs3741414**	12	C	T	0.076578281	0.071226271	0.28231163
21	*rs653178**	12	C	T	-0.103307557	0.063597317	0.10429022
22	*rs1394125*	15	A	G	0.129435658	0.067422869	0.05488835
23	*rs6598541*	15	A	G	0.030837273	0.065971348	0.64018917
24	*rs7188445**	16	G	A	-0.017123031	0.067541388	0.79986712
25	*rs7193778**	16	C	T	-0.05743491	0.089029467	0.51884782
26	*rs7224610**	17	C	A	0.029193889	0.065328924	0.65496502

MDCS-MPP: Malmö Diet Cancer Study and Malmö Preventive Project, SNP: SNP number; Chr: chromosome; EA: effect allele; OA: other allele; G: genetic variant or SNP, Y: outcome (cancer in this case); GY: beta for the SNP-cancer association; SE-GY: standard error of GY; p: p-value of GY.
'Estimates are adjusted for age and sex.
*The direction of EA was corrected for the urate raising allele prior to analyses.
SNP-cancer association analyses were done after excluding controls.

Table S9: The association estimates of the 26 SNPs for urate with colorectal cancer in the MDCS-MPP cohort

	SNP	Chr	EA	OA	GY'	SE-GY	*p*
1	*rs11264341**	1	C	T	-0.032808488	0.052082389	0.52873751
2	*rs1471633*	1	A	C	-0.060711117	0.081821775	0.45809208
3	*rs1260326*	2	T	C	0.008846459	0.053188154	0.86790209
4	*rs17050272*	2	A	G	-0.019941477	0.052344096	0.70322626
5	*rs6770152**	3	G	T	0.006181506	0.052604039	0.90645583
6	*rs12498742*	4	A	G	0.026811748	0.062171002	0.66628071
7	*rs2231142*	4	T	G	-0.059911209	0.088186265	0.49690269
8	*rs17632159**	5	G	C	0.063039479	0.059081904	0.28597881
9	*rs1165151**	6	G	T	-0.041308053	0.051595979	0.42335974
10	*rs675209*	6	T	C	-0.107988067	0.060068125	0.07221506
11	*rs729761**	6	G	T	0.033282834	0.057381509	0.5618963
12	*rs10480300*	7	T	C	-0.007338988	0.058939467	0.90090564
13	*rs1178977*	7	A	G	0.06039429	0.065152605	0.35394386
14	*rs17786744**	8	G	A	0.036468455	0.051806712	0.48147448
15	*rs2941484*	8	T	C	0.038178296	0.05190429	0.46200348
16	*rs10821905*	10	A	G	-0.13494771	0.069808444	0.0532221
17	*rs1171614**	10	C	T	-0.010592431	0.062238343	0.86485962
18	*rs2078267**	11	C	T	0.016614607	0.051782535	0.74832142
19	*rs478607**	11	G	A	-0.083177759	0.068125173	0.22210312
20	*rs3741414**	12	C	T	0.042341794	0.057965643	0.46510683
21	*rs653178**	12	C	T	0.024198901	0.05138335	0.63767766
22	*rs1394125*	15	A	G	-0.074324044	0.056221516	0.18617289
23	*rs6598541*	15	A	G	0.137334246	0.052642187	0.00908537
24	*rs7188445**	16	G	A	-0.066434091	0.054833425	0.2256801
25	*rs7193778**	16	C	T	-0.033118736	0.072424146	0.64746363
26	*rs7224610**	17	C	A	-0.008176239	0.052560129	0.87638008

MDCS-MPP: Malmö Diet Cancer Study and Malmö Preventive Project, SNP: SNP number; Chr: chromosome; EA: effect allele; OA: other allele; G: genetic variant or SNP, Y: outcome (cancer in this case); GY: beta for the SNP-cancer association; SE-GY: standard error of GY; p: p-value of GY.
'Estimates are adjusted for age and sex.
*The direction of EA was corrected for the urate raising allele prior to analyses.
SNP-cancer association analyses were done after excluding controls.

Table S10: The association estimates of the 26 SNPs for urate with brain tumor in the MDCS-MPP cohort

	SNP	Chr	EA	OA	GY'	SE-GY	*p*
1	*rs11264341**	1	C	T	0.053631885	0.132564788	0.68579279
2	*rs1471633*	1	A	C	-0.112060176	0.209527793	0.59277261
3	*rs1260326*	2	T	C	-0.011391387	0.13648614	0.9334843
4	*rs17050272*	2	A	G	-0.05640391	0.134566728	0.67510506
5	*rs6770152**	3	G	T	0.309332191	0.140016482	0.02715687
6	*rs12498742*	4	A	G	-0.162749864	0.151545213	0.28285135
7	*rs2231142*	4	T	G	-0.6085781	0.28623843	0.03349302
8	*rs17632159**	5	G	C	0.147258556	0.148484402	0.32132228
9	*rs1165151**	6	G	T	-0.098961815	0.132539434	0.45526918
10	*rs675209*	6	T	C	-0.028697351	0.151152032	0.84942078
11	*rs729761**	6	G	T	0.023518676	0.146920655	0.87282011
12	*rs10480300*	7	T	C	0.107934099	0.147147148	0.46324662
13	*rs1178977*	7	A	G	-0.122412912	0.158239666	0.43917221
14	*rs17786744**	8	G	A	-0.007685559	0.132198626	0.95363994
15	*rs2941484*	8	T	C	0.043857978	0.133265314	0.74207805
16	*rs10821905*	10	A	G	0.051395711	0.16905754	0.76111789
17	*rs1171614**	10	C	T	0.116941335	0.153702725	0.44675981
18	*rs2078267**	11	C	T	0.372791762	0.135852167	0.00606773
19	*rs478607**	11	G	A	-0.161353535	0.169544902	0.34125618
20	*rs3741414**	12	C	T	-0.050114985	0.15105744	0.74006965
21	*rs653178**	12	C	T	0.107830034	0.131867531	0.41352005
22	*rs1394125*	15	A	G	0.184748135	0.138054251	0.18082196
23	*rs6598541*	15	A	G	-0.052055795	0.137714288	0.7054317
24	*rs7188445**	16	G	A	-0.255127397	0.145292636	0.07909601
25	*rs7193778**	16	C	T	-0.528057457	0.159472686	0.00092873
26	*rs7224610**	17	C	A	-0.192433731	0.132040096	0.14500914

MDCS-MPP: Malmö Diet Cancer Study and Malmö Preventive Project, SNP: SNP number; Chr: chromosome; EA: effect allele; OA: other allele; G: genetic variant or SNP, Y: outcome (cancer in this case); GY: beta for the SNP-cancer association; SE-GY: standard error of GY; p: p-value of GY.
'Estimates are adjusted for age and sex.
*The direction of EA was corrected for the urate raising allele prior to analyses.
SNP-cancer association analyses were done after excluding controls.

Table S11: The association estimates of the 26 SNPs for urate with breast cancer in the MDCS-MPP cohort

	SNP	Chr	EA	OA	GY'	SE-GY	*p*
1	*rs11264341**	1	C	T	-0.008703187	0.054143864	0.87229669
2	*rs1471633*	1	A	C	0.038890983	0.092256814	0.67335253
3	*rs1260326*	2	T	C	0.047005088	0.054837512	0.39135076
4	*rs17050272*	2	A	G	0.046888391	0.054342569	0.38823103
5	*rs6770152**	3	G	T	0.022217522	0.054590517	0.684019
6	*rs12498742*	4	A	G	-0.001799215	0.06404885	0.97758934
7	*rs2231142*	4	T	G	-0.115803071	0.090933526	0.20284369
8	*rs17632159**	5	G	C	-0.020176284	0.062540395	0.74698907
9	*rs1165151**	6	G	T	0.038147358	0.054055373	0.48036919
10	*rs675209*	6	T	C	0.11614144	0.060011582	0.0529514
11	*rs729761**	6	G	T	-0.059539637	0.060834947	0.32772438
12	*rs10480300*	7	T	C	0.022311649	0.060724321	0.71330184
13	*rs1178977*	7	A	G	0.071820201	0.068001261	0.29089519
14	*rs17786744**	8	G	A	0.065254136	0.053531414	0.22284885
15	*rs2941484*	8	T	C	-0.035984139	0.054895174	0.51214175
16	*rs10821905*	10	A	G	0.031760984	0.069845952	0.64930417
17	*rs1171614**	10	C	T	-0.048584697	0.065669924	0.45940207
18	*rs2078267**	11	C	T	0.0146452	0.054010718	0.78627291
19	*rs478607**	11	G	A	-0.023259157	0.072501707	0.74835559
20	*rs3741414**	12	C	T	0.02226296	0.060151859	0.71129861
21	*rs653178**	12	C	T	0.185228205	0.054056331	0.00061124
22	*rs1394125*	15	A	G	0.045824248	0.057209424	0.42313671
23	*rs6598541*	15	A	G	0.08190445	0.054770039	0.13480373
24	*rs7188445**	16	G	A	-0.095872057	0.057120516	0.09326568
25	*rs7193778**	16	C	T	-0.125014753	0.072749075	0.08571681
26	*rs7224610**	17	C	A	-0.023305884	0.054138999	0.66684479

MDCS-MPP: Malmö Diet Cancer Study and Malmö Preventive Project, SNP: SNP number; Chr: chromosome; EA: effect allele; OA: other allele; G: genetic variant or SNP, Y: outcome (cancer in this case); GY: beta for the SNP-cancer association; SE-GY: standard error of GY; p: p-value of GY.
'Estimates are adjusted for age.
*The direction of EA was corrected for the urate raising allele prior to analyses.
SNP-cancer association analyses were done after excluding controls.

Table S12: The association estimates of the 26 SNPs for urate with gynecological cancer in the MDCS-MPP cohort

	SNP	Chr	EA	OA	GY'	SE-GY	*p*
1	*rs11264341**	1	C	T	0.071536831	0.053180131	0.17856722
2	*rs1471633*	1	A	C	0.013392602	0.090904533	0.8828747
3	*rs1260326*	2	T	C	-0.013224736	0.054682873	0.80890088
4	*rs17050272*	2	A	G	0.074157392	0.0538044	0.16811764
5	*rs6770152**	3	G	T	-0.007153032	0.053695348	0.89402326
6	*rs12498742*	4	A	G	0.089806688	0.064424119	0.16332011
7	*rs2231142*	4	T	G	0.028107021	0.084720717	0.74006981
8	*rs17632159**	5	G	C	-0.045412537	0.061816303	0.46256066
9	*rs1165151**	6	G	T	0.068843944	0.052972025	0.19372839
10	*rs675209*	6	T	C	-0.042767601	0.060748333	0.48142483
11	*rs729761**	6	G	T	0.051004963	0.058813195	0.38581231
12	*rs10480300*	7	T	C	-0.099115698	0.061449824	0.10675465
13	*rs1178977*	7	A	G	0.063611564	0.066247568	0.33694964
14	*rs17786744**	8	G	A	-0.013854626	0.052664978	0.79249589
15	*rs2941484*	8	T	C	0.01735908	0.053869332	0.74726769
16	*rs10821905*	10	A	G	-0.003800945	0.069195257	0.95619366
17	*rs1171614**	10	C	T	0.039018785	0.06280542	0.53442554
18	*rs2078267**	11	C	T	-0.053019108	0.052942873	0.31661415
19	*rs478607**	11	G	A	-0.062936485	0.070412627	0.37141616
20	*rs3741414**	12	C	T	0.028370532	0.058664853	0.62866724
21	*rs653178**	12	C	T	0.084721293	0.052970362	0.10972967
22	*rs1394125*	15	A	G	0.121362254	0.055716933	0.02939164
23	*rs6598541*	15	A	G	-0.009860824	0.054566641	0.85659396
24	*rs7188445**	16	G	A	-0.027390032	0.055472101	0.62147407
25	*rs7193778**	16	C	T	0.141930952	0.07747076	0.06694273
26	*rs7224610**	17	C	A	-0.049540112	0.053371977	0.35330148

MDCS-MPP: Malmö Diet Cancer Study and Malmö Preventive Project, SNP: SNP number; Chr: chromosome; EA: effect allele; OA: other allele; G: genetic variant or SNP, Y: outcome (cancer in this case); GY: beta for the SNP-cancer association; SE-GY: standard error of GY; p: p-value of GY.
'Estimates are adjusted for age.
*The direction of EA was corrected for the urate raising allele prior to analyses.
SNP-cancer association analyses were done after excluding controls.

Table S13: The association estimates of the 26 SNPs for urate with gastric cancer in the MDCS-MPP cohort

	SNP	Chr	EA	OA	GY'	SE-GY	*p*
1	*rs11264341**	1	C	T	-0.075712155	0.10867871	0.48601534
2	*rs1471633*	1	A	C	-0.108622148	0.166233822	0.51347918
3	*rs1260326*	2	T	C	-0.216583162	0.115352746	0.06043965
4	*rs17050272*	2	A	G	0.001429338	0.108841481	0.98952225
5	*rs6770152**	3	G	T	-0.044145643	0.109370749	0.68648262
6	*rs12498742*	4	A	G	0.105979864	0.132741691	0.42464322
7	*rs2231142*	4	T	G	-0.206913097	0.196867285	0.29324557
8	*rs17632159**	5	G	C	-0.141485842	0.128245969	0.26992377
9	*rs1165151**	6	G	T	-0.163067092	0.108278233	0.132067
10	*rs675209*	6	T	C	-0.117309434	0.125529476	0.35003725
11	*rs729761**	6	G	T	-0.007237478	0.121007488	0.95230683
12	*rs10480300*	7	T	C	-0.039437125	0.124130677	0.75070757
13	*rs1178977*	7	A	G	0.11401641	0.138376198	0.40996246
14	*rs17786744**	8	G	A	0.215013561	0.109690117	0.04997349
15	*rs2941484*	8	T	C	-0.075732333	0.10853201	0.48530979
16	*rs10821905*	10	A	G	-0.071369351	0.142608841	0.61675452
17	*rs1171614**	10	C	T	-0.305973118	0.142299173	0.0315386
18	*rs2078267**	11	C	T	0.16071924	0.108934602	0.14011212
19	*rs478607**	11	G	A	-0.081142463	0.14193152	0.56752422
20	*rs3741414**	12	C	T	-0.011314266	0.122375992	0.92633658
21	*rs653178**	12	C	T	0.130469152	0.107642746	0.22549053
22	*rs1394125*	15	A	G	0.045674893	0.115144592	0.6916077
23	*rs6598541*	15	A	G	0.057702514	0.110512906	0.60157723
24	*rs7188445**	16	G	A	-0.055494343	0.114386828	0.6275726
25	*rs7193778**	16	C	T	0.184709294	0.163140969	0.25754755
26	*rs7224610**	17	C	A	0.029421133	0.110253512	0.78958477

MDCS-MPP: Malmö Diet Cancer Study and Malmö Preventive Project, SNP: SNP number; Chr: chromosome; EA: effect allele; OA: other allele; G: genetic variant or SNP, Y: outcome (cancer in this case); GY: beta for the SNP-cancer association; SE-GY: standard error of GY; p: p-value of GY.
'Estimates are adjusted for age and sex.
*The direction of EA was corrected for the urate raising allele prior to analyses.
SNP-cancer association analyses were done after excluding controls.

Table S14: The association estimates of the 26 SNPs for urate with hepatic cancer in the MDCS-MPP cohort

	SNP	Chr	EA	OA	GY'	SE-GY	*p*
1	*rs11264341**	1	C	T	-0.046535	0.20658221	0.82177584
2	*rs1471633*	1	A	C	0.76619103	0.30315781	0.01149214
3	*rs1260326*	2	T	C	-0.2301314	0.21754474	0.29012022
4	*rs17050272*	2	A	G	0.08162295	0.20531756	0.69096569
5	*rs6770152**	3	G	T	-0.1032964	0.20688653	0.61757508
6	*rs12498742*	4	A	G	0.36370341	0.26926032	0.17677548
7	*rs2231142*	4	T	G	-0.1501323	0.36613675	0.68177349
8	*rs17632159**	5	G	C	0.01807345	0.23576368	0.93889468
9	*rs1165151**	6	G	T	0.09233269	0.20183287	0.64733251
10	*rs675209*	6	T	C	0.12363493	0.22293978	0.57919123
11	*rs729761**	6	G	T	0.12700202	0.22068507	0.56496005
12	*rs10480300*	7	T	C	0.3932494	0.21756362	0.0706821
13	*rs1178977*	7	A	G	0.33337582	0.28029167	0.23428664
14	*rs17786744**	8	G	A	-0.1912419	0.20289348	0.3458993
15	*rs2941484*	8	T	C	-0.0060435	0.20586204	0.97658002
16	*rs10821905*	10	A	G	0.29219939	0.24474836	0.23252617
17	*rs1171614**	10	C	T	0.24951189	0.22848805	0.27482744
18	*rs2078267**	11	C	T	0.04378918	0.20578853	0.83149289
19	*rs478607**	11	G	A	0.52339901	0.33533811	0.11856864
20	*rs3741414**	12	C	T	-0.5999548	0.27521558	0.02926155
21	*rs653178**	12	C	T	0.16932221	0.2049779	0.40877514
22	*rs1394125*	15	A	G	0.20960183	0.21325851	0.32567965
23	*rs6598541*	15	A	G	-0.0916216	0.21295117	0.667016
24	*rs7188445**	16	G	A	-0.1907715	0.22311903	0.39253954
25	*rs7193778**	16	C	T	-0.3768362	0.25816239	0.14437616
26	*rs7224610**	17	C	A	-0.2161677	0.20551627	0.29287853

MDCS-MPP: Malmö Diet Cancer Study and Malmö Preventive Project, SNP: SNP number; Chr: chromosome; EA: effect allele; OA: other allele; G: genetic variant or SNP, Y: outcome (cancer in this case); GY: beta for the SNP-cancer association; SE-GY: standard error of GY; p: p-value of GY.
'Estimates are adjusted for age and sex.
*The direction of EA was corrected for the urate raising allele prior to analyses.
SNP-cancer association analyses were done after excluding controls.

Table S15: The association estimates of the 26 SNPs for urate with skin cancer in the MDCS-MPP cohort

	SNP	Chr	EA	OA	GY'	SE-GY	*p*
1	*rs11264341**	1	C	T	-0.005607639	0.073082706	0.93883831
2	*rs1471633*	1	A	C	0.048065763	0.114157396	0.67371991
3	*rs1260326*	2	T	C	0.010644723	0.074777289	0.8868017
4	*rs17050272*	2	A	G	0.046741611	0.073197568	0.52310413
5	*rs6770152**	3	G	T	0.052953676	0.074230822	0.47561988
6	*rs12498742*	4	A	G	-0.106496546	0.084507187	0.20759471
7	*rs2231142*	4	T	G	-0.07809107	0.124767456	0.53138516
8	*rs17632159**	5	G	C	-0.100967642	0.085711621	0.23879962
9	*rs1165151**	6	G	T	-0.138987597	0.07293784	0.05670702
10	*rs675209*	6	T	C	-0.076825701	0.083976422	0.36027119
11	*rs729761**	6	G	T	-0.104832245	0.083330578	0.20838144
12	*rs10480300*	7	T	C	-0.018713816	0.083118767	0.82186609
13	*rs1178977*	7	A	G	0.076693799	0.092095911	0.40498005
14	*rs17786744**	8	G	A	0.04165706	0.072990522	0.5681903
15	*rs2941484*	8	T	C	-0.0015852	0.073069195	0.98269165
16	*rs10821905*	10	A	G	0.109184668	0.091372031	0.23210802
17	*rs1171614**	10	C	T	-0.074582076	0.089147763	0.40281078
18	*rs2078267**	11	C	T	-0.075826433	0.072805688	0.29764796
19	*rs478607**	11	G	A	-0.054119759	0.09643381	0.57465339
20	*rs3741414**	12	C	T	0.031305062	0.081639722	0.70138331
21	*rs653178**	12	C	T	0.022977393	0.072244054	0.7504452
22	*rs1394125*	15	A	G	-0.01391127	0.078222116	0.85884613
23	*rs6598541*	15	A	G	-0.037382727	0.07572252	0.62153188
24	*rs7188445**	16	G	A	-0.068342241	0.077276088	0.37648543
25	*rs7193778**	16	C	T	0.181672516	0.109884213	0.09826807
26	*rs7224610**	17	C	A	-0.123089096	0.073105938	0.092238

MDCS-MPP: Malmö Diet Cancer Study and Malmö Preventive Project, SNP: SNP number; Chr: chromosome; EA: effect allele; OA: other allele; G: genetic variant or SNP, Y: outcome (cancer in this case); GY: beta for the SNP-cancer association; SE-GY: standard error of GY; p: p-value of GY.
'Estimates are adjusted for age and sex.
*The direction of EA was corrected for the urate raising allele prior to analyses.
SNP-cancer association analyses were done after excluding controls.

Table S16: The association estimates of the 26 SNPs for urate with renal cancer in the MDCS-MPP cohort

	SNP	Chr	EA	OA	GY'	SE-GY	*p*
1	*rs11264341**	1	C	T	0.135616377	0.106349389	0.20223967
2	*rs1471633*	1	A	C	-0.044169984	0.164953911	0.78887508
3	*rs1260326*	2	T	C	0.017669018	0.109331303	0.87161308
4	*rs17050272*	2	A	G	0.056490337	0.107033005	0.59764907
5	*rs6770152**	3	G	T	-0.09073699	0.107548163	0.39884432
6	*rs12498742*	4	A	G	-0.073981722	0.124546954	0.55250765
7	*rs2231142*	4	T	G	0.066090495	0.172779293	0.70208016
8	*rs17632159**	5	G	C	0.066328288	0.121470966	0.58503685
9	*rs1165151**	6	G	T	0.053843732	0.10580758	0.61083388
10	*rs675209*	6	T	C	-0.046787169	0.121677711	0.70059517
11	*rs729761**	6	G	T	-0.044716078	0.119991055	0.7094002
12	*rs10480300*	7	T	C	0.144852226	0.117808599	0.21886355
13	*rs1178977*	7	A	G	-0.219248653	0.123963767	0.07695212
14	*rs17786744**	8	G	A	-0.065869344	0.106126089	0.53481637
15	*rs2941484*	8	T	C	-0.027578097	0.1072227	0.79702137
16	*rs10821905*	10	A	G	-0.026767914	0.139163145	0.84746871
17	*rs1171614**	10	C	T	0.011289022	0.127164246	0.92926063
18	*rs2078267**	11	C	T	0.00417322	0.106725765	0.96880885
19	*rs478607**	11	G	A	-0.157926127	0.136634448	0.2477509
20	*rs3741414**	12	C	T	0.137534574	0.117024644	0.23989006
21	*rs653178**	12	C	T	0.015206332	0.105815185	0.88573222
22	*rs1394125*	15	A	G	0.176298455	0.111591264	0.11413918
23	*rs6598541*	15	A	G	-0.10026005	0.111561501	0.36881377
24	*rs7188445**	16	G	A	0.046277027	0.111453841	0.67798635
25	*rs7193778**	16	C	T	0.10349924	0.156869716	0.50939609
26	*rs7224610**	17	C	A	0.064526708	0.109161665	0.55444576

MDCS-MPP: Malmö Diet Cancer Study and Malmö Preventive Project, SNP: SNP number; Chr: chromosome; EA: effect allele; OA: other allele; G: genetic variant or SNP, Y: outcome (cancer in this case); GY: beta for the SNP-cancer association; SE-GY: standard error of GY; p: p-value of GY.
'Estimates are adjusted for age and sex.
*The direction of EA was corrected for the urate raising allele prior to analyses.
SNP-cancer association analyses were done after excluding controls.

Table S17: The association estimates of the 26 SNPs for urate with lung cancer in the MDCS-MPP cohort

	SNP	Chr	EA	OA	GY'	SE-GY	*p*
1	*rs11264341**	1	C	T	0.050501998	0.062080737	0.41593774
2	*rs1471633*	1	A	C	0.033477736	0.09739797	0.73105605
3	*rs1260326*	2	T	C	0.098133561	0.063249919	0.12077694
4	*rs17050272*	2	A	G	-0.043158041	0.062990883	0.4932509
5	*rs6770152**	3	G	T	-0.110038869	0.062654648	0.07904125
6	*rs12498742*	4	A	G	-0.005605276	0.074065981	0.93967409
7	*rs2231142*	4	T	G	0.150691254	0.097278451	0.12136459
8	*rs17632159**	5	G	C	0.003814736	0.071766639	0.95760863
9	*rs1165151**	6	G	T	-0.025941624	0.061938658	0.67534208
10	*rs675209*	6	T	C	0.028879783	0.069873047	0.67937369
11	*rs729761**	6	G	T	0.014848277	0.069117255	0.82990192
12	*rs10480300*	7	T	C	0.003365161	0.070716771	0.96204582
13	*rs1178977*	7	A	G	0.047576514	0.077927146	0.54151372
14	*rs17786744**	8	G	A	0.008590634	0.062093734	0.88996425
15	*rs2941484*	8	T	C	0.027561878	0.06247633	0.65909925
16	*rs10821905*	10	A	G	0.015563626	0.080113234	0.8459642
17	*rs1171614**	10	C	T	0.093341663	0.07274814	0.19946415
18	*rs2078267**	11	C	T	0.001612467	0.062266906	0.97934025
19	*rs478607**	11	G	A	-0.164279759	0.079615049	0.03907218
20	*rs3741414**	12	C	T	0.005914721	0.070074273	0.93273327
21	*rs653178**	12	C	T	-0.045138018	0.061617718	0.46383334
22	*rs1394125*	15	A	G	0.07343529	0.06573368	0.26392428
23	*rs6598541*	15	A	G	0.019461755	0.064018344	0.76112576
24	*rs7188445**	16	G	A	0.018994608	0.065021821	0.77018977
25	*rs7193778**	16	C	T	0.048511271	0.089549956	0.58800934
26	*rs7224610**	17	C	A	-0.025282506	0.062797705	0.68724075

MDCS-MPP: Malmö Diet Cancer Study and Malmö Preventive Project, SNP: SNP number; Chr: chromosome; EA: effect allele; OA: other allele; G: genetic variant or SNP, Y: outcome (cancer in this case); GY: beta for the SNP-cancer association; SE-GY: standard error of GY; p: p-value of GY.
'Estimates are adjusted for age and sex.
*The direction of EA was corrected for the urate raising allele prior to analyses.
SNP-cancer association analyses were done after excluding controls.

Table S18: The association estimates of the 26 SNPs for urate with lymphatic and hematopoietic cancer in the MDCS-MPP cohort

	SNP	Chr	EA	OA	GY'	SE-GY	*p*
1	*rs11264341**	1	C	T	-0.066415854	0.068082629	0.3293032
2	*rs1471633*	1	A	C	-0.108799498	0.106667584	0.30773485
3	*rs1260326*	2	T	C	0.011434261	0.069369012	0.8690759
4	*rs17050272*	2	A	G	0.054693445	0.067938705	0.42079601
5	*rs6770152**	3	G	T	-0.000273199	0.068532429	0.99681931
6	*rs12498742*	4	A	G	0.042209796	0.081276686	0.60352742
7	*rs2231142*	4	T	G	-0.122400439	0.117474471	0.29744309
8	*rs17632159**	5	G	C	-0.076397484	0.079118941	0.33424289
9	*rs1165151**	6	G	T	0.02342256	0.06716101	0.72727524
10	*rs675209*	6	T	C	0.007399248	0.076284626	0.92273015
11	*rs729761**	6	G	T	0.137848708	0.073220734	0.05974838
12	*rs10480300*	7	T	C	0.03509979	0.076084402	0.64456401
13	*rs1178977*	7	A	G	0.0510772	0.084666756	0.54632684
14	*rs17786744**	8	G	A	-0.086802682	0.067129239	0.19598748
15	*rs2941484*	8	T	C	0.016050338	0.067803902	0.81287633
16	*rs10821905*	10	A	G	0.116401335	0.084569863	0.16870012
17	*rs1171614**	10	C	T	0.003588195	0.08072821	0.96454742
18	*rs2078267**	11	C	T	0.020244767	0.06762779	0.76466863
19	*rs478607**	11	G	A	0.002496336	0.091200373	0.97816304
20	*rs3741414**	12	C	T	-0.078043849	0.077646946	0.3148431
21	*rs653178**	12	C	T	0.065133207	0.06701413	0.33108416
22	*rs1394125*	15	A	G	0.132886738	0.070987651	0.06121078
23	*rs6598541*	15	A	G	0.074477578	0.068918851	0.27985011
24	*rs7188445**	16	G	A	-0.047303678	0.071401912	0.50765164
25	*rs7193778**	16	C	T	-0.060063691	0.093919278	0.5224817
26	*rs7224610**	17	C	A	-0.049136134	0.06813649	0.4708217

MDCS-MPP: Malmö Diet Cancer Study and Malmö Preventive Project, SNP: SNP number; Chr: chromosome; EA: effect allele; OA: other allele; G: genetic variant or SNP, Y: outcome (cancer in this case); GY: beta for the SNP-cancer association; SE-GY: standard error of GY; p: p-value of GY.
'Estimates are adjusted for age and sex.
*The direction of EA was corrected for the urate raising allele prior to analyses.
SNP-cancer association analyses were done after excluding controls.

Table S19: The association estimates of the 26 SNPs for urate with pancreatic cancer in the MDCS-MPP cohort

	SNP	Chr	EA	OA	GY'	SE-GY	*p*
1	*rs11264341**	1	C	T	0.159629465	0.130314343	0.22059143
2	*rs1471633*	1	A	C	0.137833187	0.203454455	0.49811123
3	*rs1260326*	2	T	C	0.234293206	0.129871166	0.07122452
4	*rs17050272*	2	A	G	-0.207287808	0.134907974	0.1244127
5	*rs6770152**	3	G	T	-0.135233521	0.13123716	0.30279813
6	*rs12498742*	4	A	G	-0.003022878	0.154034858	0.98434281
7	*rs2231142*	4	T	G	0.317539918	0.19085113	0.09615051
8	*rs17632159**	5	G	C	0.087118701	0.148321851	0.55696024
9	*rs1165151**	6	G	T	0.038881279	0.128633406	0.76245064
10	*rs675209*	6	T	C	-0.00926959	0.146553084	0.94956684
11	*rs729761**	6	G	T	0.228937285	0.137409703	0.09569498
12	*rs10480300*	7	T	C	-0.192273156	0.156020395	0.21781497
13	*rs1178977*	7	A	G	-0.083176017	0.15597211	0.59384328
14	*rs17786744**	8	G	A	-0.011742112	0.128974261	0.92745899
15	*rs2941484*	8	T	C	0.128523299	0.130956015	0.32638399
16	*rs10821905*	10	A	G	0.02550925	0.168081774	0.87937079
17	*rs1171614**	10	C	T	0.05730628	0.152500225	0.7070817
18	*rs2078267**	11	C	T	-0.086949373	0.130751257	0.50605153
19	*rs478607**	11	G	A	-0.096929468	0.170833569	0.57044841
20	*rs3741414**	12	C	T	0.213052098	0.141371464	0.13180104
21	*rs653178**	12	C	T	0.155910437	0.130536694	0.2323293
22	*rs1394125*	15	A	G	-0.055206606	0.141581849	0.69659042
23	*rs6598541*	15	A	G	0.270506522	0.129265509	0.03638106
24	*rs7188445**	16	G	A	0.058821411	0.136218227	0.66587407
25	*rs7193778**	16	C	T	-0.041597129	0.182606463	0.81980438
26	*rs7224610**	17	C	A	0.35480939	0.139732113	0.01111026

MDCS-MPP: Malmö Diet Cancer Study and Malmö Preventive Project, SNP: SNP number; Chr: chromosome; EA: effect allele; OA: other allele; G: genetic variant or SNP, Y: outcome (cancer in this case); GY: beta for the SNP-cancer association; SE-GY: standard error of GY; p: p-value of GY.
'Estimates are adjusted for age and sex.
*The direction of EA was corrected for the urate raising allele prior to analyses.
SNP-cancer association analyses were done after excluding controls.

Table S20: The association estimates of the 26 SNPs for urate with prostate cancer in the MDCS-MPP cohort

	SNP	Chr	EA	OA	GY'	SE-GY	*p*
1	*rs11264341**	1	C	T	-0.009362382	0.040384857	0.81667081
2	*rs1471633*	1	A	C	-0.032514527	0.060494198	0.59093489
3	*rs1260326*	2	T	C	0.063351804	0.041066876	0.12291579
4	*rs17050272*	2	A	G	-0.051237649	0.040466589	0.20545162
5	*rs6770152**	3	G	T	-0.030438355	0.040787341	0.45550454
6	*rs12498742*	4	A	G	0.00769286	0.047710546	0.87190418
7	*rs2231142*	4	T	G	0.049269663	0.067038746	0.46237415
8	*rs17632159**	5	G	C	-0.051040853	0.046738799	0.2748135
9	*rs1165151**	6	G	T	-0.022659898	0.03980336	0.5691552
10	*rs675209*	6	T	C	0.018196364	0.045006439	0.68598787
11	*rs729761**	6	G	T	-0.015911548	0.04493334	0.72325307
12	*rs10480300*	7	T	C	0.047691613	0.045582282	0.29543397
13	*rs1178977*	7	A	G	-0.029983113	0.049345524	0.54344241
14	*rs17786744**	8	G	A	0.011891475	0.040072795	0.76665981
15	*rs2941484*	8	T	C	-0.02825733	0.040181947	0.48190965
16	*rs10821905*	10	A	G	-0.027595717	0.052442156	0.59874058
17	*rs1171614**	10	C	T	0.008531302	0.047861365	0.85852641
18	*rs2078267**	11	C	T	-0.007078773	0.040054804	0.85972268
19	*rs478607**	11	G	A	-0.059803839	0.053337698	0.26218988
20	*rs3741414**	12	C	T	0.049443386	0.045118031	0.27313674
21	*rs653178**	12	C	T	-0.011065556	0.03983584	0.78118209
22	*rs1394125*	15	A	G	-0.027185321	0.043219601	0.52934614
23	*rs6598541*	15	A	G	0.076093937	0.041177935	0.06461249
24	*rs7188445**	16	G	A	0.024602059	0.042311296	0.56093472
25	*rs7193778**	16	C	T	-0.070858938	0.055996012	0.20571784
26	*rs7224610**	17	C	A	0.016321296	0.041113427	0.69138064

MDCS-MPP: Malmö Diet Cancer Study and Malmö Preventive Project, SNP: SNP number; Chr: chromosome; EA: effect allele; OA: other allele; G: genetic variant or SNP, Y: outcome (cancer in this case); GY: beta for the SNP-cancer association; SE-GY: standard error of GY; p: p-value of GY.
'Estimates are adjusted for age.
*The direction of EA was corrected for the urate raising allele prior to analyses.
SNP-cancer association analyses were done after excluding controls.

Table S21: The association estimates of the 26 SNPs for urate with All-cause cancer in the MDCS-MPP cohort

	SNP	Chr	EA	OA	GY'	SE-GY	*p*
1	*rs11264341**	1	C	T	0.018727249	0.022995139	0.41541564
2	*rs1471633*	1	A	C	-0.000166259	0.036575312	0.99637309
3	*rs1260326*	2	T	C	0.017541272	0.023571424	0.45677042
4	*rs17050272*	2	A	G	0.002990844	0.023099978	0.89698269
5	*rs6770152**	3	G	T	-0.015322617	0.023289147	0.51058345
6	*rs12498742*	4	A	G	0.019403262	0.027351239	0.47806965
7	*rs2231142*	4	T	G	-0.011187919	0.038332851	0.77039182
8	*rs17632159**	5	G	C	-0.015916306	0.026592432	0.54948791
9	*rs1165151**	6	G	T	-0.010397262	0.022862482	0.64927184
10	*rs675209*	6	T	C	-0.005334226	0.025985353	0.83735478
11	*rs729761**	6	G	T	-0.011823559	0.025710588	0.64560867
12	*rs10480300*	7	T	C	0.013696463	0.026066162	0.59927045
13	*rs1178977*	7	A	G	0.018367305	0.028580923	0.52045633
14	*rs17786744**	8	G	A	0.025129328	0.02278619	0.27010034
15	*rs2941484*	8	T	C	6.62E-05	0.023089444	0.9977129
16	*rs10821905*	10	A	G	-0.018135401	0.02996685	0.54505801
17	*rs1171614**	10	C	T	-0.003622469	0.027484015	0.8951403
18	*rs2078267**	11	C	T	0.009724534	0.022919376	0.67135203
19	*rs478607**	11	G	A	-0.065954822	0.030558209	0.03090187
20	*rs3741414**	12	C	T	0.028244003	0.025657465	0.27097922
21	*rs653178**	12	C	T	0.036042975	0.022774958	0.1135194
22	*rs1394125*	15	A	G	0.036921986	0.024415943	0.13048094
23	*rs6598541*	15	A	G	0.057929265	0.023656591	0.01433497
24	*rs7188445**	16	G	A	-0.019120688	0.024145153	0.42841559
25	*rs7193778**	16	C	T	-0.015614272	0.032315212	0.62896337
26	*rs7224610**	17	C	A	-0.015153139	0.023241004	0.51440105

MDCS-MPP: Malmö Diet Cancer Study and Malmö Preventive Project, SNP: SNP number; Chr: chromosome; EA: effect allele; OA: other allele; G: genetic variant or SNP, Y: outcome (cancer in this case); GY: beta for the SNP-cancer association; SE-GY: standard error of GY; p: p-value of GY.
'Estimates are adjusted for age and sex.
*The direction of EA was corrected for the urate raising allele prior to analyses.
SNP-cancer association analyses were done after excluding controls.

Table S22: The association estimates of the 26 SNPs for urate with bladder cancer in the UK-Biobank cohort

	SNP	Chr	EA	OA	EAF	GY^	SE-GY	*p*
1	*rs11264341**	1	C	T	0.57	-0.0069464	0.0304242	0.819
2	*rs1471633*	1	A	C	0.46	-0.000786	0.0301725	0.979
3	*rs1260326*	2	T	C	0.4	0.0150914	0.0307701	0.624
4	*rs17050272*	2	A	G	0.41	0.0538012	0.0305862	0.079
5	*rs6770152**	3	G	T	0.42	-0.002631	0.0318842	0.934
6	*rs12498742*	4	A	G	0.77	0.0126097	0.0356619	0.724
7	*rs2231142*	4	T	G	0.11	-0.0005207	0.0474596	0.991
8	*rs17632159**	5	G	C	0.7	-0.0335004	0.0327587	0.306
9	*rs1165151**	6	G	T	0.55	0.0360268	0.0302757	0.234
10	*rs675209*	6	T	C	0.27	0.0328896	0.0339725	0.333
11	*rs729761**	6	G	T	0.71	-0.0049399	0.0335245	0.883
12	*rs10480300*	7	T	C	0.27	0.0135422	0.0337445	0.688
13	*rs1178977*	7	A	G	0.8	-0.0081452	0.0379409	0.83
14	*rs17786744**	8	G	A	0.41	-0.0604109	0.0306639	0.049
15	*rs2941484*	8	T	C	0.45	-0.0426404	0.0304514	0.161
16	*rs10821905*	10	A	G	0.18	-0.0188391	0.0393197	0.632
17	*rs1171614**	10	C	T	0.77	0.00044745	0.0357528	0.99
18	*rs2078267**	11	C	T	0.45	-0.0389747	0.0301853	0.197
19	*rs478607**	11	G	A	0.15	0.00523169	0.0418806	0.901
20	*rs3741414**	12	C	T	0.76	-0.0071505	0.0351037	0.839
21	*rs653178**	12	C	T	0.48	-0.0181912	0.0301301	0.546
22	*rs1394125*	15	A	G	0.36	0.00303187	0.0313298	0.923
23	*rs6598541*	15	A	G	0.36	0.0655371	0.0315101	0.038
24	*rs7188445**	16	G	A	0.67	0.0291561	0.0320688	0.363
25	*rs7193778**	16	C	T	0.15	0.0305398	0.0423572	0.471
26	*rs7224610**	17	C	A	0.4	0.00252069	0.0310204	0.935

SNP: SNP number; Chr: chromosome; EA: effect allele; OA: other allele; EAF: effect allele frequency; G: genetic variant or SNP, Y: outcome (cancer in this case); GY: beta for the SNP-cancer association; SE-GY: standard error of GY; *p*: p-value of GY.
^Estimates are adjusted for age, sex and first principal component.
*The direction of EA was corrected for the urate raising allele prior to analyses.
SNP-cancer association analyses were done after excluding controls.

Table S23: The association estimates of the 26 SNPs for urate with colorectal cancer in the UK-Biobank cohort

	SNP	Chr	EA	OA	EAF	GY^	SE-GY	*p*
1	*rs11264341**	1	C	T	0.57	-0.0032903	0.0215676	0.879
2	*rs1471633*	1	A	C	0.46	-0.014605	0.0214111	0.495
3	*rs1260326*	2	T	C	0.4	0.00588882	0.0218188	0.787
4	*rs17050272*	2	A	G	0.41	-0.0460615	0.0217123	0.034
5	*rs6770152**	3	G	T	0.42	0.0979834	0.0226074	1.46E-05
6	*rs12498742*	4	A	G	0.77	-0.0349066	0.0252699	0.167
7	*rs2231142*	4	T	G	0.11	-0.045014	0.0335008	0.179
8	*rs17632159**	5	G	C	0.7	-0.0306629	0.0232246	0.187
9	*rs1165151**	6	G	T	0.55	0.036686	0.0214524	0.087
10	*rs675209*	6	T	C	0.27	-0.0048292	0.0240487	0.841
11	*rs729761**	6	G	T	0.71	-0.00663	0.0237776	0.78
12	*rs10480300*	7	T	C	0.27	0.00492727	0.0239143	0.837
13	*rs1178977*	7	A	G	0.8	-0.0615427	0.0268253	0.022
14	*rs17786744**	8	G	A	0.41	-0.015244	0.0217578	0.484
15	*rs2941484*	8	T	C	0.45	-0.0023016	0.0215914	0.915
16	*rs10821905*	10	A	G	0.18	-0.0998951	0.0278769	0.0003391
17	*rs1171614**	10	C	T	0.77	0.0179935	0.0253672	0.478
18	*rs2078267**	11	C	T	0.45	0.0123288	0.0214246	0.565
19	*rs478607**	11	G	A	0.15	0.0576904	0.0297211	0.052
20	*rs3741414**	12	C	T	0.76	0.0671294	0.0249172	0.007
21	*rs653178**	12	C	T	0.48	0.0974522	0.0213504	5.01E-06
22	*rs1394125*	15	A	G	0.36	-0.0033344	0.0222133	0.881
23	*rs6598541*	15	A	G	0.36	0.0260126	0.0223447	0.244
24	*rs7188445**	16	G	A	0.67	-0.0363965	0.0227639	0.11
25	*rs7193778**	16	C	T	0.15	-0.0394931	0.0299504	0.187
26	*rs7224610**	17	C	A	0.4	0.0213204	0.0219794	0.332

SNP: SNP number; Chr: chromosome; EA: effect allele; OA: other allele; EAF: effect allele frequency; G: genetic variant or SNP, Y: outcome (cancer in this case); GY: beta for the SNP-cancer association; SE-GY: standard error of GY; *p*: p-value of GY.
^Estimates are adjusted for age, sex and first principal component.
*The direction of EA was corrected for the urate raising allele prior to analyses.
SNP-cancer association analyses were done after excluding controls.

Table S24: The association estimates of the 26 SNPs for urate with brain tumor in the UK-Biobank cohort

	SNP	Chr	EA	OA	EAF	GY^	SE-GY	*p*
1	*rs11264341**	1	C	T	0.57	-0.031685	0.0589405	0.591
2	*rs1471633*	1	A	C	0.46	-0.0987199	0.0585786	0.092
3	*rs1260326*	2	T	C	0.4	0.0522866	0.0596886	0.381
4	*rs17050272*	2	A	G	0.41	0.0281363	0.059343	0.635
5	*rs6770152**	3	G	T	0.42	0.0524442	0.0618178	0.396
6	*rs12498742*	4	A	G	0.77	0.0632789	0.0690649	0.36
7	*rs2231142*	4	T	G	0.11	-0.0288031	0.0917087	0.753
8	*rs17632159**	5	G	C	0.7	0.00773879	0.0635712	0.903
9	*rs1165151**	6	G	T	0.55	0.0789672	0.0587243	0.179
10	*rs675209*	6	T	C	0.27	-0.0540494	0.0657056	0.411
11	*rs729761**	6	G	T	0.71	0.0233139	0.0650159	0.72
12	*rs10480300*	7	T	C	0.27	-0.0094298	0.0654164	0.885
13	*rs1178977*	7	A	G	0.8	0.0632518	0.0735156	0.39
14	*rs17786744**	8	G	A	0.41	-0.124346	0.0594643	0.037
15	*rs2941484*	8	T	C	0.45	0.0867096	0.0589942	0.142
16	*rs10821905*	10	A	G	0.18	-0.0987278	0.0764658	0.197
17	*rs1171614**	10	C	T	0.77	0.123703	0.069313	0.074
18	*rs2078267**	11	C	T	0.45	0.0883851	0.05863	0.132
19	*rs478607**	11	G	A	0.15	0.0895989	0.0811251	0.269
20	*rs3741414**	12	C	T	0.76	-0.0903626	0.0681517	0.185
21	*rs653178**	12	C	T	0.48	-0.0095555	0.0584341	0.87
22	*rs1394125*	15	A	G	0.36	-0.0596677	0.0607813	0.326
23	*rs6598541*	15	A	G	0.36	-0.0018031	0.0611253	0.976
24	*rs7188445**	16	G	A	0.67	0.0318968	0.0623063	0.609
25	*rs7193778**	16	C	T	0.15	0.0132215	0.082143	0.872
26	*rs7224610**	17	C	A	0.4	0.0288651	0.0600487	0.631

SNP: SNP number; Chr: chromosome; EA: effect allele; OA: other allele; EAF: effect allele frequency; G: genetic variant or SNP, Y: outcome (cancer in this case); GY: beta for the SNP-cancer association; SE-GY: standard error of GY; *p*: p-value of GY.
^Estimates are adjusted for age, sex and first principal component.
*The direction of EA was corrected for the urate raising allele prior to analyses.
SNP-cancer association analyses were done after excluding controls.

Table S25: The association estimates of the 26 SNPs for urate with gastric cancer in the UK-Biobank cohort

	SNP	Chr	EA	OA	EAF	GY^	SE-GY	*p*
1	*rs11264341**	1	C	T	0.57	0.0493737	0.04142	0.233
2	*rs1471633*	1	A	C	0.46	-0.0129679	0.0411189	0.752
3	*rs1260326*	2	T	C	0.4	-0.0447251	0.0419132	0.286
4	*rs17050272*	2	A	G	0.41	0.0478248	0.0416897	0.251
5	*rs6770152**	3	G	T	0.42	0.0157418	0.0434323	0.717
6	*rs12498742*	4	A	G	0.77	0.0657873	0.0486288	0.176
7	*rs2231142*	4	T	G	0.11	0.135012	0.0641224	0.035
8	*rs17632159**	5	G	C	0.7	-0.0585164	0.0445821	0.189
9	*rs1165151**	6	G	T	0.55	0.03386	0.0412111	0.411
10	*rs675209*	6	T	C	0.27	-0.0462202	0.0462473	0.318
11	*rs729761**	6	G	T	0.71	-0.0135494	0.0457052	0.767
12	*rs10480300*	7	T	C	0.27	0.0583149	0.0460002	0.205
13	*rs1178977*	7	A	G	0.8	0.00635303	0.0515263	0.902
14	*rs17786744**	8	G	A	0.41	-0.0474304	0.041771	0.256
15	*rs2941484*	8	T	C	0.45	0.00659176	0.0414914	0.874
16	*rs10821905*	10	A	G	0.18	0.0204743	0.0534606	0.702
17	*rs1171614**	10	C	T	0.77	0.0878629	0.0486846	0.071
18	*rs2078267**	11	C	T	0.45	0.0351306	0.0411342	0.393
19	*rs478607**	11	G	A	0.15	0.00625879	0.0570411	0.913
20	*rs3741414**	12	C	T	0.76	0.0606133	0.047814	0.205
21	*rs653178**	12	C	T	0.48	0.0452538	0.0410345	0.27
22	*rs1394125*	15	A	G	0.36	0.0277236	0.0426754	0.516
23	*rs6598541*	15	A	G	0.36	-0.0266668	0.0429041	0.534
24	*rs7188445**	16	G	A	0.67	0.00154779	0.0436978	0.972
25	*rs7193778**	16	C	T	0.15	-0.0834278	0.0574108	0.146
26	*rs7224610**	17	C	A	0.4	-0.0082339	0.0422171	0.845

SNP: SNP number; Chr: chromosome; EA: effect allele; OA: other allele; EAF: effect allele frequency; G: genetic variant or SNP, Y: outcome (cancer in this case); GY: beta for the SNP-cancer association; SE-GY: standard error of GY; *p*: p-value of GY.
^Estimates are adjusted for age, sex and first principal component.
*The direction of EA was corrected for the urate raising allele prior to analyses.
SNP-cancer association analyses were done after excluding controls.

Table S26: The association estimates of the 26 SNPs for urate with hepatic cancer in the UK-Biobank cohort

	SNP	Chr	EA	OA	EAF	GY^	SE-GY	*p*
1	*rs11264341**	1	C	T	0.57	-0.138817	0.0649915	0.033
2	*rs1471633*	1	A	C	0.46	0.0934926	0.0644691	0.147
3	*rs1260326*	2	T	C	0.4	-0.0170659	0.0657051	0.795
4	*rs17050272*	2	A	G	0.41	-0.0471308	0.0653789	0.471
5	*rs6770152**	3	G	T	0.42	0.087375	0.0680351	0.199
6	*rs12498742*	4	A	G	0.77	-0.0014903	0.07613	0.984
7	*rs2231142*	4	T	G	0.11	0.119685	0.100923	0.236
8	*rs17632159**	5	G	C	0.7	-0.0577133	0.0699623	0.409
9	*rs1165151**	6	G	T	0.55	-0.0330467	0.0646061	0.609
10	*rs675209*	6	T	C	0.27	9.0761E-05	0.0724357	0.999
11	*rs729761**	6	G	T	0.71	-0.0909478	0.0715683	0.204
12	*rs10480300*	7	T	C	0.27	-0.0578611	0.0719663	0.421
13	*rs1178977*	7	A	G	0.8	-0.0614227	0.0809019	0.448
14	*rs17786744**	8	G	A	0.41	-0.067183	0.0656544	0.306
15	*rs2941484*	8	T	C	0.45	-0.0693831	0.0649989	0.286
16	*rs10821905*	10	A	G	0.18	-0.0098332	0.0839386	0.907
17	*rs1171614**	10	C	T	0.77	0.0258793	0.0763426	0.735
18	*rs2078267**	11	C	T	0.45	-0.0779862	0.0645124	0.227
19	*rs478607**	11	G	A	0.15	0.134637	0.0895584	0.133
20	*rs3741414**	12	C	T	0.76	0.0731873	0.0751826	0.33
21	*rs653178**	12	C	T	0.48	0.0927573	0.0643578	0.15
22	*rs1394125*	15	A	G	0.36	0.0172362	0.066859	0.797
23	*rs6598541*	15	A	G	0.36	-0.0358362	0.067224	0.594
24	*rs7188445**	16	G	A	0.67	0.197309	0.0686039	0.004
25	*rs7193778**	16	C	T	0.15	-0.0776846	0.0902	0.389
26	*rs7224610**	17	C	A	0.4	0.0501786	0.0660375	0.447

SNP: SNP number; Chr: chromosome; EA: effect allele; OA: other allele; EAF: effect allele frequency; G: genetic variant or SNP, Y: outcome (cancer in this case); GY: beta for the SNP-cancer association; SE-GY: standard error of GY; *p*: p-value of GY.
^Estimates are adjusted for age, sex and first principal component.
*The direction of EA was corrected for the urate raising allele prior to analyses.
SNP-cancer association analyses were done after excluding controls.

Table S27: The association estimates of the 26 SNPs for urate with renal cancer in the UK-Biobank cohort

	SNP	Chr	EA	OA	EAF	GY^	SE-GY	*p*
1	*rs11264341**	1	C	T	0.57	0.0482911	0.0407233	0.236
2	*rs1471633*	1	A	C	0.46	0.0158273	0.0404301	0.695
3	*rs1260326*	2	T	C	0.4	-0.0105388	0.041227	0.798
4	*rs17050272*	2	A	G	0.41	0.00105173	0.0409721	0.98
5	*rs6770152**	3	G	T	0.42	0.00104742	0.0427312	0.98
6	*rs12498742*	4	A	G	0.77	-0.0383826	0.0477258	0.421
7	*rs2231142*	4	T	G	0.11	-0.095564	0.063461	0.132
8	*rs17632159**	5	G	C	0.7	-0.0030301	0.0439407	0.945
9	*rs1165151**	6	G	T	0.55	-0.0293611	0.0405218	0.469
10	*rs675209*	6	T	C	0.27	0.0646995	0.0454755	0.155
11	*rs729761**	6	G	T	0.71	-0.0147553	0.0449357	0.743
12	*rs10480300*	7	T	C	0.27	-0.0135677	0.0452713	0.764
13	*rs1178977*	7	A	G	0.8	0.0327072	0.0508108	0.52
14	*rs17786744**	8	G	A	0.41	-0.0219039	0.0410946	0.594
15	*rs2941484*	8	T	C	0.45	-0.0091608	0.0408222	0.822
16	*rs10821905*	10	A	G	0.18	-0.0186474	0.0527401	0.724
17	*rs1171614**	10	C	T	0.77	0.00542633	0.047903	0.91
18	*rs2078267**	11	C	T	0.45	-0.029248	0.040467	0.47
19	*rs478607**	11	G	A	0.15	0.150222	0.0560586	0.007
20	*rs3741414**	12	C	T	0.76	-0.0134671	0.0469733	0.774
21	*rs653178**	12	C	T	0.48	0.0260457	0.0403606	0.519
22	*rs1394125*	15	A	G	0.36	-0.0307473	0.0420022	0.464
23	*rs6598541*	15	A	G	0.36	0.0446463	0.0422106	0.29
24	*rs7188445**	16	G	A	0.67	0.0370081	0.0429286	0.389
25	*rs7193778**	16	C	T	0.15	0.0259711	0.0566608	0.647
26	*rs7224610**	17	C	A	0.4	-0.0128206	0.0415007	0.757

SNP: SNP number; Chr: chromosome; EA: effect allele; OA: other allele; EAF: effect allele frequency; G: genetic variant or SNP, Y: outcome (cancer in this case); GY: beta for the SNP-cancer association; SE-GY: standard error of GY; *p*: p-value of GY.
^Estimates are adjusted for age, sex and first principal component.
*The direction of EA was corrected for the urate raising allele prior to analyses.
SNP-cancer association analyses were done after excluding controls.

Table S28: The association estimates of the 26 SNPs for urate with lung cancer in the UK-Biobank cohort

	SNP	Chr	EA	OA	EAF	GY^	SE-GY	*p*
1	*rs11264341**	1	C	T	0.57	0.0567864	0.0282909	0.045
2	*rs1471633*	1	A	C	0.46	0.0256349	0.02807	0.361
3	*rs1260326*	2	T	C	0.4	-0.0264675	0.0286108	0.355
4	*rs17050272*	2	A	G	0.41	-0.0037085	0.0284835	0.896
5	*rs6770152**	3	G	T	0.42	-0.0158941	0.02959	0.591
6	*rs12498742*	4	A	G	0.77	-0.0458215	0.0331703	0.167
7	*rs2231142*	4	T	G	0.11	-0.0768901	0.0438082	0.079
8	*rs17632159**	5	G	C	0.7	-0.018428	0.030435	0.545
9	*rs1165151**	6	G	T	0.55	0.0385538	0.0281246	0.17
10	*rs675209*	6	T	C	0.27	0.0533537	0.0315347	0.091
11	*rs729761**	6	G	T	0.71	0.00257797	0.0311356	0.934
12	*rs10480300*	7	T	C	0.27	0.0176198	0.0313517	0.574
13	*rs1178977*	7	A	G	0.8	-0.0281266	0.0351451	0.424
14	*rs17786744**	8	G	A	0.41	0.01002	0.0285344	0.725
15	*rs2941484*	8	T	C	0.45	0.0153774	0.0283115	0.587
16	*rs10821905*	10	A	G	0.18	0.0592263	0.0364927	0.105
17	*rs1171614**	10	C	T	0.77	0.00697656	0.0332552	0.834
18	*rs2078267**	11	C	T	0.45	0.00034574	0.0280983	0.99
19	*rs478607**	11	G	A	0.15	-0.0132543	0.0389506	0.734
20	*rs3741414**	12	C	T	0.76	0.0204087	0.0327111	0.533
21	*rs653178**	12	C	T	0.48	-0.0040644	0.0279871	0.885
22	*rs1394125*	15	A	G	0.36	-0.116681	0.0291308	6.19E-05
23	*rs6598541*	15	A	G	0.36	-0.0597755	0.0293209	0.041
24	*rs7188445**	16	G	A	0.67	-0.0255103	0.0298533	0.393
25	*rs7193778**	16	C	T	0.15	-0.0350083	0.0392647	0.373
26	*rs7224610**	17	C	A	0.4	-0.0202304	0.0288272	0.483

SNP: SNP number; Chr: chromosome; EA: effect allele; OA: other allele; EAF: effect allele frequency; G: genetic variant or SNP, Y: outcome (cancer in this case); GY: beta for the SNP-cancer association; SE-GY: standard error of GY; *p*: p-value of GY.
^Estimates are adjusted for age, sex and first principal component.
*The direction of EA was corrected for the urate raising allele prior to analyses.
SNP-cancer association analyses were done after excluding controls.

Table S29: The association estimates of the 26 SNPs for urate with lymphatic and hematopoietic cancer in the UK-Biobank cohort

	SNP	Chr	EA	OA	EAF	GY^	SE-GY	*p*
1	*rs11264341**	1	C	T	0.57	0.00152829	0.014854	0.918
2	*rs1471633*	1	A	C	0.46	-0.0034634	0.0147533	0.814
3	*rs1260326*	2	T	C	0.4	-0.0293131	0.0150376	0.051
4	*rs17050272*	2	A	G	0.41	0.0265085	0.0149571	0.076
5	*rs6770152**	3	G	T	0.42	0.00603995	0.01557	0.698
6	*rs12498742*	4	A	G	0.77	-0.0133435	0.0173973	0.443
7	*rs2231142*	4	T	G	0.11	-0.0077337	0.0231338	0.738
8	*rs17632159**	5	G	C	0.7	0.0002107	0.0160164	0.99
9	*rs1165151**	6	G	T	0.55	0.00322101	0.0147807	0.827
10	*rs675209*	6	T	C	0.27	0.0290523	0.0165654	0.079
11	*rs729761**	6	G	T	0.71	0.0253976	0.0163833	0.121
12	*rs10480300*	7	T	C	0.27	0.0381005	0.0164836	0.021
13	*rs1178977*	7	A	G	0.8	-0.0099856	0.0185023	0.589
14	*rs17786744**	8	G	A	0.41	-0.0319412	0.0150048	0.033
15	*rs2941484*	8	T	C	0.45	0.0243124	0.0148718	0.102
16	*rs10821905*	10	A	G	0.18	0.0154483	0.0192251	0.422
17	*rs1171614**	10	C	T	0.77	-0.0039726	0.0174747	0.82
18	*rs2078267**	11	C	T	0.45	-0.0120105	0.0147774	0.416
19	*rs478607**	11	G	A	0.15	-0.0117025	0.0204579	0.567
20	*rs3741414**	12	C	T	0.76	0.0100397	0.0171687	0.559
21	*rs653178**	12	C	T	0.48	0.0589451	0.0147092	6.10E-05
22	*rs1394125*	15	A	G	0.36	0.00344213	0.0152962	0.822
23	*rs6598541*	15	A	G	0.36	0.00956543	0.0154014	0.535
24	*rs7188445**	16	G	A	0.67	-0.0228147	0.015692	0.146
25	*rs7193778**	16	C	T	0.15	-0.0519027	0.0206858	0.012
26	*rs7224610**	17	C	A	0.4	0.0169003	0.0151607	0.265

SNP: SNP number; Chr: chromosome; EA: effect allele; OA: other allele; EAF: effect allele frequency; G: genetic variant or SNP, Y: outcome (cancer in this case); GY: beta for the SNP-cancer association; SE-GY: standard error of GY; *p*: p-value of GY.
^Estimates are adjusted for age, sex and first principal component.
*The direction of EA was corrected for the urate raising allele prior to analyses.
SNP-cancer association analyses were done after excluding controls.

Table S30: The association estimates of the 26 SNPs for urate with pancreatic cancer in the UK-Biobank cohort

	SNP	Chr	EA	OA	EAF	GY^	SE-GY	*p*
1	*rs11264341**	1	C	T	0.57	-0.116291	0.0496183	0.821
2	*rs1471633*	1	A	C	0.46	-0.0569525	0.0492171	0.051
3	*rs1260326*	2	T	C	0.4	-0.0702754	0.0501664	0.019
4	*rs17050272*	2	A	G	0.41	-0.0983446	0.0498833	0.026
5	*rs6770152**	3	G	T	0.42	0.00186189	0.052026	0.633
6	*rs12498742*	4	A	G	0.77	0.0282765	0.0581318	0.916
7	*rs2231142*	4	T	G	0.11	0.015332	0.0773472	0.627
8	*rs17632159**	5	G	C	0.7	-0.0297058	0.0534167	0.161
9	*rs1165151**	6	G	T	0.55	-0.109984	0.0493157	0.879
10	*rs675209*	6	T	C	0.27	0.0661162	0.055293	0.247
11	*rs729761**	6	G	T	0.71	0.0778539	0.0548097	0.049
12	*rs10480300*	7	T	C	0.27	-0.0124271	0.0549831	0.578
13	*rs1178977*	7	A	G	0.8	-0.0064923	0.061791	0.648
14	*rs17786744**	8	G	A	0.41	-0.0228268	0.050061	0.923
15	*rs2941484*	8	T	C	0.45	0.0449855	0.0496983	0.843
16	*rs10821905*	10	A	G	0.18	-0.125183	0.0642501	0.365
17	*rs1171614**	10	C	T	0.77	-0.0278507	0.0583454	0.663
18	*rs2078267**	11	C	T	0.45	0.00475839	0.049263	0.535
19	*rs478607**	11	G	A	0.15	0.0422727	0.0681897	0.399
20	*rs3741414**	12	C	T	0.76	0.0249529	0.057344	0.193
21	*rs653178**	12	C	T	0.48	-0.0413498	0.0490939	0.232
22	*rs1394125*	15	A	G	0.36	0.00775193	0.0510796	0.971
23	*rs6598541*	15	A	G	0.36	0.0669744	0.0514428	0.675
24	*rs7188445**	16	G	A	0.67	-0.0219719	0.0523401	0.076
25	*rs7193778**	16	C	T	0.15	-0.122547	0.0691511	0.655
26	*rs7224610**	17	C	A	0.4	-0.0226111	0.0505959	0.155

SNP: SNP number; Chr: chromosome; EA: effect allele; OA: other allele; EAF: effect allele frequency; G: genetic variant or SNP, Y: outcome (cancer in this case); GY: beta for the SNP-cancer association; SE-GY: standard error of GY; *p*: p-value of GY.
^Estimates are adjusted for age, sex and first principal component.
*The direction of EA was corrected for the urate raising allele prior to analyses.
SNP-cancer association analyses were done after excluding controls.

Table S31: The association estimates of the 26 SNPs for urate with skin cancer in the UK-Biobank cohort

	SNP	Chr	EA	OA	EAF	GY^	SE-GY	*p*
1	*rs11264341**	1	C	T	0.57	-0.0132534	0.0301476	0.66
2	*rs1471633*	1	A	C	0.46	0.012298	0.0299561	0.681
3	*rs1260326*	2	T	C	0.4	0.0507123	0.0305305	0.097
4	*rs17050272*	2	A	G	0.41	-0.0296365	0.0303578	0.329
5	*rs6770152**	3	G	T	0.42	0.005594	0.0316384	0.86
6	*rs12498742*	4	A	G	0.77	-0.0316504	0.035312	0.37
7	*rs2231142*	4	T	G	0.11	-0.0084096	0.047026	0.858
8	*rs17632159**	5	G	C	0.7	-0.015103	0.0325375	0.643
9	*rs1165151**	6	G	T	0.55	-0.0295459	0.0300098	0.325
10	*rs675209*	6	T	C	0.27	-0.0321185	0.0336396	0.34
11	*rs729761**	6	G	T	0.71	-0.034244	0.0332941	0.304
12	*rs10480300*	7	T	C	0.27	0.105069	0.0335192	0.002
13	*rs1178977*	7	A	G	0.8	0.0494598	0.0375608	0.188
14	*rs17786744**	8	G	A	0.41	-0.0034805	0.0304401	0.909
15	*rs2941484*	8	T	C	0.45	-0.056802	0.0302004	0.06
16	*rs10821905*	10	A	G	0.18	-0.016603	0.0390974	0.671
17	*rs1171614**	10	C	T	0.77	0.0534164	0.035466	0.132
18	*rs2078267**	11	C	T	0.45	-0.0297481	0.0300008	0.321
19	*rs478607**	11	G	A	0.15	0.00092441	0.0414827	0.982
20	*rs3741414**	12	C	T	0.76	-0.0733915	0.0347885	0.035
21	*rs653178**	12	C	T	0.48	0.057602	0.0298604	0.054
22	*rs1394125*	15	A	G	0.36	-0.0132397	0.0310671	0.67
23	*rs6598541*	15	A	G	0.36	0.064992	0.031273	0.038
24	*rs7188445**	16	G	A	0.67	0.0108202	0.0318196	0.734
25	*rs7193778**	16	C	T	0.15	-0.0010791	0.0420429	0.98
26	*rs7224610**	17	C	A	0.4	-0.0189132	0.0307841	0.539

SNP: SNP number; Chr: chromosome; EA: effect allele; OA: other allele; EAF: effect allele frequency; G: genetic variant or SNP, Y: outcome (cancer in this case); GY: beta for the SNP-cancer association; SE-GY: standard error of GY; *p*: p-value of GY.
^Estimates are adjusted for age, sex and first principal component.
*The direction of EA was corrected for the urate raising allele prior to analyses.
SNP-cancer association analyses were done after excluding controls.

Table S32: The association estimates of the 26 SNPs for urate with breast cancer in the UK-Biobank cohort

	SNP	Chr	EA	OA	EAF	GY^	SE-GY	*p*
1	*rs11264341**	1	C	T	0.57	0.0339365	0.0145429	0.02
2	*rs1471633*	1	A	C	0.46	0.016186	0.0144321	0.262
3	*rs1260326*	2	T	C	0.4	-0.0160701	0.0147215	0.275
4	*rs17050272*	2	A	G	0.41	-0.0061773	0.0146452	0.673
5	*rs6770152**	3	G	T	0.42	0.00115236	0.0152483	0.94
6	*rs12498742*	4	A	G	0.77	0.0011449	0.017017	0.946
7	*rs2231142*	4	T	G	0.11	-0.0530077	0.0227263	0.02
8	*rs17632159**	5	G	C	0.7	-0.0152987	0.0156742	0.329
9	*rs1165151**	6	G	T	0.55	-0.0038493	0.0144672	0.79
10	*rs675209*	6	T	C	0.27	-0.0231559	0.0161967	0.153
11	*rs729761**	6	G	T	0.71	-0.0134652	0.0160505	0.402
12	*rs10480300*	7	T	C	0.27	0.0268811	0.0161282	0.096
13	*rs1178977*	7	A	G	0.8	0.0195769	0.0180909	0.279
14	*rs17786744**	8	G	A	0.41	-0.0028033	0.0146874	0.849
15	*rs2941484*	8	T	C	0.45	0.0269707	0.0145613	0.064
16	*rs10821905*	10	A	G	0.18	0.00605839	0.0188382	0.748
17	*rs1171614**	10	C	T	0.77	-0.0119937	0.0170911	0.483
18	*rs2078267**	11	C	T	0.45	0.015342	0.0144914	0.29
19	*rs478607**	11	G	A	0.15	-0.0206202	0.0199853	0.302
20	*rs3741414**	12	C	T	0.76	0.031245	0.0167988	0.063
21	*rs653178**	12	C	T	0.48	0.055312	0.014376	1.19E-04
22	*rs1394125*	15	A	G	0.36	0.00975224	0.0149679	0.515
23	*rs6598541*	15	A	G	0.36	0.0308479	0.0150945	0.041
24	*rs7188445**	16	G	A	0.67	-0.0128371	0.0153769	0.404
25	*rs7193778**	16	C	T	0.15	-0.0519596	0.0203027	0.01
26	*rs7224610**	17	C	A	0.4	0.00353648	0.0148585	0.812

SNP: SNP number; Chr: chromosome; EA: effect allele; OA: other allele; EAF: effect allele frequency; G: genetic variant or SNP, Y: outcome (cancer in this case); GY: beta for the SNP-cancer association; SE-GY: standard error of GY; *p*: p-value of GY.
^Estimates are adjusted for age, sex and first principal component.
*The direction of EA was corrected for the urate raising allele prior to analyses.
SNP-cancer association analyses were done after excluding controls.

Table S33: The association estimates of the 26 SNPs for urate with gynecological cancers in the UK-Biobank cohort

	SNP	Chr	EA	OA	EAF	GY^	SE-GY	*p*
1	*rs11264341**	1	C	T	0.57	-0.0532007	0.0280619	0.058
2	*rs1471633*	1	A	C	0.46	0.019734	0.0278484	0.479
3	*rs1260326*	2	T	C	0.4	-0.0141366	0.028401	0.619
4	*rs17050272*	2	A	G	0.41	-0.0067409	0.0282729	0.812
5	*rs6770152**	3	G	T	0.42	0.0611636	0.0294431	0.038
6	*rs12498742*	4	A	G	0.77	-0.017372	0.0328317	0.597
7	*rs2231142*	4	T	G	0.11	-0.0496248	0.0436996	0.256
8	*rs17632159**	5	G	C	0.7	-0.0135717	0.0302614	0.654
9	*rs1165151**	6	G	T	0.55	0.053074	0.0279024	0.057
10	*rs675209*	6	T	C	0.27	0.0171589	0.03124	0.583
11	*rs729761**	6	G	T	0.71	0.010903	0.0309661	0.725
12	*rs10480300*	7	T	C	0.27	-0.0075009	0.0311222	0.81
13	*rs1178977*	7	A	G	0.8	0.00445343	0.034889	0.898
14	*rs17786744**	8	G	A	0.41	0.00122439	0.0283346	0.966
15	*rs2941484*	8	T	C	0.45	0.0165698	0.0280825	0.555
16	*rs10821905*	10	A	G	0.18	-0.045298	0.0364035	0.213
17	*rs1171614**	10	C	T	0.77	-0.0599029	0.0329747	0.069
18	*rs2078267**	11	C	T	0.45	-0.0066049	0.0279599	0.813
19	*rs478607**	11	G	A	0.15	0.00082503	0.0385639	0.983
20	*rs3741414**	12	C	T	0.76	-0.005324	0.0323978	0.869
21	*rs653178**	12	C	T	0.48	0.0327483	0.0277188	0.237
22	*rs1394125*	15	A	G	0.36	0.0049759	0.0288726	0.863
23	*rs6598541*	15	A	G	0.36	-0.0051284	0.0291346	0.86
24	*rs7188445**	16	G	A	0.67	-0.0225596	0.0296696	0.447
25	*rs7193778**	16	C	T	0.15	-0.0412211	0.0391253	0.292
26	*rs7224610**	17	C	A	0.4	0.0284163	0.0286465	0.321

SNP: SNP number; Chr: chromosome; EA: effect allele; OA: other allele; EAF: effect allele frequency; G: genetic variant or SNP, Y: outcome (cancer in this case); GY: beta for the SNP-cancer association; SE-GY: standard error of GY; *p*: p-value of GY.

^Estimates are adjusted for age, sex and first principal component.

*The direction of EA was corrected for the urate raising allele prior to analyses.

SNP-cancer association analyses were done after excluding controls.

Table S34: The association estimates of the 26 SNPs for urate with prostate cancer in the UK-Biobank cohort

	SNP	Chr	EA	OA	EAF	GY^	SE-GY	*p*
1	*rs11264341**	1	C	T	0.57	0.0141908	0.0184193	0.252
2	*rs1471633*	1	A	C	0.46	-0.0179301	0.0182801	0.717
3	*rs1260326*	2	T	C	0.4	-0.0051067	0.0186354	0.441
4	*rs17050272*	2	A	G	0.41	-0.0016628	0.0185039	0.644
5	*rs6770152**	3	G	T	0.42	-0.0163893	0.0192746	0.158
6	*rs12498742*	4	A	G	0.77	-0.0051854	0.0215874	0.387
7	*rs2231142*	4	T	G	0.11	-0.0260415	0.0287398	0.81
8	*rs17632159**	5	G	C	0.7	-0.013901	0.0198219	0.784
9	*rs1165151**	6	G	T	0.55	-0.0084744	0.0183315	0.264
10	*rs675209*	6	T	C	0.27	-0.0013849	0.020579	0.327
11	*rs729761**	6	G	T	0.71	0.00589699	0.0202883	0.928
12	*rs10480300*	7	T	C	0.27	0.0234225	0.0204386	0.483
13	*rs1178977*	7	A	G	0.8	0.0198997	0.0229819	0.061
14	*rs17786744**	8	G	A	0.41	-0.0348108	0.0185579	0.227
15	*rs2941484*	8	T	C	0.45	-0.0099068	0.0184603	0.365
16	*rs10821905*	10	A	G	0.18	-0.0086263	0.0237772	0.592
17	*rs1171614**	10	C	T	0.77	0.0306369	0.0216753	0.104
18	*rs2078267**	11	C	T	0.45	-0.0220424	0.0182544	0.926
19	*rs478607**	11	G	A	0.15	0.00235266	0.0253732	0.773
20	*rs3741414**	12	C	T	0.76	-0.034557	0.0212837	0.7
21	*rs653178**	12	C	T	0.48	-0.0052659	0.018244	0.946
22	*rs1394125*	15	A	G	0.36	0.0212029	0.0189863	0.395
23	*rs6598541*	15	A	G	0.36	-0.0073332	0.0190537	0.326
24	*rs7188445**	16	G	A	0.67	0.0190304	0.0193823	0.194
25	*rs7193778**	16	C	T	0.15	-0.033285	0.0256088	0.618
26	*rs7224610**	17	C	A	0.4	-0.0093578	0.0187632	0.771

SNP: SNP number; Chr: chromosome; EA: effect allele; OA: other allele; EAF: effect allele frequency; G: genetic variant or SNP, Y: outcome (cancer in this case); GY: beta for the SNP-cancer association; SE-GY: standard error of GY; *p*: p-value of GY.
^Estimates are adjusted for age, sex and first principal component.
*The direction of EA was corrected for the urate raising allele prior to analyses.
SNP-cancer association analyses were done after excluding controls.

Table S35: The association estimates of the 26 SNPs for urate with all-cause cancer in the UK-Biobank cohort

	SNP	Chr	EA	OA	EAF	GY^	SE-GY	*p*
1	*rs11264341**	1	C	T	0.57	0.00657421	0.00794928	0.013
2	*rs1471633*	1	A	C	0.46	-0.0001384	0.00789576	0.141
3	*rs1260326*	2	T	C	0.4	-0.0077912	0.00804917	0.408
4	*rs17050272*	2	A	G	0.41	-0.0043466	0.00800426	0.557
5	*rs6770152**	3	G	T	0.42	0.0149524	0.00834099	0.794
6	*rs12498742*	4	A	G	0.77	-0.011096	0.00931182	0.856
7	*rs2231142*	4	T	G	0.11	-0.0353706	0.0124007	0.233
8	*rs17632159**	5	G	C	0.7	-0.0147381	0.00857488	0.333
9	*rs1165151**	6	G	T	0.55	0.00464205	0.00791341	0.734
10	*rs675209*	6	T	C	0.27	0.00037591	0.00886725	0.986
11	*rs729761**	6	G	T	0.71	-0.0013197	0.00877096	0.587
12	*rs10480300*	7	T	C	0.27	0.0219708	0.00882193	0.086
13	*rs1178977*	7	A	G	0.8	-0.0018021	0.00989756	0.004
14	*rs17786744**	8	G	A	0.41	-0.0234408	0.00802807	0.781
15	*rs2941484*	8	T	C	0.45	0.00743504	0.00796404	0.004
16	*rs10821905*	10	A	G	0.18	-0.0151794	0.0103041	0.351
17	*rs1171614**	10	C	T	0.77	0.00244047	0.00935105	0.444
18	*rs2078267**	11	C	T	0.45	0.00219449	0.00790731	0.946
19	*rs478607**	11	G	A	0.15	-0.0007424	0.0109455	2.78E-05
20	*rs3741414**	12	C	T	0.76	0.00702542	0.00918756	0.034
21	*rs653178**	12	C	T	0.48	0.0329874	0.00787135	0.966
22	*rs1394125*	15	A	G	0.36	-0.0027791	0.00818998	0.073
23	*rs6598541*	15	A	G	0.36	0.0174767	0.00824223	0.694
24	*rs7188445**	16	G	A	0.67	-0.0033104	0.0084007	0.005
25	*rs7193778**	16	C	T	0.15	-0.0311278	0.0110626	0.965
26	*rs7224610**	17	C	A	0.4	0.00035729	0.00811159	0.88

SNP: SNP number; Chr: chromosome; EA: effect allele; OA: other allele; EAF: effect allele frequency; G: genetic variant or SNP, Y: outcome (cancer in this case); GY: beta for the SNP-cancer association; SE-GY: standard error of GY; *p*: p-value of GY.
^Estimates are adjusted for age, sex and first principal component.
*The direction of EA was corrected for the urate raising allele prior to analyses.
SNP-cancer association analyses were done after excluding controls.

Table S36: Power calculations for MR analyses of the effect of serum urate on cancer risk

				UK-Biobank				MDCS-MPP			
	Cancer type	OR from observational analysis in MDCS-MPP	Type I error rate	% of variance in urate explained by 26 SNPs in GUGC	Sample size	Proportion of cases in UK-Biobank	Power (%)	% of variance in urate explained by 26 SNPs in MDCS-MPP	Sample size	Proportion of cases	Power (%)
**1**	Bladder	1.08	0.05	5.8	333000	0.0067	15	5.9	12454	0.0414	7
2	Colorectal	1.18	0.05	5.8	335243	0.0134	82	5.9	12729	0.0621	22
3	Brain	1.16	0.05	5.8	331344	0.0018	16	5.9	12054	0.0096	7
4	Gastric	0.92	0.05	5.8	331955	0.0036	10	5.9	12114	0.0145	6
5	Hepatic	1.01	0.05	5.8	331241	0.0015	5	5.9	11987	0.0041	5
6	Renal	1.01	0.05	5.8	331994	0.0037	5	5.9	12115	0.0146	5
7	Lung	0.94	0.05	5.8	333345	0.0078	11	5.9	12483	0.0437	6
8	Lymphatic and hematopoietic	1.02	0.05	5.8	340332	0.0281	7	5.9	12399	0.0372	5
9	Pancreatic	1.09	0.05	5.8	331590	0.0025	10	5.9	12060	0.0101	6
10	Skin	1.03	0.05	5.8	333019	0.0068	6	5.9	12331	0.0319	5
**11**	Breast	1.02	0.05	5.8	189222	0.0543	8	5.9	5364	0.1359	5
12	Gynecological	1.15	0.05	5.8	181590	0.0145	45	5.9	5000	0.0736	10
13	Prostate	1.08	0.05	5.8	158281	0.0409	33	5.9	8827	0.1723	10
**14**	All-cause	1.06	0.05	5.8	367570	0.1002	74	5.9	17597	0.3216	14

MDCS-MPP: Malmö Diet Cancer Study and Malmö Preventive Project, SNP: single neucclotide polymorphism

Supplementary figures

Figure S1 (a to n): Forest plots of MR estimates for the 26-SNPs, inverse variance weighted (IVW), weighted median and MR-Egger estimates for urate-cancer risk relationship in two-sample MR setting.

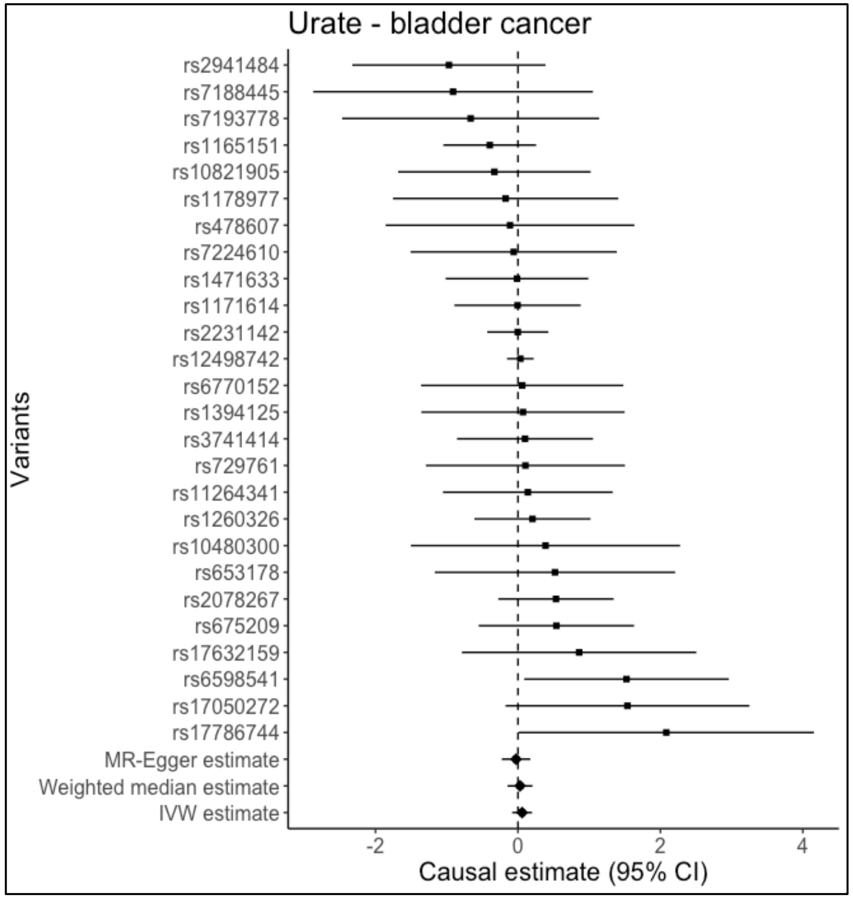

a)

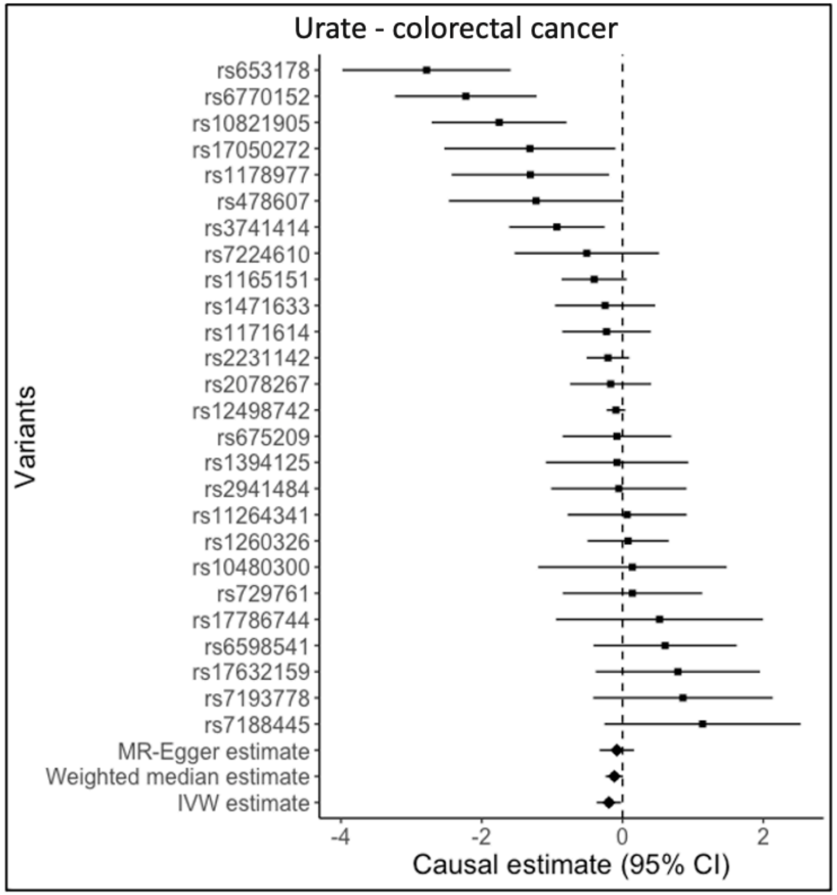

b)

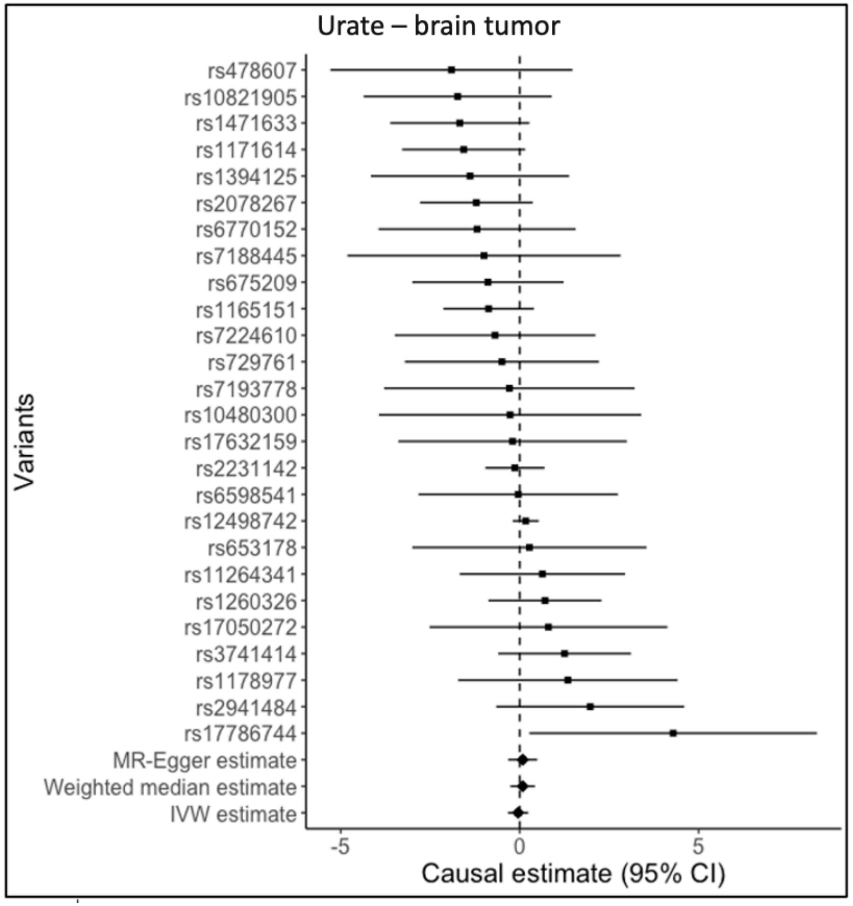
Urate – brain tumor
rs478607
rs10821905
rs1471633
rs1171614
rs1394125
rs2078267
rs6770152
rs7188445
rs675209
rs1165151
rs7224610
rs729761
rs7193778
rs10480300
rs17632159
rs2231142
rs6598541
rs12498742
rs653178
rs11264341
rs1260326
rs17050272
rs3741414
rs1178977
rs2941484
rs17786744
MR-Egger estimate
Weighted median estimate
IVW estimate
Variants
-5
0
5
Causal estimate (95% CI)

c)

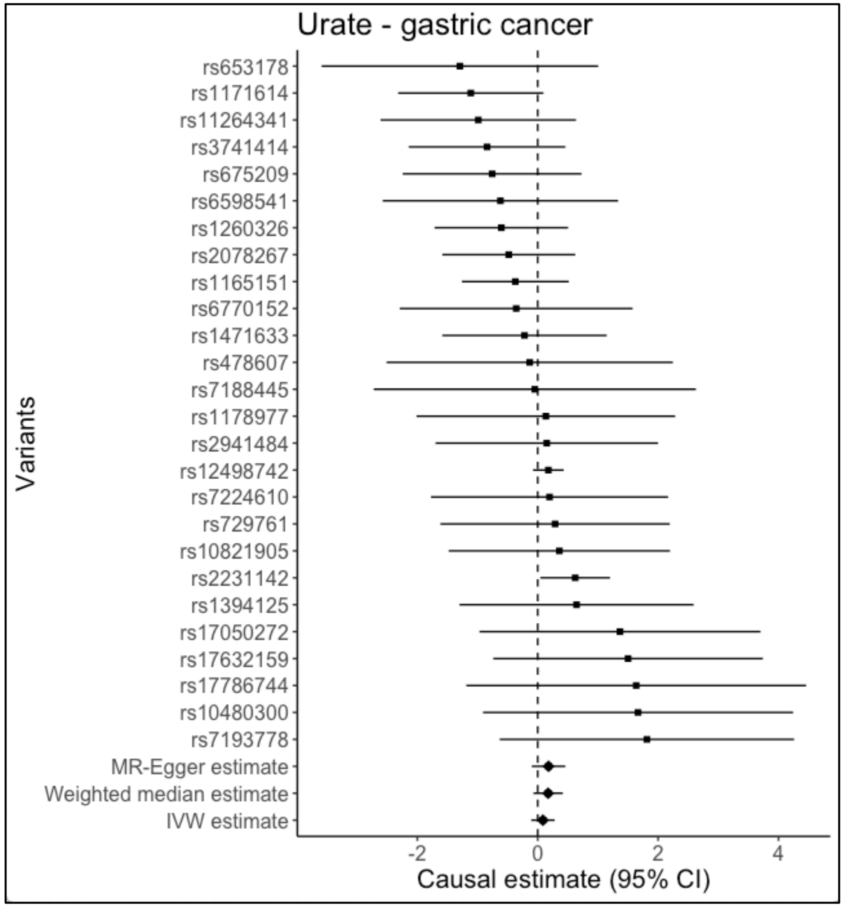
Urate - gastric cancer
rs653178
rs1171614
rs11264341
rs3741414
rs675209
rs6598541
rs1260326
rs2078267
rs1165151
rs6770152
rs1471633
rs478607
rs7188445
rs1178977
rs2941484
rs12498742
rs7224610
rs729761
rs10821905
rs2231142
rs1394125
rs17050272
rs17632159
rs17786744
rs10480300
rs7193778
MR-Egger estimate
Weighted median estimate
IVW estimate
Variants
-2
0
2
4
Causal estimate (95% CI)

d)

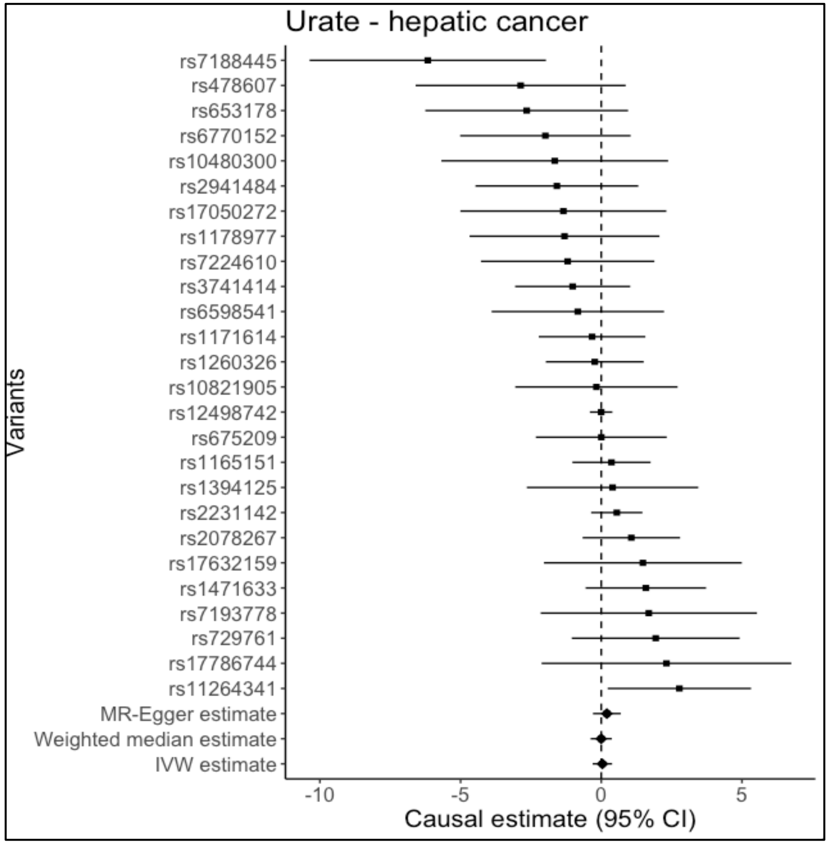
Urate - hepatic cancer
rs7188445
rs478607
rs653178
rs6770152
rs10480300
rs2941484
rs17050272
rs1178977
rs7224610
rs3741414
rs6598541
rs1171614
rs1260326
rs10821905
rs12498742
rs675209
rs1165151
rs1394125
rs2231142
rs2078267
rs17632159
rs1471633
rs7193778
rs729761
rs17786744
rs11264341
MR-Egger estimate
Weighted median estimate
IVW estimate
Variants
-10
-5
0
5
Causal estimate (95% CI)

e)

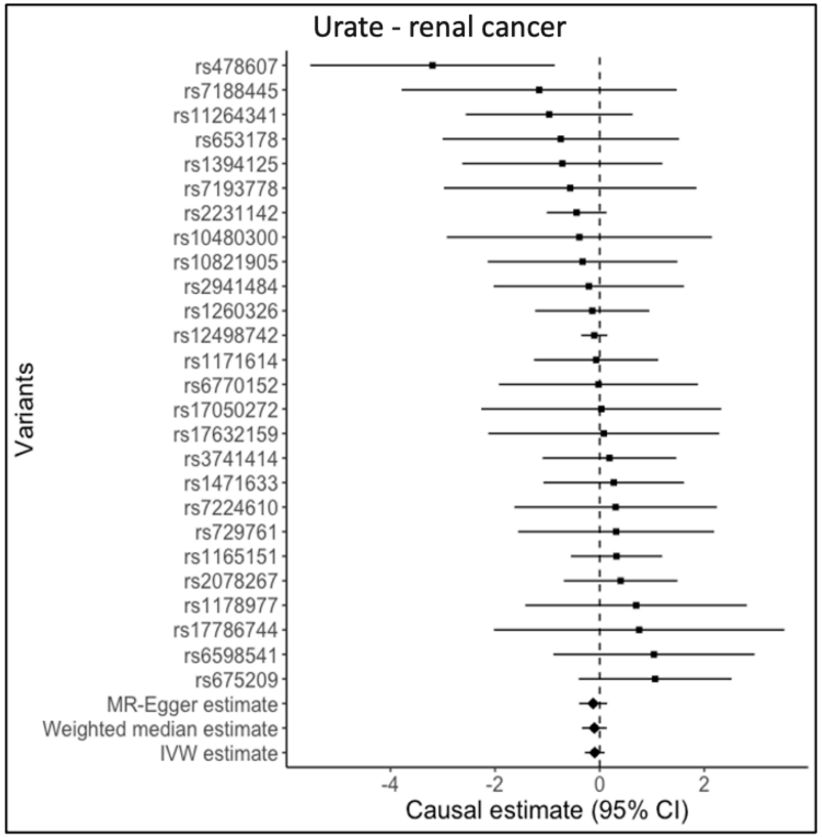
Urate - renal cancer
rs478607
rs7188445
rs11264341
rs653178
rs1394125
rs7193778
rs2231142
rs10480300
rs10821905
rs2941484
rs1260326
rs12498742
rs1171614
rs6770152
rs17050272
rs17632159
rs3741414
rs1471633
rs7224610
rs729761
rs1165151
rs2078267
rs1178977
rs17786744
rs6598541
rs675209
MR-Egger estimate
Weighted median estimate
IVW estimate
Variants
-4
-2
0
2
Causal estimate (95% CI)

f)

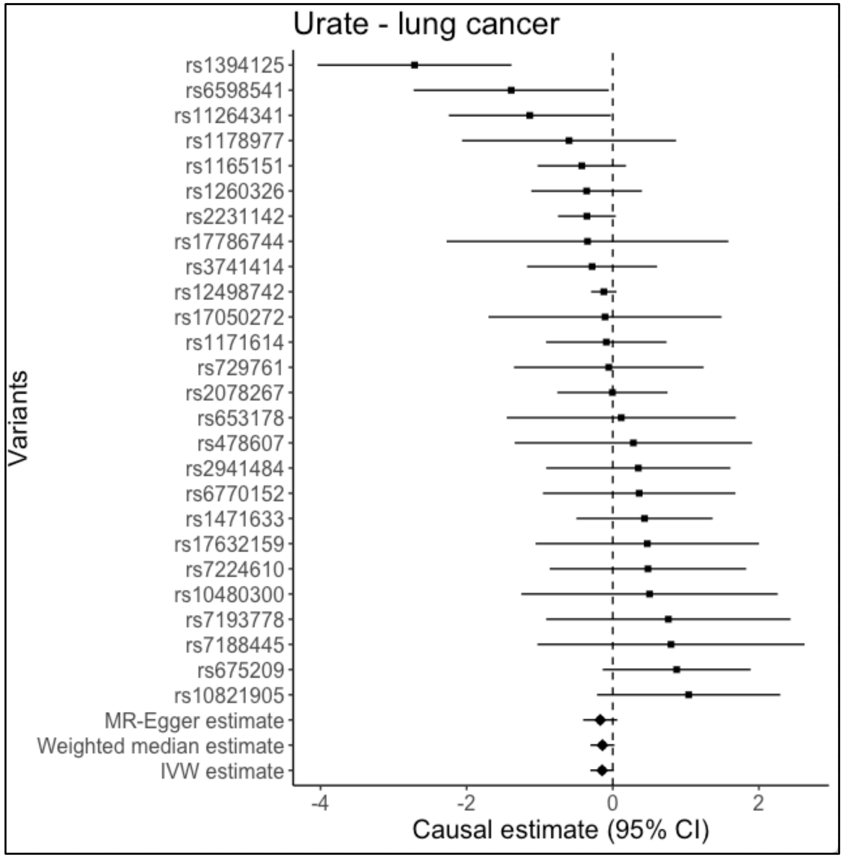
Urate - lung cancer
rs1394125
rs6598541
rs11264341
rs1178977
rs1165151
rs1260326
rs2231142
rs17786744
rs3741414
rs12498742
rs17050272
rs1171614
rs729761
rs2078267
rs653178
rs478607
rs2941484
rs6770152
rs1471633
rs17632159
rs7224610
rs10480300
rs7193778
rs7188445
rs675209
rs10821905
MR-Egger estimate
Weighted median estimate
IVW estimate
Variants
-4
-2
0
2
Causal estimate (95% CI)

g)

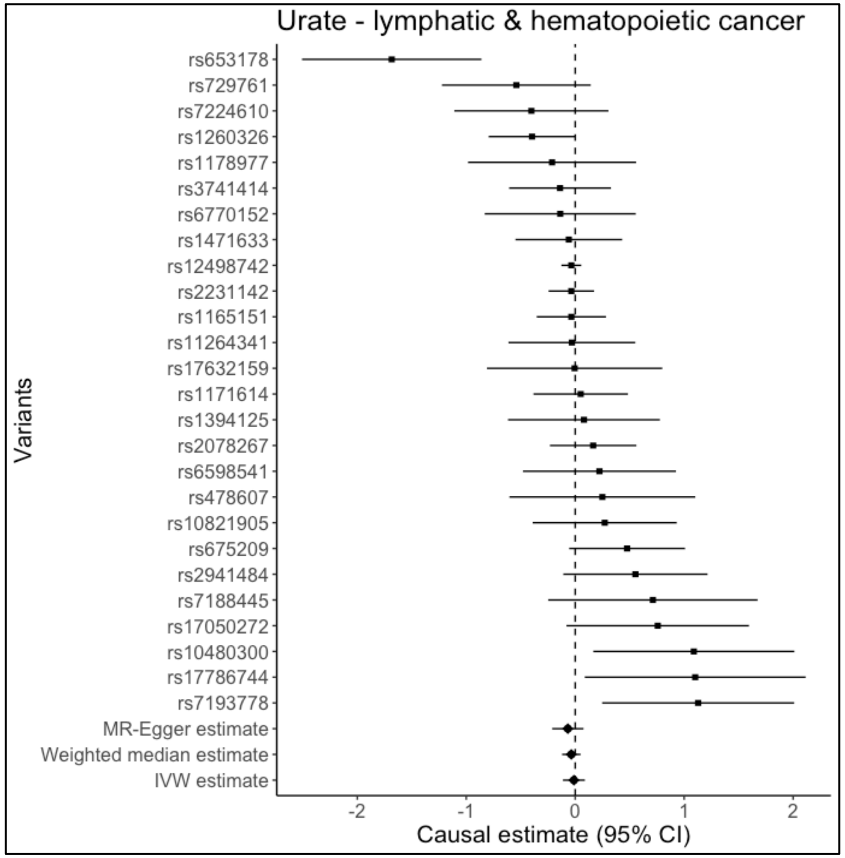
Urate - lymphatic & hematopoietic cancer
rs653178
rs729761
rs7224610
rs1260326
rs1178977
rs3741414
rs6770152
rs1471633
rs12498742
rs2231142
rs1165151
rs11264341
rs17632159
rs1171614
rs1394125
rs2078267
rs6598541
rs478607
rs10821905
rs675209
rs2941484
rs7188445
rs17050272
rs10480300
rs17786744
rs7193778
MR-Egger estimate
Weighted median estimate
IVW estimate
Variants
-2
-1
0
1
2
Causal estimate (95% CI)

h)

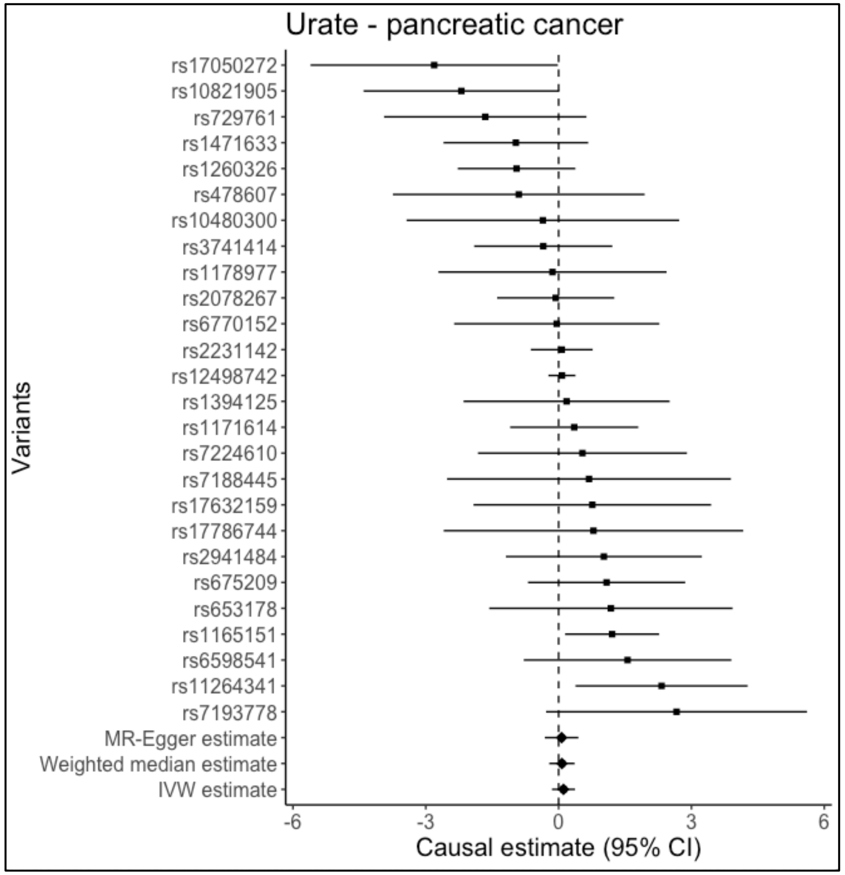
Urate - pancreatic cancer
rs17050272
rs10821905
rs729761
rs1471633
rs1260326
rs478607
rs10480300
rs3741414
rs1178977
rs2078267
rs6770152
rs2231142
rs12498742
rs1394125
rs1171614
rs7224610
rs7188445
rs17632159
rs17786744
rs2941484
rs675209
rs653178
rs1165151
rs6598541
rs11264341
rs7193778
MR-Egger estimate
Weighted median estimate
IVW estimate
Variants
-6
-3
0
3
6
Causal estimate (95% CI)

i)

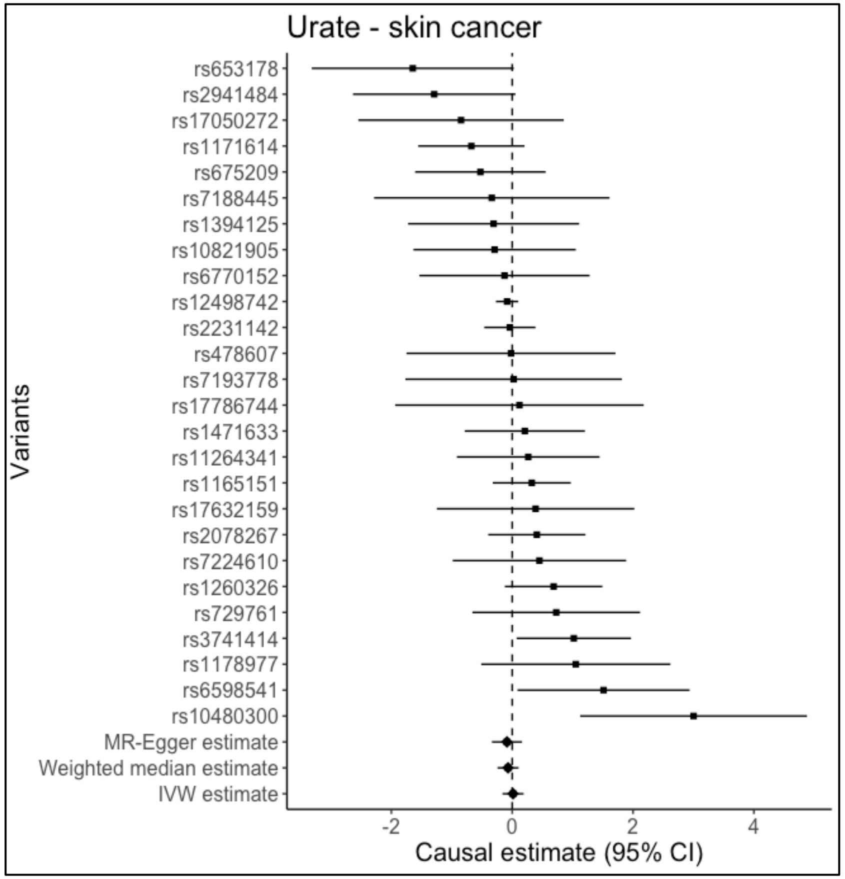
Urate - skin cancer
rs653178
rs2941484
rs17050272
rs1171614
rs675209
rs7188445
rs1394125
rs10821905
rs6770152
rs12498742
rs2231142
rs478607
rs7193778
rs17786744
rs1471633
rs11264341
rs1165151
rs17632159
rs2078267
rs7224610
rs1260326
rs729761
rs3741414
rs1178977
rs6598541
rs10480300
MR-Egger estimate
Weighted median estimate
IVW estimate
Variants
-2
0
2
4
Causal estimate (95% CI)

j)

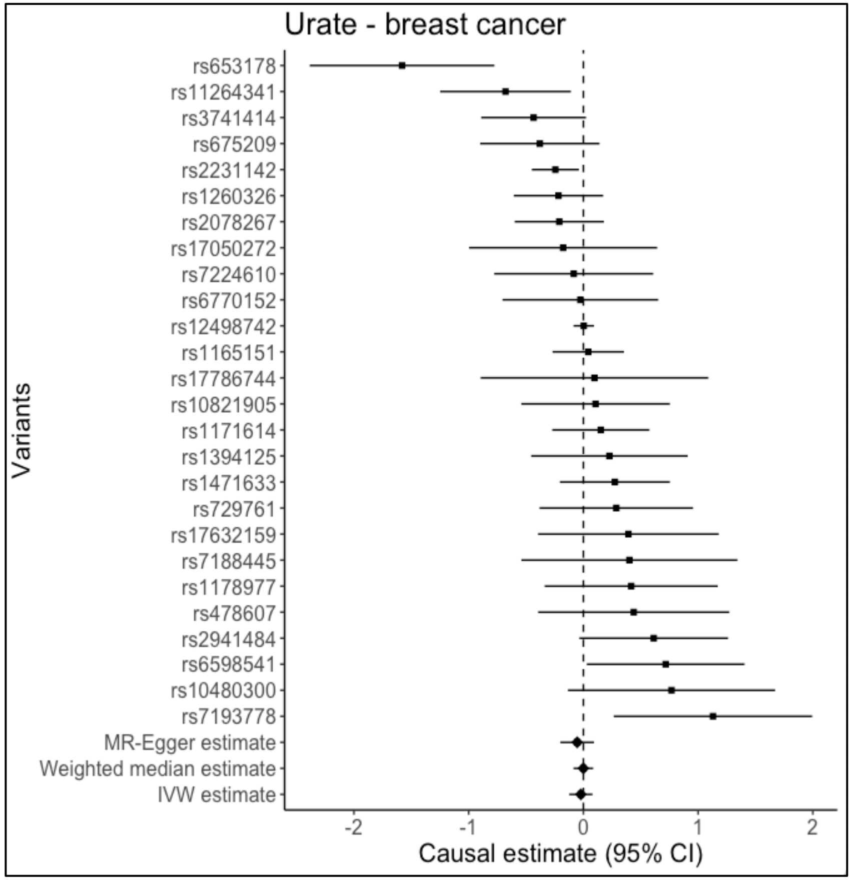

Urate - breast cancer
rs653178
rs11264341
rs3741414
rs675209
rs2231142
rs1260326
rs2078267
rs17050272
rs7224610
rs6770152
rs12498742
rs1165151
rs17786744
rs10821905
rs1171614
rs1394125
rs1471633
rs729761
rs17632159
rs7188445
rs1178977
rs478607
rs2941484
rs6598541
rs10480300
rs7193778
MR-Egger estimate
Weighted median estimate
IVW estimate
Variants
-2
-1
0
1
2
Causal estimate (95% CI)

k)

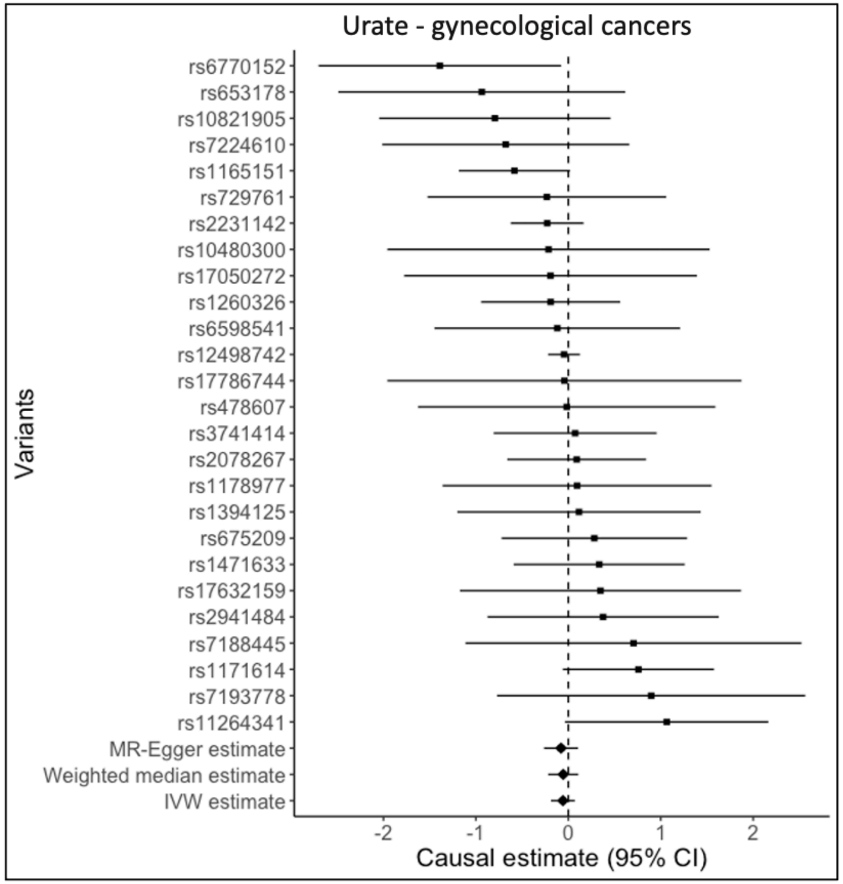

Urate - gynecological cancers
rs6770152
rs653178
rs10821905
rs7224610
rs1165151
rs729761
rs2231142
rs10480300
rs17050272
rs1260326
rs6598541
rs12498742
rs17786744
rs478607
rs3741414
rs2078267
rs1178977
rs1394125
rs675209
rs1471633
rs17632159
rs2941484
rs7188445
rs1171614
rs7193778
rs11264341
MR-Egger estimate
Weighted median estimate
IVW estimate
Variants
-2
-1
0
1
2
Causal estimate (95% CI)

l)

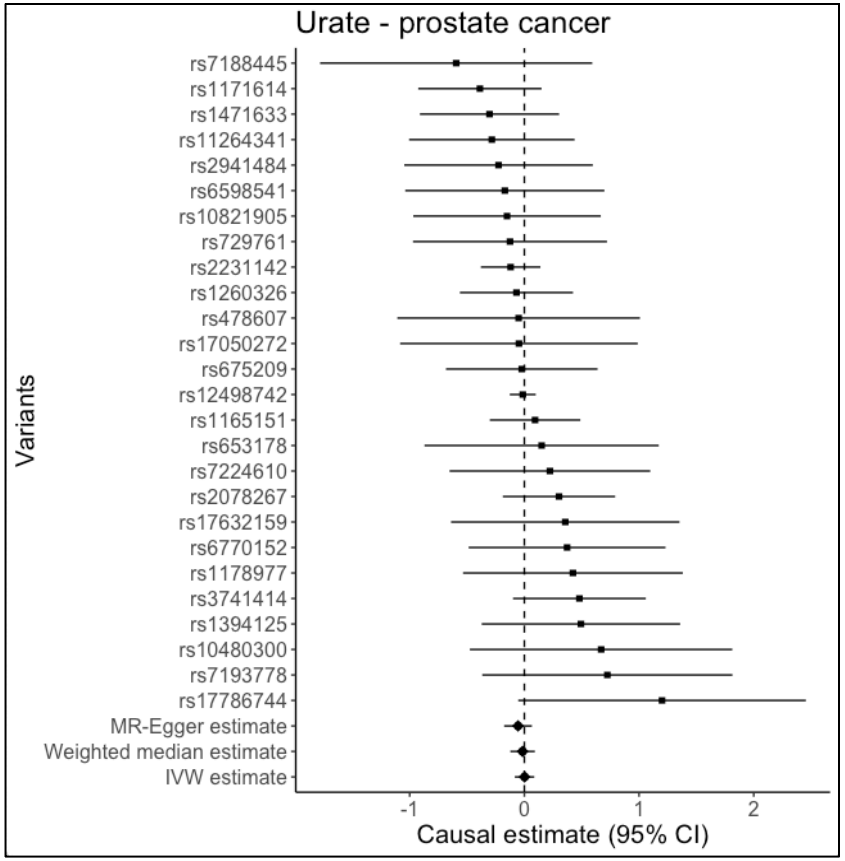
Urate - prostate cancer
rs7188445
rs1171614
rs1471633
rs11264341
rs2941484
rs6598541
rs10821905
rs729761
rs2231142
rs1260326
rs478607
rs17050272
rs675209
rs12498742
rs1165151
rs653178
rs7224610
rs2078267
rs17632159
rs6770152
rs1178977
rs3741414
rs1394125
rs10480300
rs7193778
rs17786744
MR-Egger estimate
Weighted median estimate
IVW estimate
Variants
-1
0
1
2
Causal estimate (95% CI)

m)

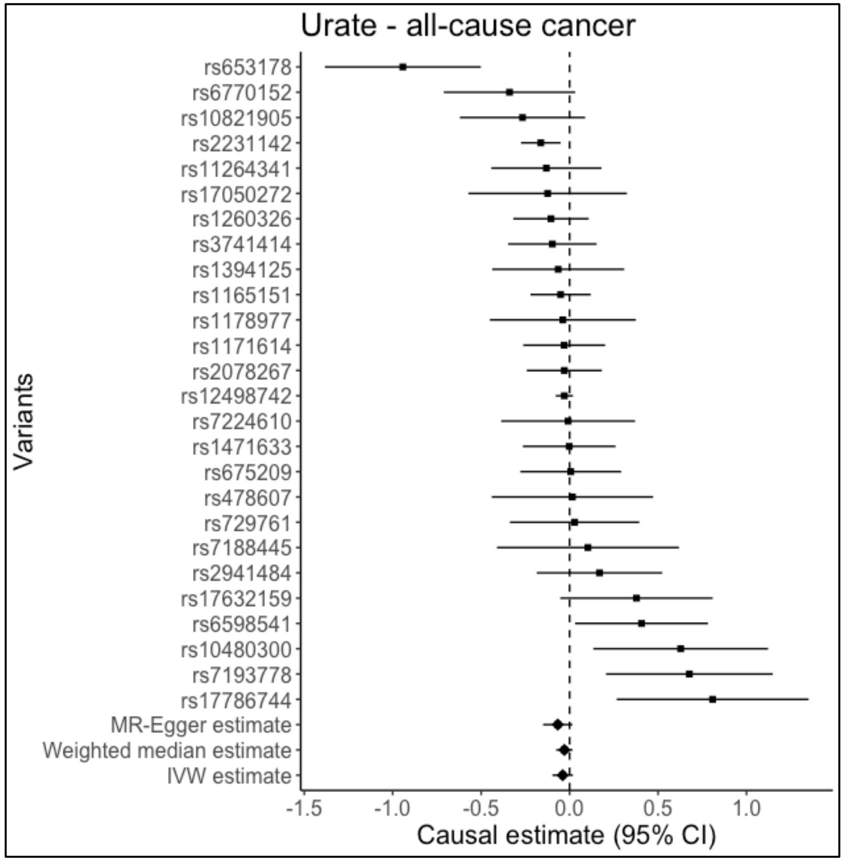
Urate - all-cause cancer
rs653178
rs6770152
rs10821905
rs2231142
rs11264341
rs17050272
rs1260326
rs3741414
rs1394125
rs1165151
rs1178977
rs1171614
rs2078267
rs12498742
rs7224610
rs1471633
rs675209
rs478607
rs729761
rs7188445
rs2941484
rs17632159
rs6598541
rs10480300
rs7193778
rs17786744
MR-Egger estimate
Weighted median estimate
IVW estimate
Variants
-1.5
-1.0
-0.5
0.0
0.5
1.0
Causal estimate (95% CI)

n)

Figure S2 (a to n): Forest plots of leave-one-out analyses for the estimated MR effects of serum urate levels on the risk of different cancer types

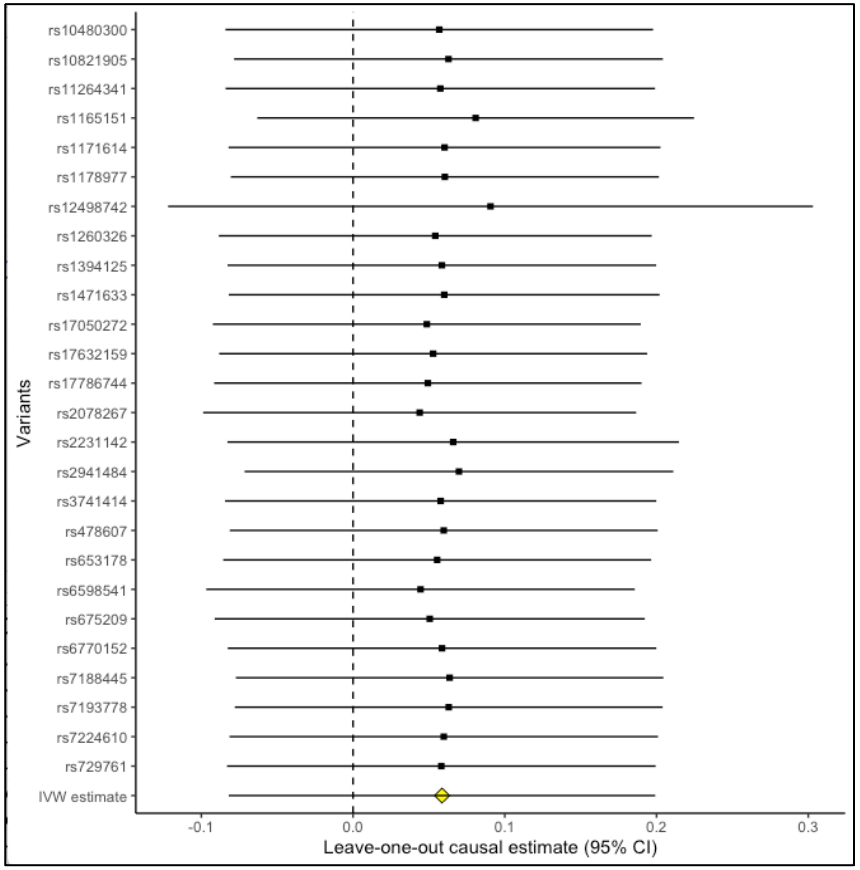

a) Urate - bladder cancer

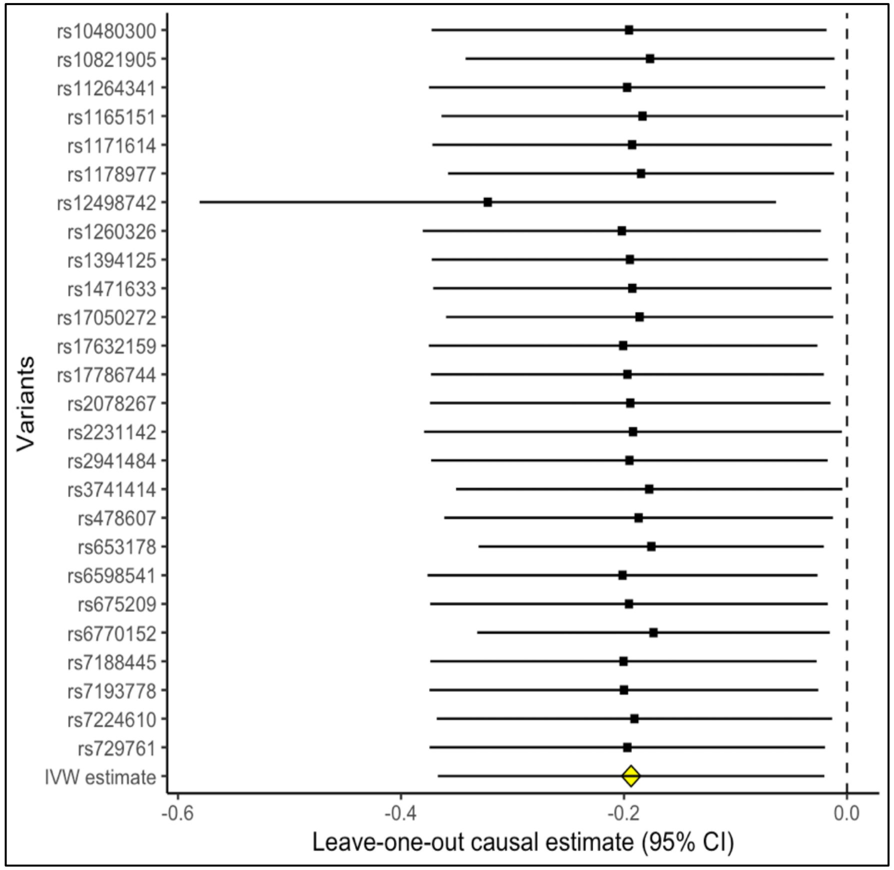

b) Urate - colorectal cancer

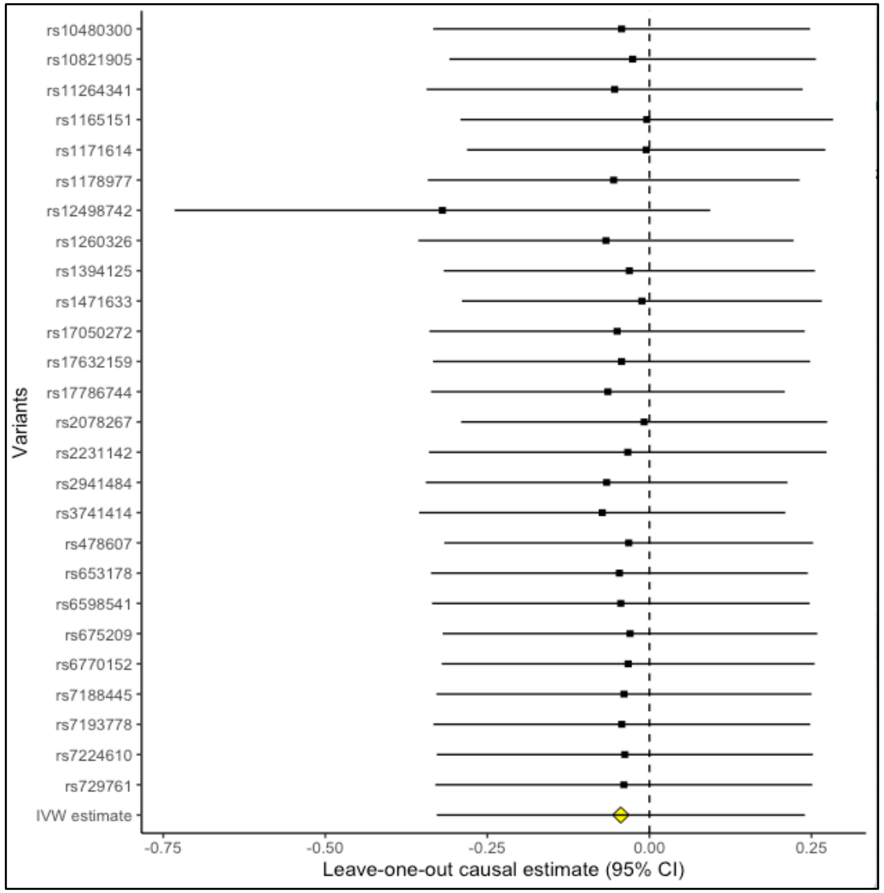

c) Urate - brain tumor

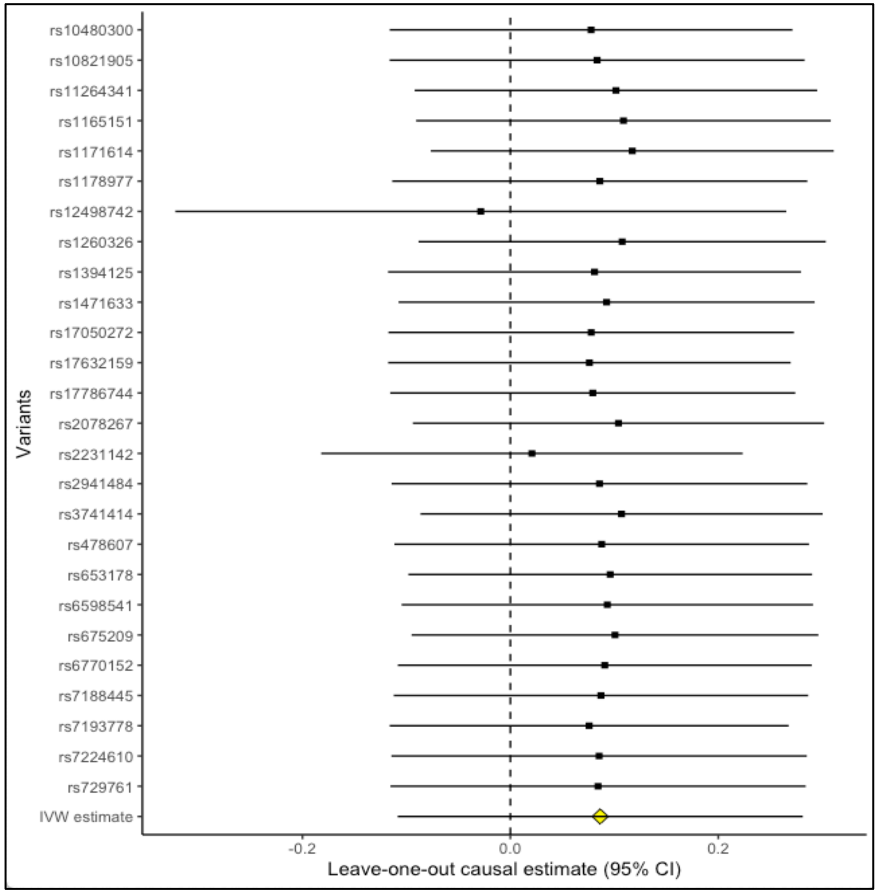

d) Urate - gastric cancer

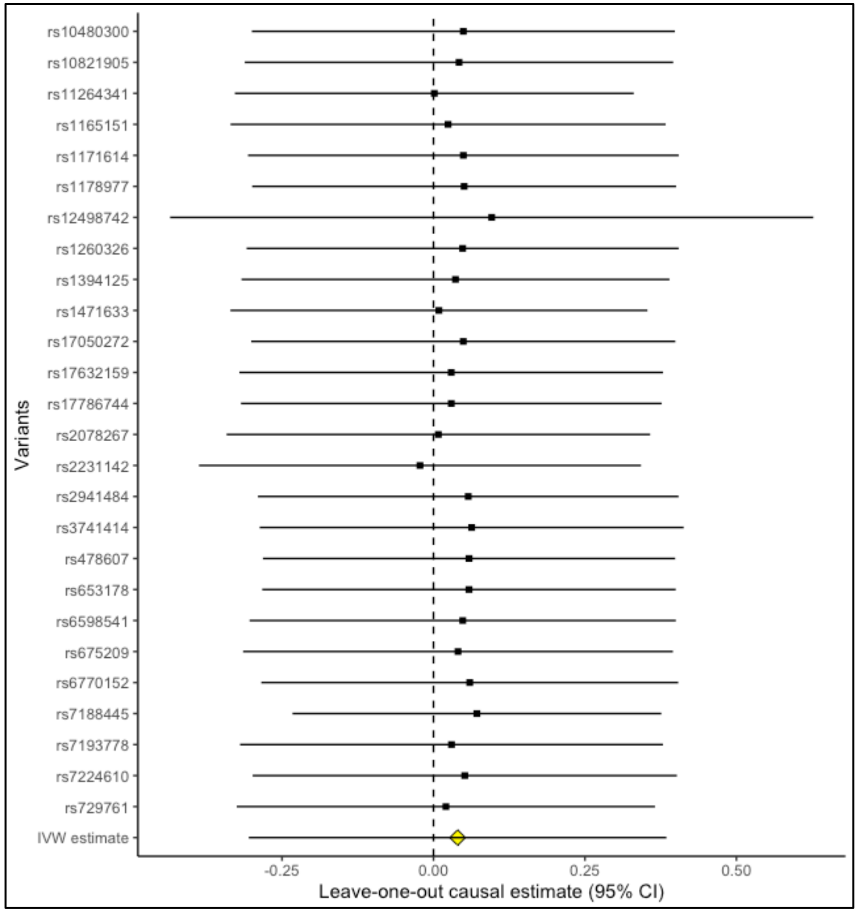

e) Urate - hepatic cancer

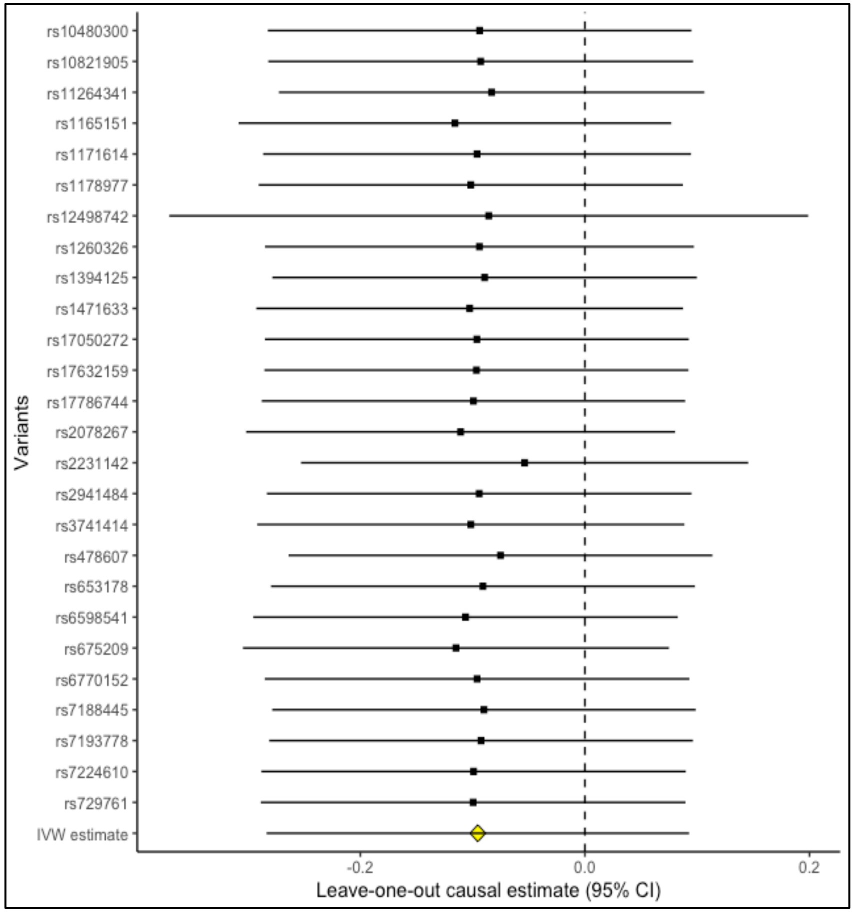

f) Urate - renal cancer

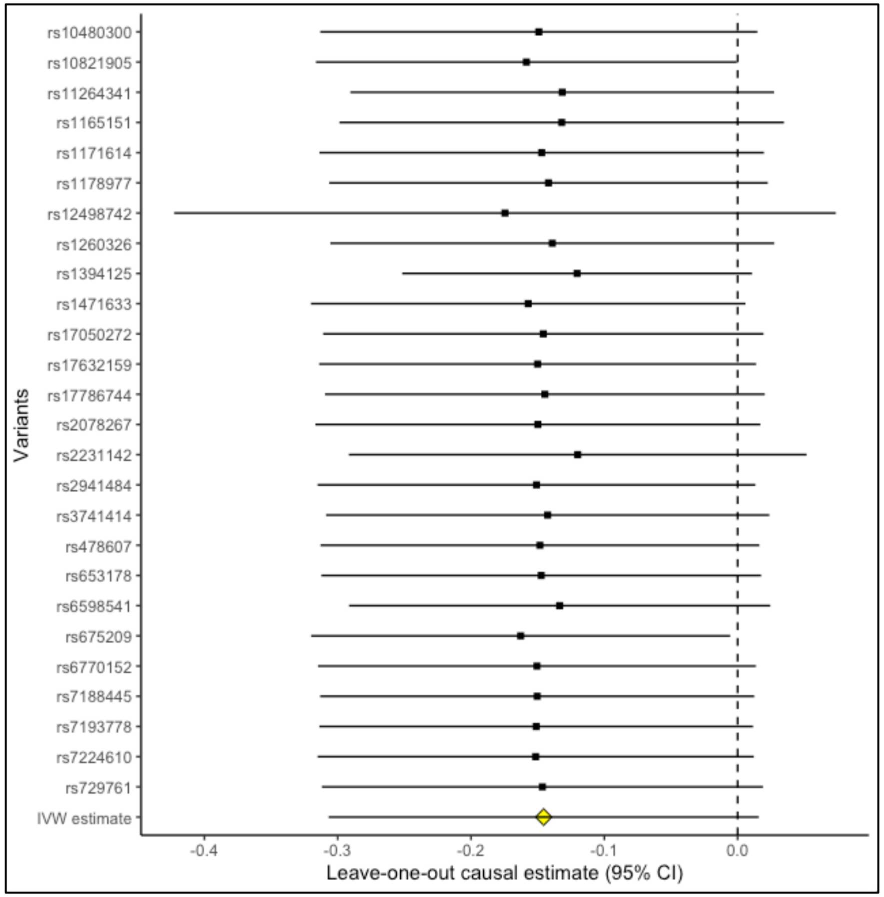

g) Urate - lung cancer

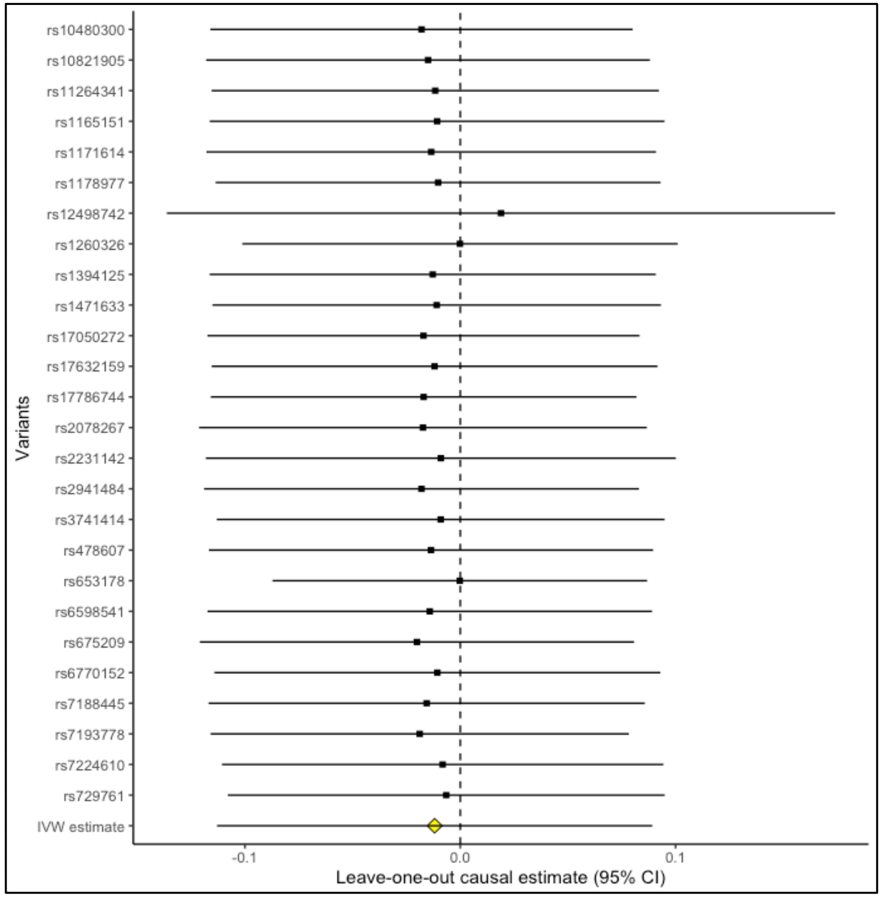

h) Urate - lymphatic and hematopoietic cancer

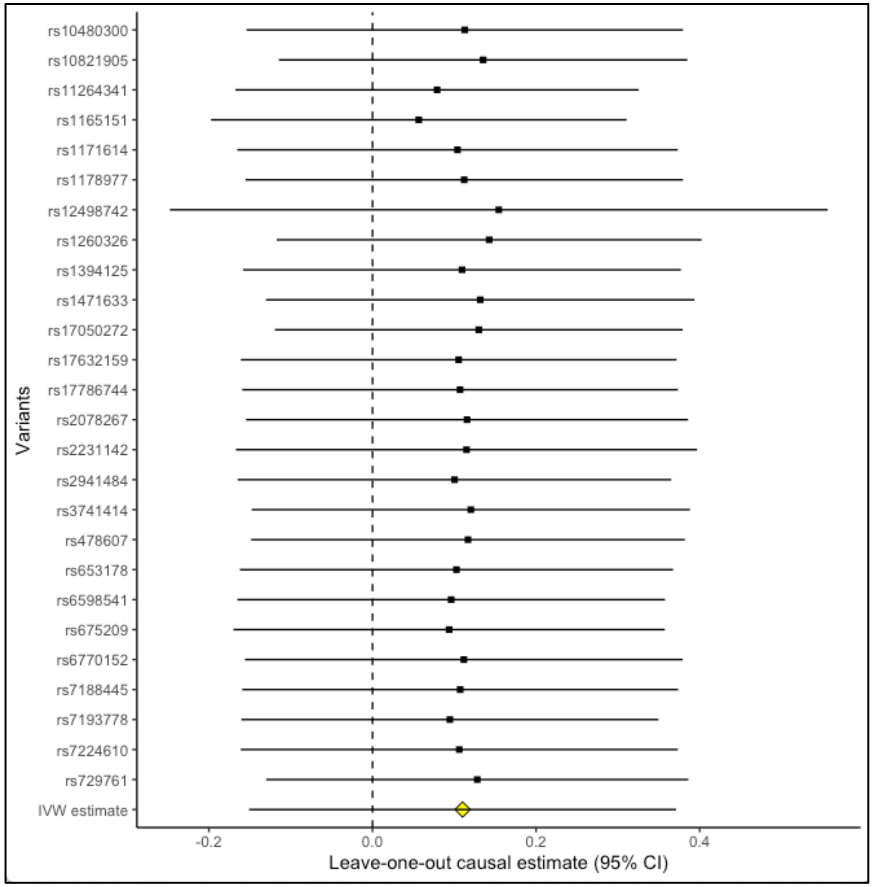

i) Urate - pancreatic cancer

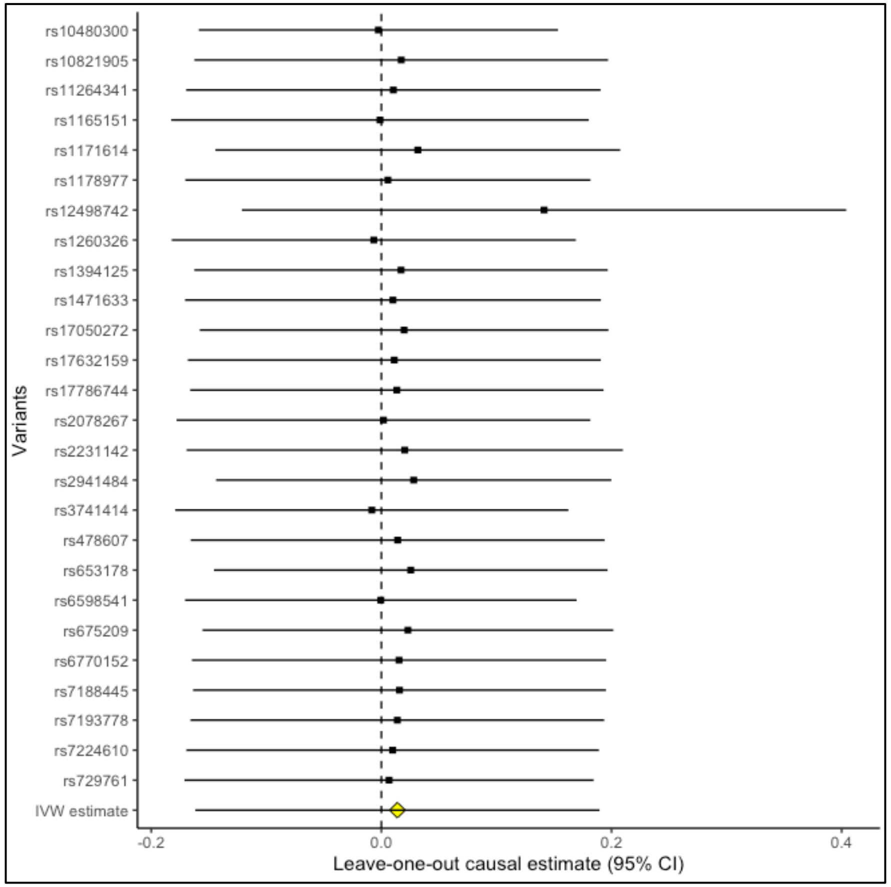

j) Urate - skin cancer

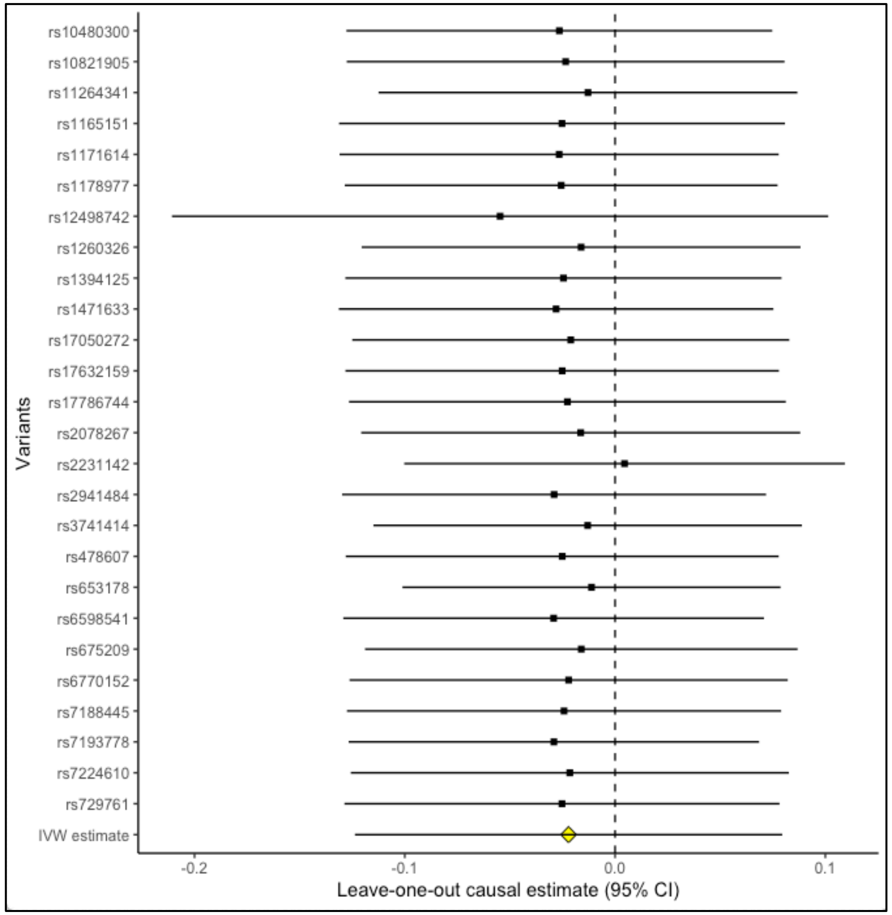

k) Urate – breast cancer

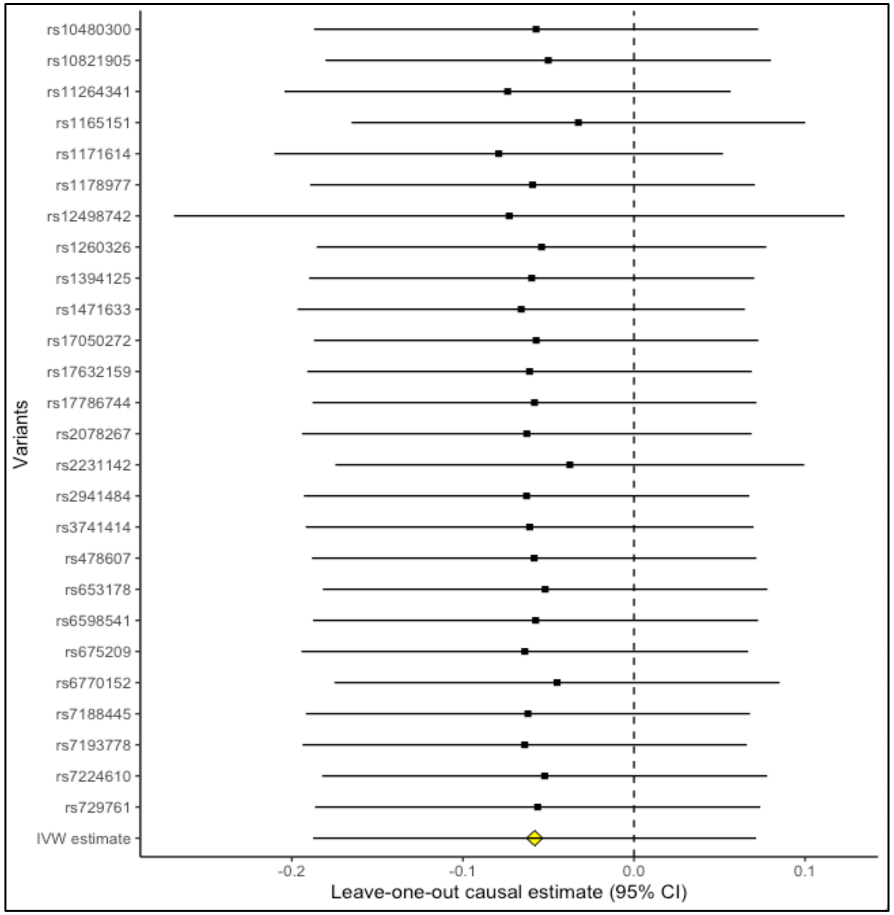

l) Urate - gynecological cancers

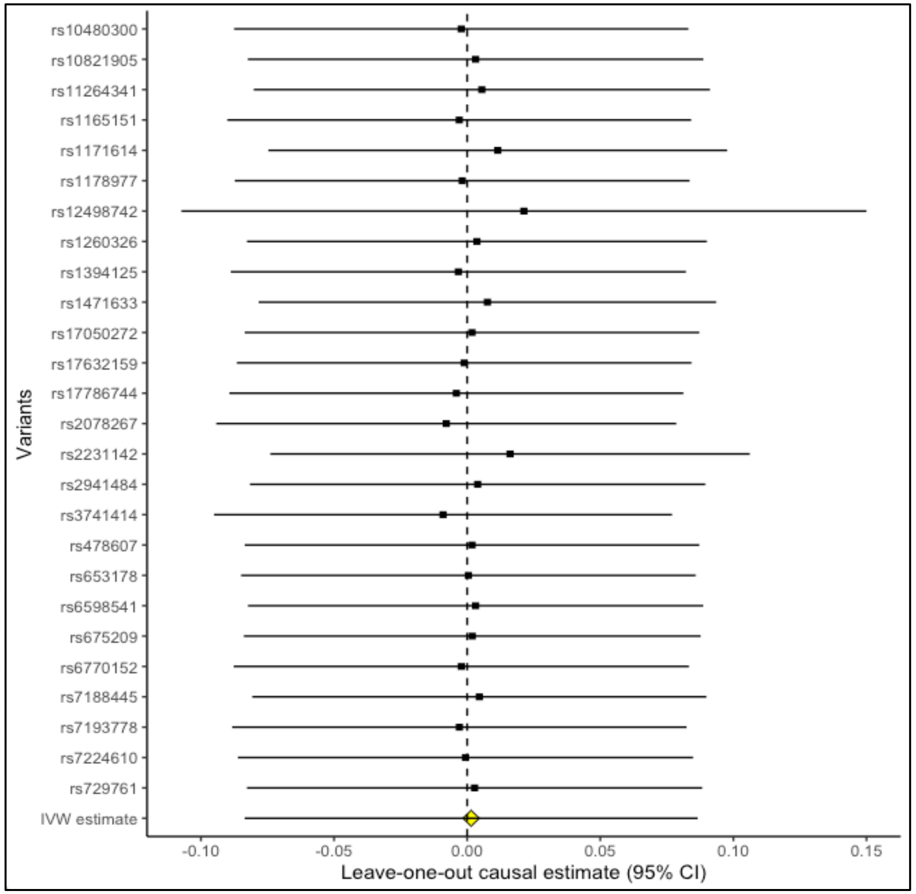

m) Urate - prostate cancer

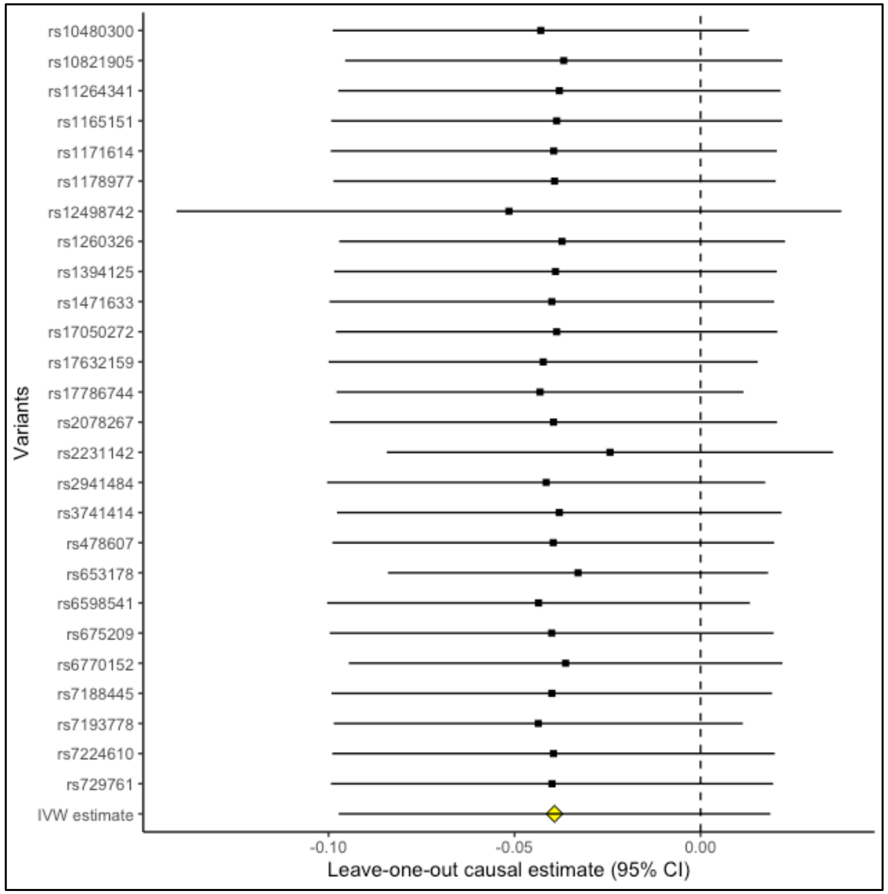

n) Urate - all-cause cancer

REFERENCES

1. Teleka S, Hindy G, Drake I, et al. Blood pressure and bladder cancer risk in men by use of survival analysis and in interaction with NAT2 genotype, and by Mendelian randomization analysis. *PloS one*. 2020;15:e0241711.
2. Bycroft C, Freeman C, Petkova D, et al. The UK Biobank resource with deep phenotyping and genomic data. *Nature*. 2018;562:203-9.
3. Köttgen A, Albrecht E, Teumer A, et al. Genome-wide association analyses identify 18 new loci associated with serum urate concentrations. *Nature genetics*. 2013;45:145-54.
4. Palmer TM, Lawlor DA, Harbord RM, et al. Using multiple genetic variants as instrumental variables for modifiable risk factors. *Statistical methods in medical research*. 2012;21:223-42.
5. Teslovich TM, Musunuru K, Smith AV, et al. Biological, clinical and population relevance of 95 loci for blood lipids. *Nature*. 2010;466:707-13.
6. Staley JR, Blackshaw J, Kamat MA, et al. PhenoScanner: a database of human genotype–phenotype associations. *Bioinformatics*. 2016;32:3207-9.
